# Downregulation of a Dorsal Root Ganglion‐Specifically Enriched Long Noncoding RNA is Required for Neuropathic Pain by Negatively Regulating RALY‐Triggered Ehmt2 Expression

**DOI:** 10.1002/advs.202004515

**Published:** 2021-05-14

**Authors:** Zhiqiang Pan, Shibin Du, Kun Wang, Xinying Guo, Qingxiang Mao, Xiaozhou Feng, Lina Huang, Shaogen Wu, Bailing Hou, Yun‐Juan Chang, Tong Liu, Tong Chen, Hong Li, Thomas Bachmann, Alex Bekker, Huijuan Hu, Yuan‐Xiang Tao

**Affiliations:** ^1^ Department of Anesthesiology New Jersey Medical School, Rutgers The State University of New Jersey Newark NJ 07103 USA; ^2^ The Office of Advanced Research Computing Rutgers The State University of New Jersey Newark NJ 07103 USA; ^3^ Center for Advanced Proteomics Research Departments of Biochemistry, Microbiology & Molecular Genetics New Jersey Medical School, Rutgers The State University of New Jersey Newark NJ 07103 USA; ^4^ Department of Physiology, Pharmacology & Neuroscience New Jersey Medical School Rutgers The State University of New Jersey Newark NJ 07103 USA; ^5^ Department of Cell Biology & Molecular Medicine New Jersey Medical School Rutgers The State University of New Jersey Newark NJ 07103 USA

**Keywords:** Dorsal root ganglion, DRG‐specifically enriched long noncoding RNA, *Ehmt2*, G9a, Neuropathic pain, RALY

## Abstract

Nerve injury‐induced maladaptive changes of gene expression in dorsal root ganglion (DRG) neurons contribute to neuropathic pain. Long non‐coding RNAs (lncRNAs) are emerging as key regulators of gene expression. Here, a conserved lncRNA is reported, named DRG‐specifically enriched lncRNA (*DS‐lncRNA*) for its high expression in DRG neurons. Peripheral nerve injury downregulates *DS‐lncRNA* in injured DRG due, in part, to silencing of POU domain, class 4, transcription factor 3, a transcription factor that interacts with the DS‐lncRNA gene promoter. Rescuing *DS‐lncRNA* downregulation blocks nerve injury‐induced increases in the transcriptional cofactor RALY‐triggered DRG *Ehmt2* mRNA and its encoding G9a protein, reverses the G9a‐controlled downregulation of opioid receptors and Kcna2 in injured DRG, and attenuates nerve injury‐induced pain hypersensitivities in male mice. Conversely, *DS‐lncRNA* downregulation increases RALY‐triggered *Ehmt2* */*G9a expression and correspondingly decreases opioid receptor and Kcna2 expression in DRG, leading to neuropathic pain symptoms in male mice in the absence of nerve injury. Mechanistically, downregulated *DS‐lncRNA* promotes more binding of increased RALY to RNA polymerase II and the *Ehmt2* gene promoter and enhances *Ehmt2* transcription in injured DRG. Thus, downregulation of *DS‐lncRNA* likely contributes to neuropathic pain by negatively regulating the expression of RALY‐triggered *Ehmt2*/G9a, a key neuropathic pain player, in DRG neurons.

## Graphical abstract

This work reports that nerve trauma‐induced the downregulation of a dorsal root ganglion‐specifically enriched long noncoding RNA (*DS‐lncRNA*) promotes the binding of the transcription co‐factor RALY to RNA polymerase II (RNP II) and the *Ehmt2* gene promoter, increases the expression of *Ehmt2* mRNA and G9a protein, downregulates many pain‐associated genes including *Oprm1* mRNA and mu opioid receptor (MOR), and causes neuropathic pain.

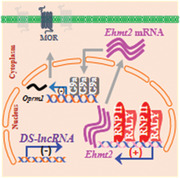

## 1 Introduction

Chronic neuropathic pain is a major public health problem affecting the quality of life of approximately 7% of the world's population.^[^ ^1^, ^2^ ^]^ In the United States, an estimated 600 billion dollars per year are spent on neuropathic pain‐associated healthcare costs and productivity losses.^[^ ^3^ ^]^ Current treatments for this debilitating disorder have limited effectiveness and/or produce severe adverse effects.^[^ ^4^, ^5^ ^]^ Neuropathic pain is characterized by intermittent burning pain, spontaneous ongoing pain, hyperalgesia (an enhanced response to noxious stimuli), and allodynia (pain in response to normally innocuous stimuli). These pain hypersensitivities are thought to be triggered at least in part by abnormal hyperexcitability and ectopic discharges that arise in neuromas and first‐order sensory neurons of injured dorsal root ganglia (DRG).^[^ ^4^, ^6^, ^7^, ^8^ ^]^ Peripheral nerve injury‐induced maladaptive changes in pain‐associated genes at transcriptional and translational levels in the DRG contribute to these abnormal spontaneous activities and subsequent increased neurotransmitter release from the primary afferents of DRG neurons.^[^ ^9^, ^10^, ^11^, ^12^, ^13^, ^14^, ^15^, ^16^, ^17^, ^18^ ^]^ Understanding the regulation of these DRG genes may provide a new avenue for neuropathic pain management.

The euchromatic histone lysine methyltransferase 2 (*Ehmt2*) gene encodes the G9a protein, a repressor of gene transcription through its dimethylation of histone H3 at Lys^[^ ^9^ ^]^ and subsequent condensation of chromatin.^[^ ^19^, ^20^ ^]^ Levels of both *Ehmt2* mRNA and G9a protein are increased in injured DRG following peripheral nerve injury.^[^ ^9^, ^12^, ^13^, ^21^ ^]^ Blocking this increase through genetic knockdown/knockout of DRG *Ehmt2* or pharmacological inhibition of DRG G9a decreased DRG neuronal hyperexcitability, impaired pain hypersensitivities, rescued opioid analgesia, and blocked opioid analgesic tolerance induction under neuropathic pain conditions.^[^ ^9^, ^12^, ^13^, ^21^, ^22^ ^]^ These effects are likely attributed to G9a participation in nerve injury‐induced downregulation of many pain‐associated genes (e.g., opioid receptors and several voltage‐gated potassium channel genes) in injured DRG.^[^ ^9^, ^12^, ^13^, ^21^ ^]^ *Ehmt2*/G9a is thus a likely key initiator in neuropathic pain genesis. However, how peripheral nerve injury transcriptionally activates *Ehmt2* in injured DRG remains elusive.

Long non‐coding RNAs (lncRNAs) are emerging as potent and multifunctional regulators of gene expression.^[^ ^23^, ^24^ ^]^ A large number of lncRNAs are dysregulated in DRG following peripheral nerve injury,^[^ ^25^, ^26^ ^]^ but the role of most of them in neuropathic pain is still incompletely understood.^[^ ^27^, ^28^, ^29^, ^30^, ^31^, ^32^, ^33^, ^34^ ^]^ Here, we report a unique and native lncRNA highly expressed in mammalian DRG, named DRG‐specifically enriched lncRNA (*DS‐lncRNA*), and find that peripheral nerve injury downregulates its expression in injured DRG. This downregulation is required for nerve injury‐induced neuropathic pain development and maintenance by negatively regulating RALY (a transcriptional cofactor^[^ ^35^ ^]^)/RNA polymerase II (RNP II)‐triggered *Ehmt2* gene expression in injured DRG. DS‐lncRNA is thus a likely key player in nerve injury‐induced pain hypersensitivity.

## 2 Results

### 2.1 Identification of *DS‐lncRNA* in DRG Neurons

**Figure 1:**
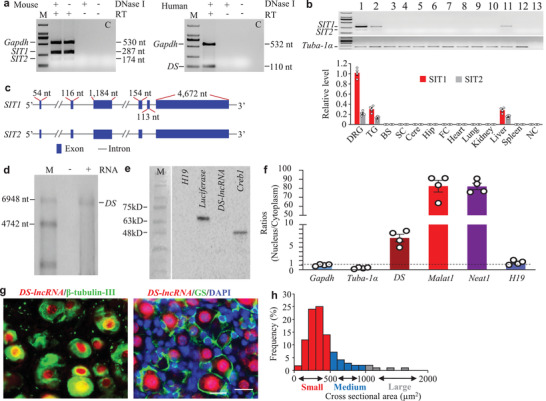
Identification of dorsal root ganglion (DRG)‐specifically‐enriched lncRNA (*DS‐lncRNA*) in DRG neurons. a) A long and highly expressed splice isoform transcript I (*SIT1*) and a short and weakly expressed splice isoform transcript II (*SIT2*) of *DS‐lncRNA* were detected in mouse DRG and one *DS‐lncRNA* (DS) transcript was detected in human DRG using reverse transcription (RT)‐PCR with strand‐specific primers. To exclude genomic DNA contamination, the extracted RNA samples were pretreated with excess DNase I. GAPDH was used as a control. Without RT primers, neither *Gapdh* nor *DS‐lncRNA* PCR products were detected in the DNase I‐treated samples. n = 3 biological repeats/species. Lane C: H_2_O. M: DNA ladder marker. b) Expression of *SIT1* and *SIT2* in different tissues from normal mice. Lane 1: dorsal root ganglion (DRG). Lane 2: trigeminal ganglion (TG). Lane 3: brainstem (BS). Lane 4: spinal cord (SC). Lane 5: cerebellum (Cere). Lane 6: hippocampus (Hip). Lane 7: Frontal cortex (FC). Lane 8: heart. Lane 9: lung. Lane 10: kidney. Lane 11: liver. Lane 12: spleen. Lane 13: no‐template control (NC). *Tuba‐1* *α* is used as an internal control. n = 4 mice. M: DNA ladder marker. c) Schematic diagrams of full‐length *SIT1* and *SIT2* constructions analyzed by 5′ and 3′ RACE assay. Blue‐highlighted boxes indicate exons and the linked thin lines introns. d) Northern blot analysis of *DS‐lncRNA* (*DS*) expression (lane 3) in mouse DRG. ‐: no RNA. +: with RNA. n = 3 mice. M: RNA marker. e) *In vitro* translation of *DS‐lncRNA* using the Transcend Non‐Radioactive Translation Detection Systems. *H19* is used as a control for noncoding RNA. Luciferase and *Creb1* are used as controls for coding RNA. M: protein molecular weight marker. n = 3 mice. f) Ratios of nucleus to cytoplasm for *Gapdh* mRNA, *Tuba‐1α* mRNA, *DS‐lncRNA*, *Malat1*, *Neat1*, and *H19* in cultured DRG neurons. n = 4 mice. g) *DS‐lncRNA* (red) was co‐expressed with *β*‐tubulin III (green, left) in individual DRG cells and undetected in cellular nuclei (labeled by 4′, 6‐diamidino‐2‐phenylindole (DAPI), blue, right) of glutamine synthetase (GS, green, right)‐labeled DRG cells. n = 5 mice. Scale bar: 50 µm. h) Histogram showing the distribution of *DS‐lncRNA*‐positive somata in the normal mouse L4 DRG: small, 77%; medium, 18%; large, 5%. n = 3 mice.

To identify *DS‐lncRNA*, we analyzed our previous RNA sequencing database^[^ ^26^ ^]^ and found that the stacked reads dramatically decreased in the genomic region (42456442–42461110) of chromosome 18 from the ipsilateral L4 DRG of unilateral L4 spinal nerve ligation (SNL) mice as compared to sham mice on day 7 post‐surgery (Figure S1, Supporting Information). We then designed strand‐specific primers for reverse transcription. A long and highly expressed splice isoform transcript I (*SIT1*) and a short and weakly expressed splice isoform transcript II (*SIT2*) were identified in mouse DRG (**Figure** **1**a; Figures S2,3, Supporting Information), whereas one transcript was detected in human DRG (Figure 1a), although the sequences between two species were not identical. In mice, both transcripts were also detected at low levels in the trigeminal ganglion and liver but were undetectable in the remaining body tissues (Figure 1b). By using 5′ and 3′ RACE and RT‐PCR assays, we identified the full‐length 6.293‐kb *SIT1* (including 6 exons) and 6.18‐kb *SIT2* (without exon 5) in mouse DRG (Figure 1c; Figures S2,3, Supporting Information). Northern blot analysis of RNA from adult mouse DRG confirmed *DS‐lncRNA* at the expected size although these two transcripts could not be distinguished due to the 0.113‐kb difference between them (Figure 1d). Coding substitution frequency analysis^[^ ^36^, ^37^ ^]^ of *DS‐lncRNA* predicted a negative/low protein‐coding potential (Figures S2,3, Supporting Information). Consistently, *in vitro* translation of *DS‐lncRNA* yielded no proteins (Figure 1e). Ribosome profiling analysis^[^ ^38^ ^]^ revealed no/minimal ribosomes on *DS‐lncRNA* (Figure S4a,b, Supporting Information). This evidence indicates that *DS‐lncRNA* is a non‐coding RNA.

Subcellular fractionation of DRG showed that *DS‐lncRNA*, like nuclear long noncoding RNA *Malat1*,^[^ ^39^ ^]^ was located mainly in the insoluble nuclear pellet (Figure S5a,b, Supporting Information). Quantification analysis of nuclear/cytoplasmic RNA from DRG extracts also revealed that *DS‐lncRNA* was distributed predominantly in nuclei (Figure 1f). In situ hybridization histochemistry assay showed that *DS‐lncRNA* was expressed highly in DRG neuronal nuclei, but not in adjacent satellite cells (Figure 1g). Approximately 73.7% of DRG neurons (252/342) were labeled, of which about 77% were small (< 25 µm in diameter), 18% medium (25–35 µm in diameter), and 5% large (> 35 µm in diameter) (Figure 1h). Consistently, approximately 37.7% of *DS‐lncRNA*‐labeled neurons were positive for isolectin B4 (IB4), 41.4% for calcitonin gene‐related peptide (CGRP) and 31.7% for neurofilament‐200 (NF‐200) (Figure S6, Supporting Information). Distribution patterns suggest that *DS‐lncRNA* may participate in the transmission/modulation of nociceptive information.

### 2.2 *DS‐lncRNA* Downregulation in Injured DRG After Peripheral Nerve Injury

**Figure 2:**
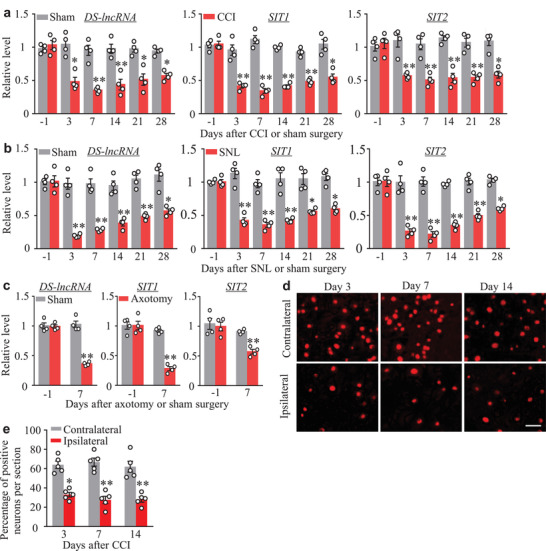
Downregulation of *DS‐lncRNA*, *SIT1*, and *SIT2* expression in injured dorsal root ganglion (DRG) after peripheral nerve injury. a) Levels of *DS‐lncRNA*, *SIT1*, and *SIT2* in the ipsilateral L3/4 DRGs after chronic constriction injury (CCI) or sham surgery of unilateral sciatic nerve. n = 8 mice/time point/group. *P < 0.05, **P < 0.01 versus the corresponding day ‐1 post‐surgery by two‐way ANOVA followed by post hoc Tukey test. b) Levels of *DS‐lncRNA*, ***SIT1***, and *SIT2* in the ipsilateral L4 DRG after unilateral spinal nerve ligation (SNL) or sham surgery. n = 16 mice/time point/group. *P < 0.05, **P < 0.01 versus the corresponding day ‐1 post‐surgery by two‐way ANOVA followed by post hoc Tukey test. c) Levels of *DS‐lncRNA*, *SIT1*, and *SIT2* in the ipsilateral L3/4 DRGs on day 7 after axotomy or sham surgery of unilateral sciatic nerve. n = 8 mice/time point/group. **P < 0.01versus the corresponding day ‐1 post‐surgery by two‐way ANOVA followed by post hoc Tukey test. d,e) In situ hybridization histochemistry demonstrating *DS‐lncRNA*‐labeled neurons in the contralateral and ipsilateral L4 DRG after CCI surgery (d) and their corresponding statistical analysis (e). Scale bar: 50 µm. n = 5 mice/time point/group. *P < 0.05, **P < 0.01 versus the corresponding sham group (0 day) by two‐way ANOVA followed by post hoc Tukey test.

We next examined whether the expression of *DS‐lncRNA* was altered in DRG after peripheral nerve injury. Levels of total *DS‐lncRNA* and its two transcripts were significantly reduced in the ipsilateral, but not contralateral, lumbar 3 and 4 (L3/4) DRGs on days 3, 7, 14, 21, and 28 following chronic constriction injury (CCI) of unilateral sciatic nerve, but not sham surgery (**Figure** **2**a; Figure S7, Supporting Information). Results were similar after SNL or unilateral sciatic nerve axotomy (Figure 2b, c; Figure S7, Supporting Information). Furthermore, the number of *DS‐lncRNA*‐labeled neurons in the ipsilateral L3/4 DRGs was significantly less than that in the contralateral L3/4 DRGs on days 3, 7, and 14 post‐CCI (Figure 2d,e). It thus appears that peripheral nerve injury down‐regulates *DS‐lncRNA* in injured DRG neurons.

### 2.3 Pou4f3 Participates in CCI‐Induced *DS‐lncRNA* Downregulation

**Figure 3:**
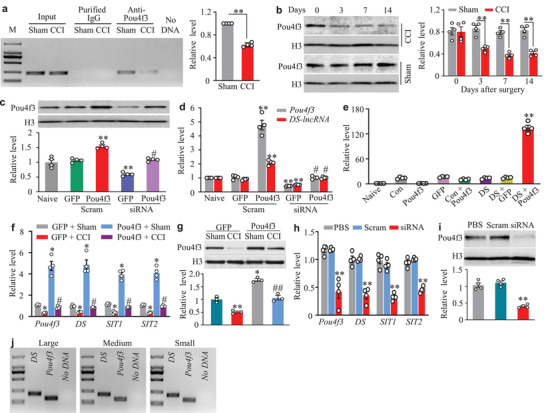
*DS‐lncRNA* downregulation caused by Pou4f3 silence in injured DRG following peripheral nerve injury. a) The *DS‐lncRNA* promoter fragment immunoprecipitated by rabbit anti‐Pou4f3 antibody in the ipsilateral L3/4 DRGs on day 7 after CCI or sham surgery. Input: total purified fragments. IgG: purified rabbit IgG. M: DNA ladder marker. n = 12 mice/group. **P < 0.01 versus the sham group by two‐tailed unpaired Student's *t*‐test. b) Pou4f3 protein expression in the ipsilateral L3/4 DRGs after CCI or sham surgery. n = 8 mice/time point/group. **P < 0.01 versus the corresponding sham group (0 day) by two‐way ANOVA followed by post hoc Tukey test. c,d) Levels of Pou4f3 protein (c), *Pou4f3* mRNA (d) and *DS‐lncRNA* (d) in cultured DRG neurons transfected/transduced as shown. Naïve: no treatment. Pou4f3: AAV5 expressing full‐length *Pou4f3*. GFP: AAV5 expressing *Gfp*. siRNA: Pou4f3 siRNA. Scram: control scrambled siRNA. n = 8 mice/treatment. **P < 0.01 versus the naïve group and #*P* < 0.05 versus the GFP plus siRNA group by one‐way ANOVA followed by post hoc Tukey test. e) *DS‐lncRNA* promoter activities in HEK‐293T cells transfected as shown. Naïve: no treatment. Con: control vector (pGL3‐Basic). Pou4f3: proviral vector (AAV5) expressing full‐length *Pou4f3*. GFP, proviral vector expressing *Gfp*. DS: pGL3 reporter vector with *DS‐lncRNA* promoter. n = 4 biological repeats/treatment. **P < 0.01 versus the DS plus GFP‐treated group by one‐way ANOVA followed by post hoc Tukey test. f) Levels of *Pou4f3* mRNA (Pou4f3), *DS‐lncRNA* (DS), *SIT1*, and *SIT2* in the ipsilateral L3/4 DRGs on day 14 after CCI or sham surgery in mice pre‐microinjected with AAV5‐Gfp (GFP) or AAV5‐Pou4f3 (Pou4f3) into the ipsilateral L3/4 DRGs 5 weeks before CCI or sham surgery. n = 8 mice/group. *P < 0.05 versus the corresponding GFP‐treated sham group and #*P* < 0.05 versus the corresponding GFP‐treated CCI group by one‐way ANOVA followed by post hoc Tukey test. g) Levels of Pou4f3 protein in the ipsilateral L3/4 DRGs on day 14 after CCI or sham surgery in mice pre‐microinjected with AAV5‐Gfp or AAV5‐Pou4f3 into unilateral L3/4 DRGs for 35 days. n = 6 mice/group. *P < 0.05, **P < 0.01 versus the AAV5‐Gfp plus sham group and ##*P* < 0.01 versus the AAV5‐Gfp plus CCI group by one‐way ANOVA followed by post hoc Tukey test. h) Levels of *Pou4f3* mRNA (Pou4f3), *DS‐lncRNA* (DS), *SIT1*, and *SIT2* in the ipsilateral L3/4 DRGs on day 8 after DRG microinjection of Pou4f3 siRNA (siRNA), control scrambled siRNA (Scram) or PBS. n = 8 mice/group. **P < 0.01 versus the corresponding PBS group by one‐way ANOVA followed by post hoc Tukey test. i) Level of Pou4f3 protein in the ipsilateral L3/4 DRG on day 8 after microinjection with PBS, Pou4f3‐siRNA (siRNA), or scrambled siRNA (Scram). n = 8 mice/group. **P < 0.01 versus the PBS group by one‐way ANOVA followed by post hoc Tukey test. j) Co‐expression of *Pou4f3*mRNA with *DS‐lncRNA* in individual small, medium, and large DRG neurons. n = 3 mice.

How is DRG *DS‐lncRNA* downregulated after nerve injury? We found a transcriptional activator POU domain, class 4, transcription factor 3 (*Pou4f3*) gene located adjacent and upstream (−3818 to −2322; DS‐lncRNA gene's TTS as +1) of the *DS‐lncRNA* gene promoter. In addition, *Pou4f3* expression in an RNA sequencing database was decreased in injured DRG after SNL.^[^ ^26^ ^]^ Thus, we predicted that Pou4f3 could regulate the expression of the *DS‐lncRNA* gene. Using the online software JASPAR, we found two potential Pou4f3‐binding regions including region −81 to −96 (score: 11.3125) and region −190 to −205 (score: 11.0054) in the negative strand of the *DS‐lncRNA* promoter, both of which contained one consensus binding motif (5′‐ATGCAATA‐3′) for Pou4f3. We chose the region with the highest score for the following experiments. A chromatin immunoprecipitation (ChIP) assay revealed that a fragment of the *DS‐lncRNA* promoter containing this binding motif could be amplified from the complex immunoprecipitated with Pou4f3 antibody in nuclear fractions from sham DRG (**Figure** **3**a). CCI reduced the binding of Pou4f3 to *DS‐lncRNA* promoter, as evidenced by a 37.4% decrease in band density in the ipsilateral L3/4 DRG from the CCI mice compared to that from the sham mice on day 7 (Figure 3a). This decrease was attributed to a marked reduction of Pou4f3 in the ipsilateral L3/4 DRG following CCI (Figure 3b).

We further confirmed a Pou4f3 knockdown‐caused decrease in *DS‐lncRNA* in cultured DRG neurons that were transfected with *Pou4f3* siRNA (but not control scrambled siRNA) and transduced with AAV5 expressing control *Gfp* (AAV5‐Gfp; Figure 3c,d). This decrease was abolished in DRG neurons co‐transduced with AAV5 expressing full‐length *Pou4f3* mRNA (AAV5‐Pou4f3; Figure 3c,d). As expected, AAV5‐Pou4f3 transduction markedly upregulated the expression of *DS‐lncRNA* in the scrambled siRNA‐treated DRG neurons (Figure 3c,d). The luciferase assay revealed that co‐transfection of full‐length Pou4f3 vector, but not control Gfp vector, significantly increased the activity of the *DS‐lncRNA* gene promoter in *in vitro* HEK‐293 cells (Figure 3e). Furthermore, rescuing the CCI‐induced downregulation of DRG Pou4f3 through DRG pre‐microinjection of AAV5‐Pou4f3 (but not AAV5‐Gfp) not only attenuated the induction of CCI‐induced mechanical, heat, and cold nociceptive hypersensitivities (Figure S8, Supporting Information), but also reversed the CCI‐induced decreases in *DS‐lncRNA*, *SIT1*, and *SIT2* in injured L3/4 DRGs (Figure 3f,g). Additionally, DRG knockdown of Pou4f3 through DRG microinjection of *Pou4f3* siRNA (but not scrambled siRNA) reduced the levels of *DS‐lncRNA*, *SIT1*, and *SIT2* in the injected L3/4 DRGs and augmented responses to mechanical, heat, and cold stimuli after siRNA microinjection (Figure 3h,i; Figure S9, Supporting Information). Given that *Pou4f3* mRNA co‐expressed with *DS‐lncRNA* in individual small, medium, and large DRG neurons (Figure 3j) and that both of them had similar distribution patterns in DRG (Figure 1g, Figures S6, S10, Supporting Information), our data strongly support the idea that nerve injury‐induced *DS‐lncRNA* downregulation is due, at least in part, to silencing of Pou4f3 expression in injured DRG.

### 2.4 Rescuing Downregulated DRG *DS‐lncRNA* Mitigates Neuropathic Pain

**Figure 4:**
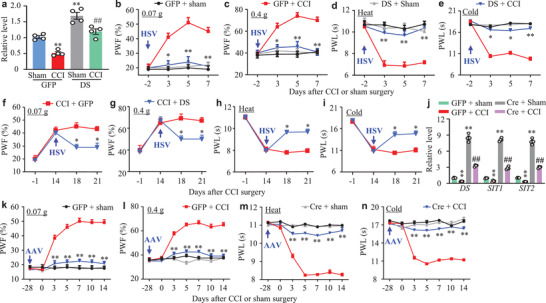
Rescuing nerve injury‐induced *DS‐lncRNA* downregulation in injured DRG attenuated neuropathic pain induction and maintenance. a) *DS‐lncRNA* expression in the ipsilateral L3/4 DRGs on day 7 after CCI or sham surgery in mice pre‐microinjected with HSV‐Gfp (GFP) or HSV‐DS‐lncRNA (DS) into the ipsilateral L3/4 DRGs 2 days before surgery. n = 8 mice/group. **P < 0.01 versus the GFP plus sham group and ##*P* < 0.01 versus the GFP plus CCI group by one‐way ANOVA followed by post hoc Tukey test. b–e) Effect of pre‐microinjection of HSV‐DS‐lncRNA (DS) or HSV‐Gfp (GFP) into unilateral L3/4 DRGs on the paw withdrawal frequency (PWF) to 0.07 g (b) and 0.4 g (c) von Frey filaments and on paw withdrawal latencies (PWL) to heat (d) and cold (e) stimuli on the ipsilateral side at the different days after CCI or sham surgery. n = 10 mice/group. *P < 0.05, **P < 0.01 versus the GFP plus CCI group at the corresponding time points by two‐way ANOVA with repeated measures followed by post hoc Tukey test. f–i) Effect of post‐microinjection of HSV‐DS‐lncRNA (DS) or HSV‐Gfp (GFP) into unilateral L3/4 DRGs on the paw withdrawal frequency (PWF) to 0.07 g (f) and 0.4 g (g) von Frey filaments and on paw withdrawal latencies (PWL) to heat (h) and cold (i) stimuli on the ipsilateral side at the different days after CCI surgery. n = 10 mice/group. *P < 0.05 versus the CCI plus GFP group at the corresponding time points by two‐way ANOVA with repeated measures followed by post hoc Tukey test. j) Levels of *DS‐lncRNA*, *SIT1*, and *SIT2* in the ipsilateral L3/4 DRGs on day 14 after CCI or sham surgery in conditional Rosa26^DS‐lncRNA^ knock‐in mice (DS‐KI mice) with pre‐microinjection of AAV5‐Cre (Cre) or AAV5‐Gfp (GFP) into the ipsilateral L3/4 DRGs 28 days before surgery. n = 8 mice/group. **P < 0.01 versus the corresponding GFP‐treated sham group and ##*P* < 0.01 versus the corresponding GFP‐treated CCI group by one‐way ANOVA followed by post hoc Tukey test. k–n) Effect of pre‐microinjection of AAV5‐Cre (Cre) or AAV5‐Gfp (GFP) into the ipsilateral L3/4 DRGs of DS‐KI mice on the paw withdrawal frequency (PWF) to 0.07 g (k) and 0.4 g (l) von Frey filaments and on paw withdrawal latencies (PWL) to heat (m) and cold (n) stimuli on the ipsilateral side at the different days after CCI or sham surgery. n = 12 mice/group. **P < 0.01 versus the GFP plus CCI group at the corresponding time points by two‐way ANOVA with repeated measures followed by post hoc Tukey test.

To examine whether DRG *DS‐lncRNA* downregulation participates in neuropathic pain genesis, we rescued its downregulation through microinjection of herpes simplex virus (HSV) expressing full‐length *DS‐lncRNA* (HSV‐lncRNA) into the ipsilateral L3/4 DRGs 2 days before CCI or sham surgery (**Figure** **4**a). HSV‐Gfp was used as a control. Consistent with previous studies,^[^ ^10^, ^18^, ^40^, ^41^ ^]^ CCI led to mechanical allodynia (demonstrated by increased paw withdrawal frequencies in response to low (0.07 g) and medium (0.4 g) force von Frey filaments), heat hyperalgesia, and cold allodynia (evidenced by decreased paw withdrawal latencies in response to heat and cold stimuli, respectively) on the ipsilateral side of HSV‐Gfp‐injected mice from days 3 to 7 after CCI (Figure 4b–e). These pain hypersensitivities did not develop in the CCI mice pre‐microinjected with HSV‐lncRNA during the observation period (Figure 4b–e). Microinjection of neither virus altered basal paw responses to mechanical, heat, and cold stimuli on the contralateral side of the CCI mice and on either side of the sham mice (Figure 4b–e; Figure S11a–c, Supporting Information). The role of DRG *DS‐lncRNA* downregulation in the maintenance of neuropathic pain was also observed. DRG microinjection of HSV‐lncRNA on day 14 post‐CCI (at this time point nociceptive hypersensitivities were completely established) significantly attenuated mechanical allodynia, heat hyperalgesia, and cold allodynia on days 18 and 21 post‐CCI on the ipsilateral side (Figure 4f–i). As expected, basal paw responses on the contralateral side (Figure S11d–f, Supporting Information) and locomotor functions (Table S1, Supporting Information) were not affected in these mice.

To further confirm our above observations, we generated the conditional Rosa26^*DS‐lncRNA*^ knock‐in mice (DS‐KI mice), in which the endogenous *DS‐lncRNA* gene is transcriptionally activated under the control of the CAG promoter in the presence of Cre recombinase (Figure S12, Supporting Information). Microinjection of AAV5‐Cre into the ipsilateral L3/4 DRGs of DS‐KI mice 28 days before CCI completely rescued the CCI‐induced downregulation of *DS‐lncRNA*, *SIT1*, and *SIT2* in injured DRG on day 14 post‐CCI (Figure 4j) and abolished CCI‐induced mechanical allodynia, heat hyperalgesia, and cold allodynia on the ipsilateral side from days 3 to 14 post‐CCI (Figure 4k–n). Consistently, this microinjection decreased CCI‐induced dorsal horn neuronal/glial hyperactivities as indicated by the abolition of CCI‐induced increases in the phosphorylation of extracellular signal‐regulated kinase 1 and 2 (p‐ERK1/2, a marker for neuronal hyperactivation) and glial fibrillary acidic protein (GFAP, a marker of astrocyte hyperactivation) in the ipsilateral L3/4 dorsal horn (Figure S13, Supporting Information). Although DRG microinjection of AAV5‐Cre significantly increased basal levels of *DS‐lncRNA* and its two transcripts in the ipsilateral L3/4 DRG of sham DS‐KI mice (Figure 4j), basal paw responses to mechanical, heat, and cold stimuli on both sides of these mice were not changed (Figure 4k–n; Figure S11g–i, Supporting Information). Similar results were observed in the DS‐KI mice crossed with sensory neuron‐specific Advillin^Cre/+^ mice (conditional *DS‐lncRNA* overexpression (OE) mice) after SNL (Figure S14a–h, Supporting Information). Both virus‐microinjected DS‐KI mice and conditional *DS‐lncRNA* OE mice exhibited normal locomotor activity (Table S1, Supporting Information). Collectively, these findings indicate that DRG *DS‐lncRNA* downregulation is required for the development and maintenance of neuropathic pain.

### 2.5 Mimicking CCI‐Induced DRG *DS‐lncRNA* Downregulation Leads to Nociceptive Hypersensitivity

**Figure 5:**
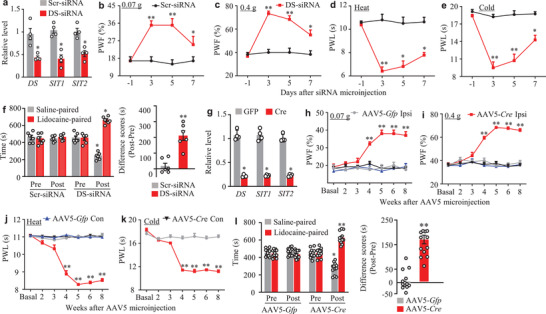
DRG knockdown of *DS‐lncRNA* produced neuropathic pain‐like symptoms. a) Levels of *DS‐lncRNA* (*DS*), *SIT1*, and *SIT2* in the ipsilateral L3/4 DRGs on day 7 after microinjection with *DS‐lncRNA* siRNA (DS‐siRNA) or control scrambled siRNA (Scr‐siRNA) into unilateral L3/4 DRGs. n = 8 mice/group. *P < 0.05 versus the corresponding scrambled siRNA group by two‐tailed unpaired Student's *t*‐test. b–e) Effect of pre‐microinjection of *DS‐lncRNA* siRNA (DS‐siRNA) or control scrambled siRNA (Scr‐siRNA) into unilateral L3/4 DRGs on the paw withdrawal frequency (PWF) to 0.07 g (b) and 0.4 g (c) von Frey filaments and on paw withdrawal latencies (PWL) to heat (d) and cold (e) stimuli on the ipsilateral side at the different days after siRNA microinjection. n = 10 mice/group. *P < 0.05, **P < 0.01 versus the scrambled siRNA‐treated mice at the corresponding time points by two‐way ANOVA with repeated measures followed by post hoc Tukey test. f) Effect of pre‐microinjection of *DS‐lncRNA* siRNA (DS‐siRNA) or control scrambled siRNA (Scr‐siRNA) into unilateral L3/4 DRGs on spontaneous ongoing pain as assessed by the CPP paradigm on day 6 after siRNA microinjection. Pre: preconditioning. Post: post‐conditioning. n = 6 mice/group. *P < 0.05, **P < 0.01 versus the corresponding preconditioning (left) by two‐way ANOVA with repeated measures followed by post hoc Tukey test or versus the scrambled siRNA‐treated group (right) by two‐tailed, independent Student's *t*‐test. g) Levels of *DS‐lncRNA* (*DS*), *SIT1*, and *SIT2* in the ipsilateral L3/4 DRGs 8 weeks after microinjection of AAV5‐Cre (Cre) or AAV‐Gfp (GFP) into unilateral L3/4 DRGs of conditional DS‐lncRNA^fl/fl^ mice. n = 8 mice/group. *P < 0.05 versus the corresponding AAV5‐Gfp group by two‐tailed unpaired Student's *t*‐test. h–k) Effect of microinjection of AAV5‐Cre or AAV5‐Gfp into unilateral L3/4 DRGs of DS‐lncRNA^fl/fl^ mice on the paw withdrawal frequency (PWF) to 0.07 g (h) and 0.4 g (i) von Frey filaments and on paw withdrawal latencies (PWL) to heat (j) and cold (k) stimuli on the ipsilateral (Ipsi) and contralateral (Con) sides at the different weeks after viral microinjection. n = 12 mice/group. **P < 0.01 versus the control AAV5‐Gfp group on the ipsilateral side at the corresponding time points by two‐way ANOVA followed by Tukey post hoc test. l) Effect of microinjection of AAV5‐Cre or AAV5‐Gfp into unilateral L3/4 DRGs of DS‐lncRNA^fl/fl^ mice on spontaneous ongoing pain as assessed by the CPP paradigm 7 weeks after viral microinjection. Pre: preconditioning. Post: post‐conditioning. n = 12 mice/group. *P < 0.05, **P < 0.01 versus the corresponding preconditioning (left) by two‐way ANOVA with repeated measures followed by post hoc Tukey test or versus the AAV‐Gfp group (right) by two‐tailed, independent Student's *t*‐test.

We further examined whether DRG *DS‐lncRNA* downregulation is sufficient for neuropathic pain induction. To this end, *DS‐lncRNA* siRNA that significantly knocked down *DS‐lncRNA* and its two transcripts in *in vitro* and *in vivo* DRG neurons (**Figure** **5**a; Figure S15a, Supporting Information) was microinjected into unilateral L3/4 DRGs of naïve adult mice. Scrambled siRNA was used as a control. The mice microinjected with *DS‐lncRNA* siRNA, but not scrambled siRNA, displayed dramatic increases in paw withdrawal frequencies to 0.07 g and 0.4 g von Frey filaments and decreases in paw withdrawal latencies to heat and cold stimuli on the ipsilateral side on days 3, 5, and 7 after microinjection (Figure 5b–e). In addition, DRG microinjection of *DS‐lncRNA* siRNA (but not scrambled siRNA) led to stimulation‐independent spontaneous ongoing pain evidenced by a robust preference for (that is, spending more time in) the lidocaine‐paired chamber (Figure 5f). Given that siRNA may have potential off‐target effects, we further generated DS‐lncRNA^fl/fl^ mice (Figure S16, Supporting Information) and observed the impact of DRG *DS‐lncRNA* knockdown on nociceptive thresholds through microinjection of AAV5‐Cre into unilateral L3/4 DRGs of these mice (Figure 5g). Like the DS‐lncRNA siRNA‐treated mice, the DS‐lncRNA^fl/fl^ mice microinjected with AAV5‐Cre, but not AAV5‐Gfp, exhibited both evoked mechanical, heat, and cold sensitivities and spontaneous ongoing pain at weeks 4, 5, 6, and 8 after microinjection on the ipsilateral side (Figure 5h–l). Neuronal and astrocyte hyperactivities indicated by robust increases in the levels of p‐ERK1/2 and GFAP, respectively, were also observed in the ipsilateral L3/4 dorsal horn of these mice 8 weeks after AAV5‐Cre microinjection (Figure S17, Supporting Information). As expected, no changes in locomotor function (Table S1, Supporting Information) and basal paw response on the contralateral side were found in either the siRNA‐microinjected wild type mice or the virus‐microinjected DS‐lncRNA^fl/fl^ mice (Figure 5h–l; Figure S15b–d, Supporting Information). It is very likely that, in the absence of nerve injury, DRG *DS‐lncRNA* downregulation leads to neuropathic pain‐like symptoms.

### 2.6 *DS‐lncRNA* Negatively Regulates Expression of *Ehmt2* mRNA/G9a in Injured DRG After CCI

**Figure 6:**
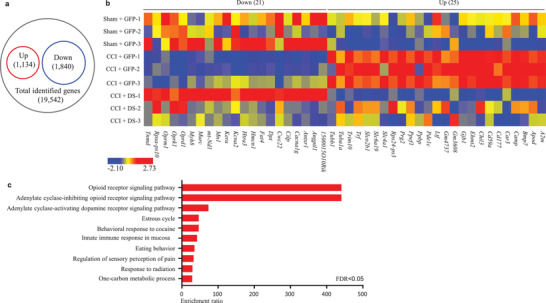
Effect of rescuing nerve injury‐induced *DS‐lncRNA* downregulation on CCI‐induced changes in gene expression in injured DRG using a high‐throughput RNA sequencing assay. Up: upregulated genes. Down: down‐regulated genes. a) The analysis of gene expression profiles in the ipsilateral L3/4 DRG on day 7 after CCI as compared to sham group. Differentially expressed genes were filtered to *P* < 0.05 and log_2_fold‐change. b) Heatmaps of 46 differentially expressed genes (including *Ehmt2*, *Kcna2*, *Oprm1*, *Oprk1*, and *Oprd1* mRNAs), which were reversed by *DS‐lncRNA* overexpression, in the ipsilateral L3/4 DRG on day 7 after CCI or sham surgery in the mice pre‐microinjected with HSV‐DS‐lncRNA (DS) or HSV‐Gfp (GFP) 2 days before surgery. n = 4 mice/group. The scaled heat maps were created using ZA‐score values obtained from RNA sequencing. High expression is shown by the red color spectrum, and low expression is shown in blue. c) Analysis of the Gene Ontology database showed top 10 biological process functions of these 46 differentially expressed genes.

To explore how DRG *DS‐lncRNA* downregulation is involved in neuropathic pain, we carried out high‐throughput RNA sequencing to identify the downstream targets regulated by *DS‐lncRNA* in injured DRG. The unbiased gene expression profiles revealed that about 2,974 genes out of a total of 19, 542 identified genes were significantly changed in the ipsilateral L3/4 DRG from the HSV‐*GFP*‐treated mice on day 7 post‐CCI as compared to those post‐sham surgery (**Figure** **6**a). Approximately 38.13% of these changed genes were upregulated and 61.87% downregulated (Figure 6a). Among these changed genes, 25 upregulated genes and 21 downregulated genes were reversed in the ipsilateral L3/4 DRG from the HSV‐lncRNA‐treated mice on day 7 post‐CCI (Figure 6b, Figure S18, Supporting Information). These affected genes are notably enriched for the opioid receptor signaling pathway (Figure 6c).

**Figure 7:**
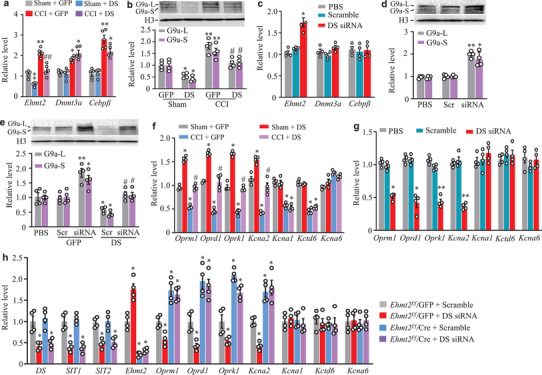
Downregulated *DS‐lncRNA* was required for CCI‐induced increases in *Ehmt2*/G9a expression and consequent G9a‐controlled decreases in opioid receptors and Kcna2 in injured DRG. a,b) Levels of *Ehmt2*, *Dnmt3a* and *Cebpβ* mRNAs (a) and G9a's two protein isoforms (b) in the ipsilateral L3/4 DRG on day 7 after CCI or sham surgery in mice pre‐microinjected with HSV‐DS‐lncRNA (DS) or HSV‐Gfp (GFP) into the unilateral L3/4 DRGs 2 days before surgery. n = 8–10 mice/group. *P < 0.05, **P < 0.01 versus the Sham plus GFP group and #*P* < 0.05, ##*P* < 0.01 versus the CCI plus GFP group by one‐way ANOVA followed by post hoc Tukey test. c,d) Levels of *Ehmt2*, *Dnmt3a*, and *Cebpβ* mRNAs (c) and G9a's two protein isoforms (d) in the ipsilateral L3/4 DRG on day 7 after microinjection of *DS‐lncRNA* siRNA (DS‐siRNA or siRNA), control scrambled siRNA (Scramble or Scr), or PBS into unilateral L3/4 DRGs. n = 8 mice/group. *P < 0.05, **P < 0.01 versus the corresponding PBS group by one‐way ANOVA followed by post hoc Tukey test. e) Levels of G9a's two protein isoforms in cultured DRG neurons treated as indicated. Scr: control scrambled siRNA. siRNA: *DS‐lncRNA* siRNA. GFP: HSV‐Gfp. DS: HSV‐DS‐lncRNA. n = 4 biological repeats/group. *P < 0.05, **P < 0.01 versus the PBS group and #*P* < 0.05 versus the DS plus Scr‐treated group by one‐way ANOVA followed by post hoc Tukey test. f) Levels of *Oprm1*, *Oprd1*, *Oprk1*, *Kcna2*, *Kcna1*, *Kctd6*, and *Kcna6* mRNAs in the ipsilateral L3/4 DRGs on day 7 after CCI or sham surgery in mice pre‐microinjected with HSV‐Gfp (GFP) or HSV‐DS‐lncRNA (DS) into unilateral L3/4 DRGs 2 days before surgery. n = 8 mice/group. *P < 0.05, **P < 0.01 versus the corresponding GFP plus sham group and #*P* < 0.05 versus the GFP plus CCI group by one‐way ANOVA followed by post hoc Tukey test. g) Levels of *Oprm1*, *Oprd1*, *Oprk1*, *Kcna2*, *Kcna1*, *Kctd6*, and *Kcna6* mRNAs in the ipsilateral L3/4 DRG on day 7 after microinjection with PBS, *DS‐lncRNA* siRNA (DS‐siRNA) or control scrambled siRNA (Scramble) into unilateral L3/4 DRGs. n = 8 mice/group. *P < 0.05, **P < 0.01 versus the PBS group by one‐way ANOVA followed by post hoc Tukey test. h) Levels of *DS‐lncRNA*, *SIT1*, and *SIT2* as well as *Ehmt2*, *Oprm1*, *Oprd1*, *Oprk1*, *Kcna2*, *Kcna1*, *Kctd6*, and *Kcna6* mRNAs in cultured DRG neurons from Ehmt2^fl/fl^ mice transduced/transfected as shown. GFP: AAV5‐Gfp. Cre: AAV5‐Cre. Scramble: control scrambled siRNA. DS siRNA: *DS‐lncRNA* siRNA. n = 4 biological repeats/treatment. *P < 0.05 versus the corresponding Ehmt2*^f/f^/GFP* plus Scramble group by one‐way ANOVA followed by post hoc Tukey test.

*Ehmt2/*G9a participates in nerve injury‐induced downregulation of DRG opioid receptor and *Kcna2* genes.^[^ ^9^, ^10^, ^12^, ^13^, ^21^, ^22^, ^40^ ^]^ Our RNA sequencing result showed that the CCI‐induced increase in DRG *Ehmt2* mRNA was blocked in the ipsilateral L3/4 DRG of mice pre‐microinjected with HSV‐lncRNA on day 7 post‐CCI (Figure 6b). RT‐qPCR and Western blot assays further confirmed that DRG microinjection of HSV‐lncRNA blocked CCI‐induced increases in the levels of *Ehmt2* mRNA and G9a protein, but not *Dnmt3a* and *Cebpβ* mRNAs in the ipsilateral L3/4 DRG on day 7 post‐CCI (**Figure** **7**a,b). A similar change in *Ehmt2* mRNA was seen in DS‐KI mice after DRG AAV5‐Cre microinjection on day 14 post‐CCI (Figure S19, Supporting Information). Conversely, DRG *DS‐lncRNA* knockdown through microinjection of its siRNA (but not control scrambled siRNA) robustly increased the amounts of *Ehmt2* mRNA and G9a, but not *Dnmt3a* and *Cebpβ* mRNAs, in the injected DRG on day 5 post‐microinjection (Figure 7c,d). DS‐lncRNA^fl/f/^ mice displayed similar results on day 62 after DRG microinjection of AAV5‐Cre (Figure S20a, Supporting Information). Consistently, the level of G9a was increased or decreased in cultured DRG neurons transfected with *DS‐lncRNA* siRNA or HSV‐lncRNA, respectively (Figure 7e). The HSV‐lncRNA‐induced G9a decrease could be rescued by co‐transfection with *DS‐lncRNA* siRNA (Figure 7e). Additionally, four downstream targets (*Oprm1*, *Oprd1*, *Oprk1*, and *Kcna2* mRNAs) of G9a^[^ ^9^, ^10^, ^12^, ^13^, ^21^, ^40^ ^]^ were correspondingly rescued by *DS‐lncRNA* overexpression in injured DRG on day 7 post‐CCI (Figure 7f) or AAV5‐Cre microinjection‐induced transcriptional activation of the *DS‐lncRNA* gene in injured DRG of DS‐KI mice on day 14 post‐CCI (Figure S19, Supporting Information). As expected, the expression of these four targeted genes was reduced by *DS‐lncRNA* knockdown in injected DRG on day 7 after siRNA microinjection (Figure 7g) or on day 62 after DRG microinjection of AAV5‐Cre in scrambled siRNA‐treated DS‐lncRNA^fl/f/^ mice (Figure S20, Supporting Information). G9a‐independent genes (such as *Kcna1*, *Kctd6*, and *Kcna6*) were not affected (Figure 7f,g; Figures S19, S20, Supporting Information). Collectively, *DS‐lncRNA* negatively regulates *Ehmt2* mRNA/G9a expression and participates in G9a‐controlled downstream signaling in injured DRG after peripheral nerve injury.

To rule out the possibility that *DS‐lncRNA* directly affects the expression of *Ehmt2* mRNA/G9a downstream targeted genes in the DRG neurons, we evaluated the impact of DRG *DS‐lncRNA* downregulation on the expression of *Oprm1*, *Oprd1*, *Oprk1*, and *Kcna2* mRNAs in the presence of DRG G9a knockdown or pharmacological inhibition. The established nociceptive hypersensitivities in responses to mechanical, heat, and cold stimuli (Figure S21a–h, Supporting Information) and downregulation of *Oprm1*, *Oprd1*, *Oprk1*, and *Kcna2* mRNAs in injected DRG (Figure S20b, Supporting Information) from the AAV5‐Cre‐microinjected DS‐lncRNA^fl/f/^ mice were blocked after DRG microinjection of *Ehmt2* siRNA (but not scrambled siRNA) or intraperitoneal injection of BIX01294 (a G9a inhibitor^[^ ^22^ ^]^) once daily for 5 days starting 56 days post‐virus microinjection. Furthermore, a *DS‐lncRNA* siRNA‐induced increase in the level of *Ehmt2* mRNA and corresponding decrease in the amounts of *Oprm1*, *Oprd1*, *Oprk1*, and *Kcna2* mRNAs was not seen in the AAV5‐Cre (compared to AAV5‐GFP)‐transduced cultured DRG neurons from Ehmt2^fl/fl^ mice (Figure 7h). It appears that *Ehmt2* mRNA/G9a is required for DS‐lncRNA to regulate opioid receptor and *Kcna2* gene expression in the DRG neurons.

### 2.7 *DS‐lncRNA* Downregulation Enhances RALY Binding to RNP II and Promotes RALY/RNP II‐Triggered *Ehmt2* Expression in Injured DRG After CCI

**Figure 8:**
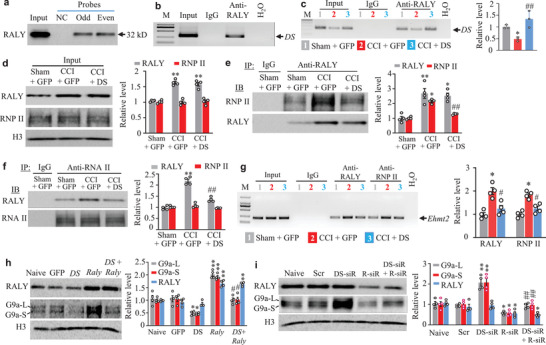
Downregulated *DS‐lncRNA* enhanced RALY binding to the *Ehmt2* gene promoter and promoted RALY‐triggered Ehmt2*/*G9a expression in injured DRG after CCI. a) RALY pulled down by *DS‐lncRNA* probes in cultured DRG neurons. A total of 16 different biotinylated antisense DNA probes that were complementary to the sequence of full‐length *DS‐lncRNA* were designed and numbered. Eight odd‐numbered probes (Odd) and 8 even‐numbered probes (Even) were pooled together, respectively, for hybridization of cell lysates. Input: extracted protein. NC: negative control probes. n = 3 biological repeats. b) *DS‐lncRNA* fragment (DS) immunoprecipitated by rabbit anti‐RALY antibody in the cultured DRG neurons. M: marker. Input: total purified RNA. IgG: purified rabbit IgG. n = 3 biological repeats/treatment. c) *DS‐lncRNA* fragment (DS) immunoprecipitated by rabbit anti‐RALY antibody in the ipsilateral L3/4 DRGs on day 21 after CCI or sham surgery in mice post‐microinjected with HSV‐DS‐lncRNA (DS) or HSV‐Gfp (GFP) starting at day 14 post‐surgery. Input: total purified fragments. M: marker. IgG: purified rabbit IgG. n = 18 mice/group. *P < 0.05 versus the sham plus GFP group and ##*P* < 0.01 versus the CCI plus GFP group by one‐way ANOVA followed by post hoc Tukey test. d–f) RNA polymerase II (RNP II) co‐immunoprecipitated by rabbit anti‐RALY antibody (e) and RALY co‐immunoprecipitated by rabbit anti‐RNP II antibody (f) in the ipsilateral L3/4 DRGs on day 7 after CCI or sham surgery in mice pre‐microinjected with HSV‐DS‐lncRNA (DS) or HSV‐Gfp (GFP) into unilateral L3/4 DRGs 2 days before surgery. Input (d): the purified protein after co‐immunoprecipitation (IP). IB: immunoblotting. IgG: purified rabbit IgG. n = 12 mice/group. *P < 0.05, **P < 0.01 versus the corresponding Sham plus GFP group and ##*P* < 0.01 versus the corresponding GFP plus CCI group by one‐way ANOVA followed by post hoc Tukey test. g) *Ehmt2* promoter fragment immunoprecipitated by rabbit anti‐RALY or rabbit anti‐RNA polymerase II (Anti‐RNP II) in the ipsilateral L3/4 DRGs on day 7 after CCI or sham surgery in mice pre‐microinjected with HSV‐DS‐lncRNA (DS) or HSV‐Gfp (GFP) into unilateral L3/4 DRGs 2 days before surgery. M: marker. Input: total purified RNA. IgG: purified rabbit IgG. n = 12 mice/group. *P < 0.05 versus the corresponding sham plus GFP group and #*P* < 0.05 versus the corresponding CCI plus GFP group by one‐way ANOVA followed by post hoc Tukey test. h) Levels of RALY and the G9a protein's two isoforms in the ipsilateral L3/4 DRG on day 7 after microinjection with HSV‐DS‐lncRNA (DS) or HSV‐Gfp (GFP) into unilateral L3/4 DRGs in mice with or without pre‐microinjection with AAV5‐Raly (Raly) 5 weeks before HSV microinjection. n = 8 mice/group. *P < 0.05, **P < 0.01 versus the corresponding naive group and #*P* < 0.05 versus the corresponding Raly group by one‐way ANOVA followed by post hoc Tukey test. i) Levels of RALY and G9a's two protein isoforms in the ipsilateral L3/4 DRG on day 5 after microinjection with Raly siRNA (R‐siR), *DS‐lncRNA* siRNA (DS‐siR), or control scrambled siRNA (Scr) into unilateral L3/4 DRGs. n = 8 mice/group. *P < 0.05, **P < 0.01 versus the corresponding naive group and ##*P* < 0.01 versus the corresponding R‐siR group by one‐way ANOVA followed by post hoc Tukey test.

Finally, we examined how the *Ehmt2* gene was affected by *DS‐lncRNA* in injured DRG under neuropathic pain conditions. RNA‐binding proteins facilitate RNA transcription.^[^ ^42^ ^]^ To search for *DS‐lncRNA* binding proteins, we designed antisense DNA oligonucleotides (probes) to capture *DS‐lncRNA*, and employed mass spectrometry to identify *DS‐lncRNA*‐binding proteins using ChIRP‐MS assay.^[^ ^43^, ^44^ ^]^ We found that *DS‐lncRNA* interacted with 24 proteins in cultured DRG neurons (Table S3, Supporting Information). Among these interacting proteins, RALY (a heterogeneous ribonucleoprotein^[^ ^35^ ^]^) appeared to be the most likely binding partner (Table S3, Supporting Information). Like *DS‐lncRNA*, RALY is located in DRG neuronal nuclei and highly enriched in the nuclear insoluble fraction (Figure S22a–c, Supporting Information). RALY was pulled down by specific *DS‐lncRNA* probes, but not by a negative control probe, in cultured DRG neurons (**Figure** **8**a). Moreover, a *DS‐lncRNA* fragment immunoprecipitated by anti‐RALY antibody (but not normal purified IgG) was detectable in cultured DRG neurons (Figure 8b) and in *in vivo* sham DRG (Figure 8c). Their binding activity in injured DRG post‐CCI was significantly reduced by 49% compared to that post‐sham surgery on day 21 (Figure 8c). This reduction was attributed to the CCI‐induced downregulation of DRG *DS‐lncRNA*, as rescuing this downregulation through DRG microinjection of HSV‐DS‐lncRNA reversed this reduction (Figure 8c). These *in vitro* and *in vivo* data indicate the binding ability of *DS‐lncRNA* to RALY in DRG.

Unlike *DS‐lncRNA*, RALY was upregulated in injured DRG post‐CCI (Figure 2; Figure S22d, Supporting Information). This upregulation resulted in an increase in its binding activity with RNP II, although basal levels of RNP II were not changed, in injured DRG on day 7 post‐CCI as compared to the sham group (Figure 8d–f). Interestingly, this increased activity was blocked by rescuing *DS‐lncRNA* expression through microinjection of DRG HSV‐lncRNA in injured DRG (Figure 8d–f). This indicates that *DS‐lncRNA* may competitively inhibit the interaction of RALY with RNP II. Given that RALY acts as a transcriptional cofactor for gene expression,^[^ ^35^ ^]^ CCI‐induced *DS‐lncRNA* downregulation could allow more increased RALY to bind to RNP II and the *Ehmt2* gene promoter, which in turn activates *Ehmt2* gene transcription in injured DRG. Consistent with this conclusion, our ChIP assay revealed that both RALY and RNP II bound to the *Ehmt2* promoter and that CCI produced increases in these binding activities in injured DRG on day 7 post‐CCI (Figure 8g). Rescuing *DS‐lncRNA* expression blocked these increases (Figure 8g). DRG overexpression of *DS‐lncRNA* also blocked an increase in G9a protein caused by DRG RALY overexpression through microinjection of AAV5 expressing full‐length Raly mRNA (AAV5‐Raly) into the DRG (Figure 8h). In addition, DRG knockdown of RALY with its siRNA blocked an increase in DRG G9a protein and nociceptive hypersensitivities caused by DRG microinjection of *DS‐lncRNA* siRNA (Figure 8i; Figure S23a–h, Supporting Information). As expected, either DRG knockdown of RALY alone or overexpression of *DS‐lncRNA* alone decreased basal levels of DRG G9a (Figure 8h,i). Given that *DS‐lncRNA* co‐expressed with *Raly* mRNA, *Ehmt2* mRNA, and downstream target *Kcna2* mRNA in individual large, medium, and small DRG neurons as well as downstream target *Oprm1* mRNA in individual medium and small DRG neurons (Figure S24a–c, Supporting Information), our results indicate that *DS‐lncRNA* downregulation may enhance the binding of RALY to RNP II and *Ehmt2* *promoter*, leading to a RALY/RNP II‐triggered increase in *Ehmt2* mRNA/G9a in injured DRG after peripheral nerve injury.

## 3 Discussion

LncRNAs, functioning as a novel regulatory mechanism of gene expression, have attracted widespread attention for their vital roles in myriad biological processes and human diseases.^[^ ^23^, ^24^ ^]^ Bioinformatic analyses from twelve human tissues identified the largest number of brain‐specific lncRNAs.^[^ ^45^ ^]^ The expression levels of these lncRNAs are often altered during neuronal differentiation and in nervous system‐related diseases,^[^ ^46^, ^47^ ^]^ but how they are causally linked to brain‐specific physiological and pathological functions remains elusive. In this study, we identified a DRG‐specifically enriched lncRNA and reported its downregulation in injured DRG neurons following peripheral nerve injury. This downregulation was required for neuropathic pain induction and maintenance through negative regulation of RALY/RNP II‐triggered *Ehmt2* gene expression in the DRG neurons. *DS‐lncRNA* likely is a key player in the mechanisms of nerve injury‐induced pain hypersensitivity.

**Figure 9:**
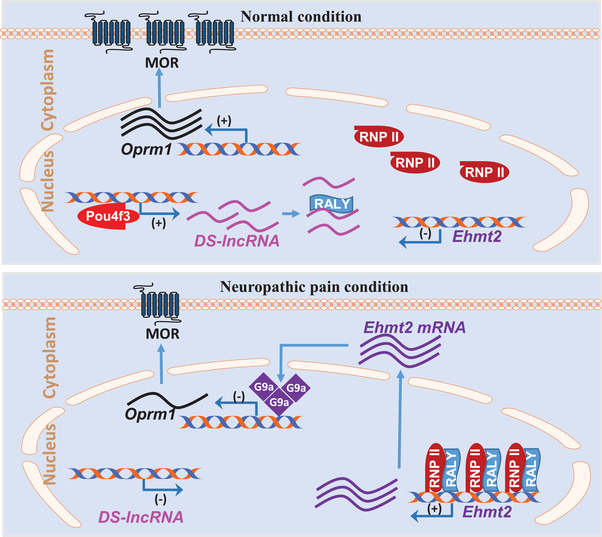
Proposed mechanism by which *DS‐lncRNA* contributes to neuropathic pain. Under normal conditions, the transcription factor Pou4f3 maintains normal expression of *DS‐lncRNA* through its binding to the *DS‐lncRNA* gene promoter and upholding *DS‐lncRNA* basic transcriptional activity in dorsal root ganglion (DRG) neurons. The interaction of *DS‐lncRNA* with the co‐transcription factor RALY causes a loss in the binding of RALY to RNA polymerase II (RNP II) and the *Ehmt2* gene promoter, resulting in *Ehmt2* transcriptional inactivation and low expression of *Ehmt2* mRNA and its coding G9a protein. The latter maintains normal expression of opioid receptors (e.g., Oprm1*/*mu opioid receptor (MOR)) and Kcna2 in DRG neurons. In contrast, peripheral nerve injury downregulates *DS‐lncRNA* due to the silencing of Pou4f3 expression in injured DRG. The downregulated *DS‐lncRNA* promotes the binding of increased RALY to RNP II and the *Ehmt2* gene promoter. The transcriptional activation of the latter increases the expression of *Ehmt2* mRNA and G9a protein. The increased G9a epigenetically represses transcriptional activation of the opioid receptors (e.g., *Oprm1*/MOR) and *Kcna2* genes in injured DRG neurons, resulting in elevations in DRG neuronal excitability and primary afferent neurotransmitter/neuromodulator release.

Expression of *DS‐lncRNA*, like that of other lncRNAs,^[^ ^48^, ^49^ ^]^ can be regulated at a transcriptional level under neuropathic pain conditions. Peripheral nerve injury downregulated *DS‐lncRNA* at least in part through nerve injury‐induced silencing of the transcription factor Pou4f3 and subsequent loss of its binding to the *DS‐lncRNA* gene promoter in injured DRG (**Figure** **9**). Both *DS‐lncRNA* and Pou4f3 mRNA co‐expressed in the nuclei of all types of DRG neurons. A previous report revealed that Pou4f3 was not expressed in non‐peptidergic DRG neurons likely due to the low efficiency of Cre‐mediated recombination because about 10% of Pou4f3 immunolabeled neurons were undetected by this genetic strategy.^[^ ^50^ ^]^ More importantly, we demonstrated that rescuing DRG Pou4f3 not only blocked CCI‐induced nociceptive hypersensitivity but also restored *DS‐lncRNA* expression in injured DRG. DRG knockdown of Pou4f3 reduced DRG *DS‐lncRNA* expression in addition to enhancing responses to nociceptive stimuli in naive mice. These findings strongly support the role of Pou4f3 silence in the nerve injury‐induced DRG *DS‐lncRNA* downregulation. Whether other transcription factors also participate in *DS‐lncRNA* downregulation in injured DRG is unknown. In addition, *DS‐lncRNA* downregulation might also be attributed to a decrease in RNA stability and/or epigenetic modifications. These possibilities cannot be ruled out and will be addressed in future studies.

*DS‐lncRNA* downregulation is required for the RALY/RNP II‐triggered increase in *Ehmt2* mRNA/G9a in injured DRG. −LncRNAs regulate gene expression through their interactions with proteins, DNA, and other RNAs.^[^ ^17^ ^]^ We demonstrated the binding of *DS‐lncRNA* to a heterogeneous ribonucleoprotein RALY in the DRG. We also identified that RALY interacted with RNP II or the *Ehmt2* gene promoter and triggered *Ehmt2* transcriptional activation in the DRG. This transcriptional activation may require nuclear transcription factor Y, as a recent study reported that RALY directly interacted with this transcription factor to influence cholesterogenic gene expression.^[^ ^51^ ^]^ CCI produced *DS‐lncRNA* downregulation, RALY upregulation, and the increases of RALY interaction with RNP II or the *Ehmt2* gene promoter and of RNP II interaction with the *Ehmt2* gene promoter in injured DRG. Given that rescuing DRG *DS‐lncRNA* downregulation not only attenuated these increased interactions but also reversed the CCI‐induced decrease in the binding of RALY to *DS‐lncRNA*, our results suggest that *DS‐lncRNA* may compete with RNP II and the *Ehmt2* gene promoter to bind to RALY (Figure 9). Nerve injury‐induced decreases in the binding of *DS‐lncRNA* to RALY likely result in the greater interaction of the upregulated RALY with RNP II and the *Ehmt2* gene promoter, recruiting more RALY and RNP II to the *Ehmt2* gene promoter, and promoting the G9a expression in injured DRG (Figure 9). This conclusion is further supported by the facts from cultured DRG neurons that the RALY upregulation‐induced increase in G9a expression was blocked by *DS‐lncRNA* overexpression and that the RALY reduction‐induced decrease in G9a expression was attenuated by *DS‐lncRNA* downregulation. *DS‐lncRNA* appears to be a critical regulator in RALY/RNP II‐triggered DRG *Ehmt2* gene expression in DRG neurons.

Downregulated DRG *DS‐lncRNA* contributes to nerve injury‐induced nociceptive hypersensitivity. The present study demonstrated that rescuing *DS‐lncRNA* expression through DRG microinjection of HSV‐DS‐lncRNA in CD1 mice or AAV5‐Cre in DS‐KI mice blocked the increases in *Ehmt2* mRNA and G9a protein and restored the expression of *Oprm1*, *Oprk1*, *Oprd1*, and *Kcna2* mRNAs in injured DRG, thereby abolishing neuropathic pain development and maintenance. Mimicking nerve injury‐induced DRG *DS‐lncRNA* downregulation through DRG microinjection of *DS‐lncRNA* siRNA in CD1 mice or AAV5‐Cre in DS‐lncRNA^fl/fl^ mice increased the expression of *Ehmt2* mRNA and G9a and reduced the expression of *Oprm1*, *Oprk1*, *Oprd1*, and *Kcna2* mRNAs in injected DRG and augmented the animals’ response to noxious stimuli. It is well documented that *Ehmt2* */*G9a is an endogenous initiator of neuropathic pain through its participation in nerve injury‐induced downregulation of *Oprm1*, *Oprk1*, *Oprd1*, and *Kcna2* mRNAs in injured DRG.^[^ ^9^, ^12^, ^13^, ^21^ ^]^ Opioid receptors mediate the inhibitory effect on neurotransmitter/neuromodulator release from primary afferent terminals.^[^ ^10^, ^13^, ^15^, ^21^ ^]^ *Kcna2*‐encoding Kv1.2 protein determines DRG neuronal excitability.^[^ ^12^, ^18^, ^49^, ^52^ ^]^ Given that AAV5‐ or HSV‐mediated knockdown/overexpression strategies predominantly target the DRG neurons,^[^ ^49^, ^53^ ^]^ the anti‐nociception produced by rescuing the downregulated *DS‐lncRNA* in neuropathic pain likely results from the silencing of *Ehmt2*, restoration of opioid receptor and *Kcna2* expression, and reduction in neuronal excitability in injured DRG. The latter may cause a decrease in primary afferent transmitter release, resulting in attenuation of spinal cord central sensitization formation (Figure 9). In line with this conclusion, we found that rescuing *DS‐lncRNA* downregulation in injured DRG abolished the CCI‐induced hyperactivation in dorsal horn neurons and astrocytes. However, other potential mechanisms by which *DS‐lncRNA* participates in neuropathic pain cannot be excluded. Our RNA sequencing assay showed that, in addition to the *Ehmt2* transcript, rescuing DRG *DS‐lncRNA* downregulation also impacted CCI‐induced changes in other DRG transcripts. Whether these transcripts are targeted by *DS‐lncRNA* or mediate its role in neuropathic pain remains to be further investigated. In addition, given that only male mice were used in the present study, whether the mechanisms discussed above are male‐specific is unknown. We will examine these mechanisms in female mice in future studies.

In summary, we demonstrated that in injured DRG, rescuing *DS‐lncRNA* mitigated neuropathic pain without affecting basal/acute pain and locomotor function. Given that *DS‐lncRNA* is uniquely and highly expressed in the DRG but less or no in other tissues, developing drugs that up‐regulate its expression may produce an anti‐nociception with less side‐effects compared to currently available treatments. Thus, *DS‐lncRNA* may be a promising target for neuropathic pain management.

## 4 Experimental Section

### Animals

Male CD1 mice (purchased from Charles River Laboratory, Wilmington, MA), Rosa26^DS‐lncRNA^ KI mice (generated by Biocytogen, Worcester, MA), DS‐lncRNA^fl/fl^ mice (generated by Biocytogen), Advillin^Cre/+^ mice (provided by Dr. Fan Wang at Duke University), and Ehmt2^fl/fl^ mice (provided by Dr. Eric J Nestler at Icahn School of Medicine at Mount Sinai) were used in this study. Rosa26^DS‐lncRNA^ KI mice or DS‐lncRNA^fl/fl^ mice were bred onto a C57B/L6 background for at least 6 generations by crossing the heterozygotes in the facility. Male sensory neuron‐specific Cre line Advillin^Cre/+^ mice were crossed with female Rosa26^DS‐lncRNA^ KI mice to obtain conditional *DS‐lncRNA* OE mice. All animals were kept in a standard 12‐h light/dark cycle, with water and food pellets available *ad libitum*. Male mice weighing 25–30 g were used for the experiments. All procedures used were approved by the Institutional Animal Care and Use Committee at Rutgers New Jersey Medical School and are consistent with the ethical guidelines of the US National Institutes of Health and the International Association for the Study ofPain. All efforts were made to minimize animal suffering and to reduce the number of animals used. All of the experimenters were blind to the treatment condition.

### Animal Models

For neuropathic pain models, CCI of the sciatic nerve and L4 SNL were carried out as described previously.^[^ ^10^, ^18^, ^49^, ^54^ ^]^ Briefly, mice were placed under anesthesia with isoflurane. For the CCI model, the unilateral sciatic nerve was exposed and loosely ligated with 7‐0 silk thread at four sites at intervals of about 1mm, proximal to the trifurcation of the sciatic nerve. The SNL model was performed by ligating the unilateral fourth lumbar spinal nerve with 7‐0 silk suture and transecting it distal to this site. For sciatic nerve axotomy, the unilateral exposed sciatic nerve was cut at a point approximately 1 cm distal to its spinal nerve root exit point as described previously.^[^ ^52^, ^55^ ^]^ Proximal and distal stumps were separated to ensure full transection. Sham animals received an identical surgery but without the ligation or transection of the respective nerve.

### DRG Microinjection

DRG microinjection was carried out as described previously with minor modification.^[^ ^10^, ^18^, ^49^, ^54^ ^]^ Briefly, a midline incision was made in the lower lumbar back region after the mouse was anesthetized with isoflurane. The L4 and/or L3 articular processes were exposed and then removed. Viral solution (1 µL/DRG, 4 × 10^12^) or siRNA solution (1 µL/DRG, 40 x 10^–6^ m) was injected into unilateral exposed L3/4DRGs with the use of a glass micropipette connected to a Hamilton syringe. The pipette was removed 10 min after injection. The surgical field was irrigated with sterile saline and the skin incision closed with wound clips. Mice showing signs of paresis or other abnormalities were excluded. Injected DRGs that were stained with hematoxylin/eosin confirmed the integrity of their structure and demonstrated no visible leukocytes.

### Behavioral Tests

All mice were habituated 1 to 2 h every day for 2 to 3 days before basal behavioral testing. The evoked behavioral testing including mechanical, heat, and cold tests was carried out in sequential order at 1 h intervals. Conditional place preference (CPP) testing was performed at the seventh week after viral injection. Locomotor function testing was carried out after all behavioral tests described above were done.

Paw withdrawal thresholds in response to mechanical stimuli were measured with two calibrated von Frey filaments (0.07 and 0.4 g, Stoelting Co., Wood Dale, IL).^[^ ^10^, ^18^, ^49^, ^54^ ^]^ Briefly, mice were placed in a Plexiglas chamber on an elevated mesh screen and allowed to habituate for 30 min. Each von Frey filament was applied to the plantar sides of both hind paws 10 times. A quick withdrawal of the paw was regarded as a positive response. The number of positive responses among 10 applications was recorded as percentage withdrawal frequency [(number of paw withdrawals/10 trials) × 100 = % response frequency].

Paw withdrawal latencies in response to noxious heat stimuli were examined with a Model 336 Analgesia Meter (IITC Inc. Life Science Instruments. Woodland Hills, CA).^[^ ^10^, ^18^, ^49^, ^54^ ^]^ Briefly, mice were placed in a Plexiglas chamber on a glass plate. A beam of light was emitted from a hole in the lightbox and applied to the middle of the plantar surface of each hind paw. The light beam was automatically turned off when the hind paw was quickly lifted. The length of time between the start and the stop of the light beam was defined as the paw withdrawal latency. For each side, five trials at 5‐min intervals were carried out. A cutoff time of 20 s was used to avoid tissue damage to the hind paw.

Paw withdrawal latencies to noxious cold (0 °C) were examined as described.^[^ ^10^, ^18^, ^49^, ^54^ ^]^ Mice were placed in a Plexiglas chamber on the cold aluminum plate, the temperature of which was monitored continuously by a thermometer. The paw withdrawal latency was recorded as the length of time between placement and the first sign of the mouse jumping and/or flinching. Each test was repeated three times at 10‐min intervals on the ipsilateral side. To avoid tissue damage, a cut‐off time of 20 s was used.

CPP test was carried out as described with minor modifications.^[^ ^10^, ^18^, ^49^, ^54^ ^]^ Briefly, an apparatus with two Plexiglas chambers connected with an internal door (Med Associates Inc., St. Albans, VT) was used. One of the chambers has a rough floor and walls with horizontal black and white stripes, whereas the other contained a smooth floor and walls with vertical black and white stripes. Movement of the mice and time spent in each chamber was monitored by photobeam detectors installed along the chamber walls and automatically recorded in MED‐PC IV CPP software. Mice were first preconditioned for 30 min with full access to both chambers to habituate them to the environment. At the end of the preconditioning phase, basal duration spent in each chamber was recorded within 15 min to check whether mice had a preexisting chamber bias. Mice spending more than 80% or less than 20% of the total time in any chamber were excluded from further testing. The conditioning protocol was performed for the following 3 days with the internal door closed. The mice first received an intrathecal injection of saline (5 µL) specifically paired with one conditioning chamber in the morning. Six hours later, lidocaine (0.8% in 5 µL of saline) was given intrathecally paired with the opposite conditioning chamber in the afternoon. Lidocaine at this dosage did not affect motor function. Injection order of saline and lidocaine was switched every day. On the test day, at least 20 h after the conditioning, the mice were placed in one chamber with free access to both chambers. The duration of time that each mouse spent in each chamber was recorded for 15 min. Difference scores were calculated as test time‐preconditioning time spent in the lidocaine chamber.

Locomotor function, including placing, grasping, and righting reflexes, were examined as described.^[^ ^10^, ^18^, ^49^, ^54^ ^]^ 1) Placing reflex: The placed positions of the hind limbs were slightly lower than those of the forelimbs, and the dorsal surfaces of the hind paws were brought into contact with the edge of a table. Whether the hind paws were placed on the table surface reflexively was recorded; 2) Grasping reflex: After the animal was placed on a wire grid, whether the hind paws grasped the wire on contact was recorded; 3) Righting reflex: When the animal was placed on its back on a flat surface, whether it immediately assumed the normal upright position was recorded. Each trial was repeated 5 times at 5‐min interval and the scores for each reflex were recorded based on counts of each normal reflex.

### Cell Culture and Transfection

HEK‐293T cell cultures and DRG neuronal cultures were prepared according to previously described methods.^[^ ^10^, ^18^, ^49^, ^54^ ^]^ Briefly, HEK‐293T cells were cultured in Dulbecco's modified Eagle's medium/high glucose medium (Gibco/Thermo Fisher Scientific) containing 10% fetal bovine serum (FBS) and 1% antibiotics. For primary DRG neuronal cultures, after 4‐week‐old CD1 mice were euthanized with isoflurane, all DRGs were collected in cold Neurobasal Medium (Gibco/ThermoFisher Scientific) containing 10% FBS (JR Scientific, Woodland, CA), 100 units mL^−1^ Penicillin and 100 µg mL^−1^ Streptomycin (Quality Biological, Gaithersburg, MD). The DRGs were then treated with enzyme solution (5 mg mL^−1^ dispase, 1 mg mL^−1^ collagenase type I in Hanks’ balanced salt solution (HBSS) without Ca^2+^ and Mg^2+^ (Gibco/ThermoFisher Scientific). After trituration and centrifugation, dissociated cells were resuspended in a mixed Neurobasal Medium and plated in a six‐well plate coated with 50 µg mL^−1^ poly‐D‐lysine (Sigma, St. Louis, MO). The cells were incubated at 95% O_2_, 5% CO_2_, and 37 ℃. On the second day, 2–10 µL of virus (titer ≥1 × 10^12^/µL) or siRNA (100 x 10^–9^ m; transfected with Lipofectamine 2000) was added to every 2 mL well. Cells were collected 3 days later.

### Reverse Transcription (RT)‐PCR Assay

The unilateral L3/4 DRGs from two adult CCI, axotomy, or sham surgery mice, or the unilateral L4 DRGs from four SNL or sham mice, or the cultured DRGs neurons from one well of a 6‐well plate were collected rapidly and pooled together to achieve enough RNA. Total RNA was extracted by the RNeasy Mini Kit (Qiagen, Valencia, CA) and treated with excess DNase I (New England Biolabs, Ipswich, MA). Highly purified, DNase‐treated RNA samples from human DRG were purchased from Clontech Laboratories, Inc. (Mountain View, CA). RNA concentration was measured using the NanoDrop 2000 Spectrophotometer (Thermo Scientific, Wilmington, DE) and Qubit Fluorometric Quantitation (Invitrogen, Carlsbad, CA). Ratios of A260/280nm were between 1.97 and 2.08. RNA (0.5 µg) was reverse‐transcribed into single‐stranded cDNA using the Omniscript RT Kit (Qiagen) with specific RT‐primers, and the cDNA template (1 µL) was amplified by real‐time PCR using the primers listed in the Table S2 (Supporting Information). Each sample was run in triplicate in a 20 µL reaction using SsoAdvanced Universal SYBR Green Supermix (Bio‐Rad Laboratories, Hercules, CA). Reactions were carried out in a BIO‐RAD CFX96 real‐time PCR system. Ratios of RNA levels at different time points post‐surgery to RNA level 1 day before surgery or of RNA levels in other treated groups to RNA level in the control group were calculated using the ΔCt method (2^−ΔΔCt^). All data were normalized to *Tuba1α*, as it was demonstrated to remain stable even after peripheral nerve injury.^[^ ^10^, ^18^, ^49^, ^54^ ^]^

### Single‐Cell RT‐PCR

Single‐cell RT‐PCR was carried out as described.^[^ ^10^, ^18^, ^49^, ^54^ ^]^ In brief, the freshly cultured mouse DRG neurons were prepared. Four hours after plating, under an inverted microscope fitted with a micromanipulator and microinjector, single living large (> 35 µm), medium (25–35 µm), and small (< 25 µm) DRG neurons were collected in a PCR tube with 9–10 µL of cell lysis buffer (Signosis, Sunnyvale, CA). After centrifugation, the supernatants were collected and divided into PCR tubes for different genes. The remaining RT‐PCR procedure was performed according to the manufacturer's instructions with the Single‐Cell RT‐PCR Assay Kit (Signosis). All nest‐PCR primers used are listed in Table S2 (Supporting Information).

### Rapid Amplification of cDNA ends (RACE)

RNA fragments amplified from the mouse DRG was extended first by using RT‐PCR with strand‐specific primers and then by using a RACE Kit (2nd Generation, Roche Diagnostics, Indianapolis, IN). The 5′ RACE was used for amplification of the 5′‐end of cDNA according to the manufacturer's instructions. The 3′ RACE analysis was performed by ligating an adapter to the 3‐hydroxyl group of the RNA, followed by gene‐ and adapter‐specific amplification. All primers are listed in Table S2 (Supporting Information). PCR products from RT‐PCR, 5′ RACE, and 3′ RACE were extracted, purified, and verified by automatic DNA sequencing. All sequences were analyzed and the full‐length *SIT1* and *SIT2* were determined.

### Subcellular Fractionation

Subcellular fractionation was carried out as described before^[^ ^10^, ^18^, ^35^, ^40^ ^]^ with minor modification. Briefly, after being rinsed with 1× PBS/1 x 10^–3^ m EDTA and centrifuged, the cultured DRG neurons were collected. The pellets were resuspended in ice‐cold lysis buffer (10 x 10^–3^ m Tris‐HCl (pH 7.5), 0.05% NP40, and 150 x 10^–3^ m NaCl) for 5 min. The lysate was then layered on top of 2.5 volumes of a chilled sucrose cushion (24% sucrose in lysis buffer) and centrifuged at 14, 000 rpm for 10 min at 4 °C. The supernatant (cytoplasmic fraction) was collected and treated with proteinase K for 1 h at 37 °C. RNA was extracted with phenol/chloroform and precipitated with ethanol. The nuclear pellets were gently rinsed with ice‐cold 1×PBS/1 x 10^–3^ m EDTA, then re‐suspended in a prechilled glycerol buffer (20 x 10^–3^ m Tris‐HCl (pH 7.9), 75 x 10^–3^ m NaCl, 0.5 x 10^–3^ m EDTA, 0.85 x 10^–3^ m DTT, 0.125 x 10^–3^ m PMSF, 50% glycerol) by gentle flicking of the tube. After an equal volume of cold nuclear lysis buffer (10 x 10^–3^ m HEPES (pH 7.6), 1 x 10^–3^ m DTT, 7.5 x 10^–3^ m MgCl_2_, 0.2 x 10^–3^ m EDTA, 0.3 m NaCl, 1 m UREA, 1%NP‐40) was added, the tube was gently mixed via vortex, incubated for 2 min on ice, and then centrifuged at 14 , ​000 rpm for 2 min at 4 °C. RNA was extracted with phenol/chloroform and precipitated with ethanol. The supernatant (soluble nuclear fraction) was treated with proteinase K for 1 h at 37 °C. The chromatin pellet was gently rinsed with cold 1 × PBS/1 x 10^–3^ m EDTA and then dissolved in TRIzol (Invitrogen). Chromatin‐associated RNA was purified according to the TRIzol protocol, but an additional phenol/chloroform extraction step was performed prior to precipitation. All RNA fractions were resuspended in TE (pH 7), quantified, and tested for DNA contamination by RT‐PCR lacking reverse transcriptase.

### Plasmid Constructs and Virus Production

Full‐length *Pou4f3* or *Raly* cDNA were respectively amplified from mouse DRG RNA by using the SuperScript III One‐Step RT‐qPCR System with the Platinum Taq High Fidelity Kit (Invitrogen/Thermo‐Fisher Scientific) and forward primers with BspEI and reverse primers with NotI restriction sites (Table S2, Supporting Information). After double enzyme digestion, the PCR products were ligated into the BspEI/NotI sites of the proviral plasmids (University of North Carolina, Chapel Hill) to replace enhanced GFP (EGFP) and the S‐D sequence. The resulting vectors expressed the genes under the control of the cytomegalovirus promoter. AAV5 packaging of viral particles carrying the cDNA was carried out using the AAVpro Purification Kit (Takara, Mountain View, CA). The virus titer was evaluated using the AAVpro Titration Kit (Takara). AAV5‐Cre was purchased from UNC Vector Core. HSV‐Gfp and HSV‐lncRNA were constructed/provided by the McGovern Institute for Brain Research at MIT. *DS‐lncRNA* siRNA (Table S2, Supporting Information) and its negative control siRNA and *Ehmt2* siRNA (s168026) were purchased from Thermo Fisher Scientific, Inc. (Waltham, MA).

### Northern Blotting

To prepare complementary RNA (cRNA) probes of mouse *DS‐lncRNA*, a PCR product with a 310 bp fragment was amplified using mouse cDNA with a pair of primers including the T7 promoter at the 3′ end (Table S2, Supporting Information) and identified using DNA sequencing. After PCR purification, a riboprobe was generated through in vitro transcription and labeled with digoxigenin‐dUTP according to the manufacturer's instructions (Roche Diagnostics, Indianapolis, IN) at 37 °C for 2 h. The probes were purified using Micro Bio‐Spin 30 Chromatography Column (Bio‐Rad).

Northern blot analysis was performed as described previously.^[^ ^49^, ^56^ ^]^ Briefly, the extracted RNA (10 µg) was separated on a 1.5% agarose/formaldehyde gel, transferred to a BrightStar‐plus positively charged nylon membrane, and cross‐linked using UV light. After pre‐hybridization, the membrane was hybridized overnight at 68 °C with a digoxigenin‐UTP‐labeled cRNA probe for *DS‐lncRNA*. The membrane was washed in low salt buffer at room temperature for 2 × 5 min, high salt buffer at 68 °C for 2 × 5 min, and SSC at 68 °C for 1 × 2 min. After being blocked, the membrane was incubated with alkaline phosphatase‐conjugated sheep anti‐digoxigenin (1:500, Roche) for 1 h at room temperature, and washed for 2 × 5 min, incubated by luminol/enhancer reagent (Clarity Western ECL Substrate, Bio‐Rad) and exposed by the ChemiDoc XRS System with Image Lab software (Bio‐Rad).

### In Situ Hybridization Histochemistry (ISHH)

The ISHH was carried out as described previously with minor modification.^[^ ^40^, ^49^ ^]^ For single‐labeling, two sets of 20‐µm sections from each DRG were collected by grouping every third section. A *DS‐lncRNA* cRNA antisense probe (0.31‐kb fragment) and sense cRNA probe (0.31‐kb fragment) were prepared through in vitro transcription and labeled with digoxigenin‐dUTP according to the manufacturer's instructions (Roche Diagnostics, Indianapolis, IN). After being treated with proteinase K for 15 min, two sets of sections were pre‐hybridized for 1.5 h at 65 °C and hybridized with digoxigenin‐dUTP‐labeled antisense and sense cRNA probes, respectively, at 65 °C overnight. After being blocked, the sections were incubated with alkaline phosphatase‐conjugated sheep anti‐digoxigenin (1:500, Roche) overnight at 4 °C. After being washed in 1× PBST, TNT buffer, and detection buffer, respectively, the fluorescent signals were developed by incubation with Fast‐Red dye. For the double labeling of ISHH, the sections were first hybridized with a digoxigenin‐dUTP–labeled cRNA probe for *DS‐lncRNA* antisense, then co‐incubated with alkaline phosphatase‐conjugated sheep anti‐digoxigenin (1:500, Roche) and chicken ant‐*β*‐tubulin‐III (1:200, EMD Millipore), rabbit anti‐glutamine synthetase (1:500, Sigma‐Aldrich), rabbit anti‐NF200 (1:100, Sigma), biotinylated IB4 (1:200, Sigma) or mouse anti‐CGRP (1:50, Abcam), respectively, overnight at 4 °C. The fluorescent signals were developed with Fast Red and appropriate fluorescence‐conjugated secondary antibodies.

### In Vitro Protein Translation

The full‐length *DS‐lncRNA* DNA fragment containing the T7 promoter was obtained by RT‐PCR (Table S2, Supporting Information) and was *in vitro* transcribed and translated by the Transcend Non‐Radioactive Translation Detection System as described in the manufacturer's manual (Promega, Madison, WI). In this system, biotinylated lysine residues were incorporated into nascent proteins during translation, allowing for *in vitro* non‐radioactively‐labeled protein synthesis. Proteins were detected by incubation with streptavidin‐horseradish peroxidase and visualized using Western blotting.^[^ ^57^ ^]^ Luciferase and Creb1 were used as coding gene controls, whereas the lncRNA H19 was used as a noncoding gene control.

### Bioinformatic Prediction of Transcription Factors

The University of Santa Cruz (UCSC) genomic database (https://genome.ucsc.edu) was used to acquire the 2000‐bp promoter sequence of the *DS‐lncRNA* gene. The JASPAR database (http://jaspar.genereg.net/) was used to predictively analyze whether there were binding motifs for Pou4f3 in the promoter region of the *DS‐lncRNA* gene. The relative profile score threshold was set as 80%. Through analyzing the predicted score from the potential Pou4f3‐binding regions, the region with the highest score was used to clone and to investigate the binding between POU4F3 and *DS‐lncRNA*.

### Co‐Immunoprecipitation (Co‐IP)

Co‐IP was carried out according to the methods described previously^[^ ^58^, ^59^, ^60^ ^]^ with minor modifications. Briefly, unilateral L3/4 DRGs from two CCI or sham mice were collected rapidly and pooled together. The tissues were homogenized in chilled lysis buffer. Total protein was incubated in rabbit anti‐RALY‐antibody (Abcam) or rabbit anti‐RNP II‐antibody (Abcam) at 4 °C for 1 h. The purified rabbit or mouse IgG was used as a control. The protein A/G beads were then added for rotation at 4 °C overnight. After being centrifuged at 1,000 g for 5 min at 4 °C, the pellets were collected, washed 4 times with 1 mL IP buffer, and heated in 40 µL electrophoresis buffer at 99 °C for 10 min. Proteins were separated by SDS‐PAGE and detected by RALY or RNP II antibody as described below.

### Luciferase Reporter Assay

A 1,002‐bp fragment from the *DS‐lncRNA* promoter region (including the Pou4f3‐binding motif) was amplified by PCR from genomic DNA using primers (Table S2, Supporting Information) to construct the *DS‐lncRNA* gene reporter plasmid as described previously.^[^ ^49^ ^]^ The PCR products were cloned into the KpnI and NheI restriction sites of pGL3‐Basic vector (Promega, Madison, WI). The accuracy of recombinant clones was verified by DNA sequencing. HEK‐293T cells were plated on a 12‐well plate and cultured at 37 °C in a humidified incubator with 5% CO_2_. One day after culture, the cells in each well were co‐transfected with 300 ng of a plasmid expressing full‐length Pou4f3, 300 ng of pGL3‐Basic vector with or without the *DS‐lncRNA* promoter sequence, and 10 ng of the pRL‐TK (Promega) using Lipofectamine 2000 (Invitrogen), according to the manufacturer's instructions. The wells were divided into different groups as indicated. Two days after transfection, the cells were collected and lysed in a passive lysis buffer. Approximately 10 µL of supernatant was used to measure the luciferase activity using the Dual‐Luciferase Reporter Assay System (Promega). Transfection experiments were repeated 3 independent times. Relative reporter activity was calculated after normalization of the firefly activity to renilla.

### Comprehensive Identification of RNA Binding Proteins by Mass Spectrometry (ChIRP‐MS)

ChIRP was performed as described previously.^[^ ^10^, ^44^ ^]^ DRGs from 18 mice were collected and pooled together for one ChIRP test. The cultured DRG neurons from these mice were prepared as mentioned above. Three days after culture, DRG neurons were cross‐linked by using 3% formaldehyde for 30 min. The reactions were quenched by adding 0.125m glycine for 5 min. After being rinsed with chilled PBS, cell pellets were dissolved in nuclear lysis buffer. A total of 16 different biotinylated antisense DNA probes that were complementary to the *DS‐lncRNA* sequence were prepared using an online tool (http://www.singlemoleculefish.com) and numbered. The negative control probes that were not complementary to the sequence of *DS‐lncRNA* were used as a control. Eight odd‐numbered probes, 8 even‐numbered probes, and negative control probes were hybridized separately with the cell lysates overnight. The complex of beads/probes/RNA/protein was pulled down using streptavidin magnetic C1 beads (Invitrogen). The collected proteins were solubilized in Laemmli sample buffer and subjected to Bis‐Tris SDS‐PAGE. Mass spectrometry experiments and data analysis were carried out in the Center for Advanced Proteomics Research, Rutgers New Jersey Medical School. Each gel lane was cut into five fractions, and in‐gel trypsin digestion was performed. The resulting peptides were desalted with a C18 spin column and then analyzed by LC‐MS/MS on a Q Exactive Mass Spectrometer. The MS/MS spectra were searched against the Swiss‐Prot Mus Musculus database using a local MASCOT (V.2.4) search engine on the Proteome Discoverer (V1.4) platform. Through the analysis of Mass Spectrometry Proteomics, only those proteins simultaneously pulled down by both odd and even numbered probes, but not by negative control probes, were considered to be positive targeted proteins. As described below, Western blot analysis was carried out to verify the binding of *DS‐lncRNA* to RALY.

### RNA Sequencing

The ipsilateral L3/4 DRGs were harvested on day 7 post‐CCI or sham surgery in mice pre‐microinjected with HSV‐DS‐lncRNA or HSV‐Gfp into the ipsilateral L3/4 DRGs. Eight DRGs from four mice were pooled together to achieve enough RNA. Total RNA (1.2 µg/sample) extracted as described above was subjected to rRNA depletion by Ribo‐Zero rRNA Removal (Human/Mouse/Rat) Kit (Illumina, San Diego, CA, USA). Strand‐specific RNA libraries were prepared using TruSeq Stranded Total RNA Sample Preparation Kit (Illumina) without poly‐A selection. All assays were performed according to the manufacturer's instructions. Sequencing was carried out using the Illumina HiSeq2500 platform High Output Mode, in a 2×100 bp paired‐end configuration, with a total of more than 190 m reads per lane (at least 60 m reads per sample).^[^ ^26^ ^]^

### Chromatin Immunoprecipitation (ChIP) Assay

ChIP was performed using the EZ ChIP Kit (Upstate/EMD Millipore, Darmstadt, Germany) as described previously.^[^ ^12^, ^18^, ^49^ ^]^ In brief, DRG homogenates were crosslinked with 1% formaldehyde for 10 min at room temperature. The reaction was terminated by the addition of 0.25 m glycine. After centrifugation, the collected pellet was lysed by SDS lysis buffer with a protease inhibitor cocktail and sonicated until the DNA was broken into fragments with a mean length of 200 to 1000 bp. After the samples were pre‐cleaned with protein G agarose, they were subjected to immunoprecipitation overnight with 2 µg of rabbit anti‐RNP II (Millipore), rabbit anti‐RALY1 (Abcam), rabbit anti‐Pou4f3 (Thermo Fisher), or purified rabbit IgG overnight at 4 °C. Input (10–20% of the sample for immunoprecipitation) was used as a positive control. The DNA fragments were purified and identified using PCR/Real‐time PCR with the primers listed in Table S2 (Supporting Information).

### Western Blotting

To achieve sufficient protein, four unilateral mouse DRGs were pooled together. The tissues were homogenized and the cultured cells ultrasonicated in chilled lysis buffer (10 x 10^–3^ m Tris, 1 x 10^–3^ m phenylmethylsulfonyl fluoride, 5 x 10^–3^ m MgCl_2_, 5 x 10^–3^ m EGTA, 1 x 10^–3^ m EDTA, 1 x 10^–3^ m DTT, 40 x 10^–6^ m leupeptin, 250 x 10^–3^ m sucrose). After centrifugation at 4 °C for 15 min at 1,000 g, the supernatant was collected for cytosolic proteins and the pellet for nuclear proteins. The content of the proteins in the samples was measured using the Bio‐Rad protein assay (Bio‐Rad) and the samples were then heated at 99 °C for 5 min and loaded onto a 4–15% stacking/7.5% separating SDS‐polyacrylamide gel (Bio‐Rad Laboratories). The proteins were then electrophoretically transferred onto a polyvinylidene difluoride membrane (Bio‐Rad Laboratories). After the membranes were blocked with 3% nonfat milk in Tris‐buffered saline containing 0.1% Tween‐20 for 1 h, they were incubated overnight at 4 °C with the following primary antibodies including mouse anti‐*α*‐tubulin (1:1,000, Santa Cruz), rabbit anti‐G9a (1:1,000, Cell signaling, Danvers, MA), rabbit anti‐ RNP II (1:500, Millipore), rabbit anti‐RALY (1:1,000, Abcam), rabbit anti‐Pou4f3 (1:500, Invitrogen), rabbit anti‐phospho‐ERK1/2 (Thr202/Tyr204, 1:1,000, Cell Signaling), rabbit anti‐ERK1/2 (1:1,000, Cell Signaling), mouse anti‐GFAP (1:1,000, Cell Signaling), rabbit anti‐GAPDH (1:2,000, Santa Cruz, Dallas, TX)—, and rabbit anti‐histone H3 (1:1,000, Cell Signaling). The proteins were detected by horseradish peroxidase‐conjugated anti‐mouse or anti‐rabbit secondary antibody (1:3,000, Jackson ImmunoResearch) and visualized by western peroxide reagent and luminol/enhancer reagent (Clarity Western ECL Substrate, Bio‐Rad) and exposed using the ChemiDoc XRS System with Image Lab software (Bio‐Rad). The intensity of blots was quantified with densitometry using Image Lab software (Bio‐Rad). All cytosolic protein bands were normalized to *α*‐tubulin or GAPDH and all nuclear proteins to histone H3.

### Immunohistochemistry

Mice were anesthetized and perfused with 4% formaldehyde in 0.1m phosphate‐buffered saline (PBS, pH 7.4). DRGs were dissected rapidly, postfixed in the same fixative solution at 4 °C for 4 h, and cryoprotected in 30% sucrose overnight. A series of 20‐µm transverse sections were cut on a cryostat. The sections were blocked with 5% goat serum and 0.3% TritonX‐100 in 0.01 m PBS at room temperature for 1 h and then incubated with rabbit anti‐Pou4f3 (1:800, GeneTex) plus biotinylated IB4 (1:200, Sigma), mouse anti‐CGRP (1:200, Abcam), mouse anti‐NF200 (1:200, Sigma), mouse anti‐glutamine synthetase (1:1,000, Sigma) or chicken *β*‐tubulin III (1:500, EMD Millipore) or with rabbit anti‐RALY (1:2,500, Abcam) plus mouse anti‐glutamine synthetase (1:1,000, Sigma) or chicken *β*‐tubulin III (1:500, EMD Millipore) at 4 °C over‐two nights. The sections were then incubated with species‐appropriate Cy2‐ or Cy3‐conjugated secondary antibody (1:500, Jackson ImmunoResearch), or FITC‐labeled Avidin D (1:200, Sigma) at room temperature for 2 h. Control experiments were performed in parallel. The images were captured with a Leica DMI4000 fluorescence microscope (Leica).

### Statistical Analysis

For *in vitro* experiments, the cells were evenly suspended and then randomly distributed into each well tested. For *in vivo* experiments, the animals were distributed into various treatment groups randomly. The sample sizes were determined based on the pilot studies, previous reports in the field,^[^ ^49^, ^58^, ^60^, ^61^, ^62^ ^]^ and power analyses (power of 0.90 at p < 0.05). All of the results were given as means ± S.E.M. Data distribution was assumed to be normal but this was not formally tested. The data were statistically analyzed using two‐tailed, paired Student's *t*‐test, and a one‐way or two‐way ANOVA. When ANOVA showed a significant difference, a pairwise comparison between means was performed using the post hoc Tukey method (SigmaPlot 12.5, San Jose, CA). Significance was set at *P* < 0.05.

## Conflict of Interest

The authors declare no conflict of interest.

## Author Contributions

Z.P., S.D., and K.W. contributed equally to this work. Y.X.T. conceived the project and supervised all experiments. Z.P., S.D., and K.W. contributed towards the development and execution of the project, each making substantial contributions towards this work, including design, acquisition, analysis, or interpretation of the data presented. Z.P., S.D., K.W., X.F., S.W., and B.H. carried out molecular, biochemical, and morphological experiments. S.D., K.W., X.G., Q.M., and L.H. prepared animal models and conducted behavioral experiments. Z.P. and Y.J.C. carried out the bioinformatic analysis. Z.P., T.L., T.C., and H.L. performed mass spectrometry assay and data analysis. Z.P., S.D., K.W., A.B., H.H., and Y.X.T. analyzed the data. Z.P. and Y.X.T. wrote the draft of the manuscript. T.B. and Y.X.T. edited the final manuscript. All authors read and discussed the manuscript.

## Supporting information

Supporting Information

## Data Availability

Research data are not shared.
