# Supplementary material for: Downregulation of a Dorsal Root Ganglion‐Specifically Enriched Long Noncoding RNA is Required for Neuropathic Pain by Negatively Regulating RALY‐Triggered Ehmt2 Expression
Source: Adv Sci (Weinh). 2021 May 14;8(13):2004515. doi: 10.1002/advs.202004515 (PMC8356248; doi:10.1002/advs.202004515)
Supplement: Supplementary file 1 — Supporting Information [file ADVS-8-2004515-s001.pdf]

© 2021 Wiley-VCH GmbH

ADVANCED SCIENCE Open Access

Supporting Information

for *Adv. Sci.*, DOI: 10.1002/advs.202004515

Downregulation of a Dorsal Root Ganglion-Specifically Enriched Long Noncoding RNA is Required for Neuropathic Pain by Negatively Regulating RALY-Triggered Ehmt2 Expression

*Zhiqiang Pan, Shibin Du, Kun Wang, Xinying Guo, Qingxiang Mao, Xiaozhou Feng, Lina Huang, Shaogen Wu, Bailing Hou, Yun-Juan Chang, Tong Liu, Tong Chen, Hong Li, Thomas Bachmann, Alex Bekker, Huijuan Hu, and Yuan-Xiang Tao**

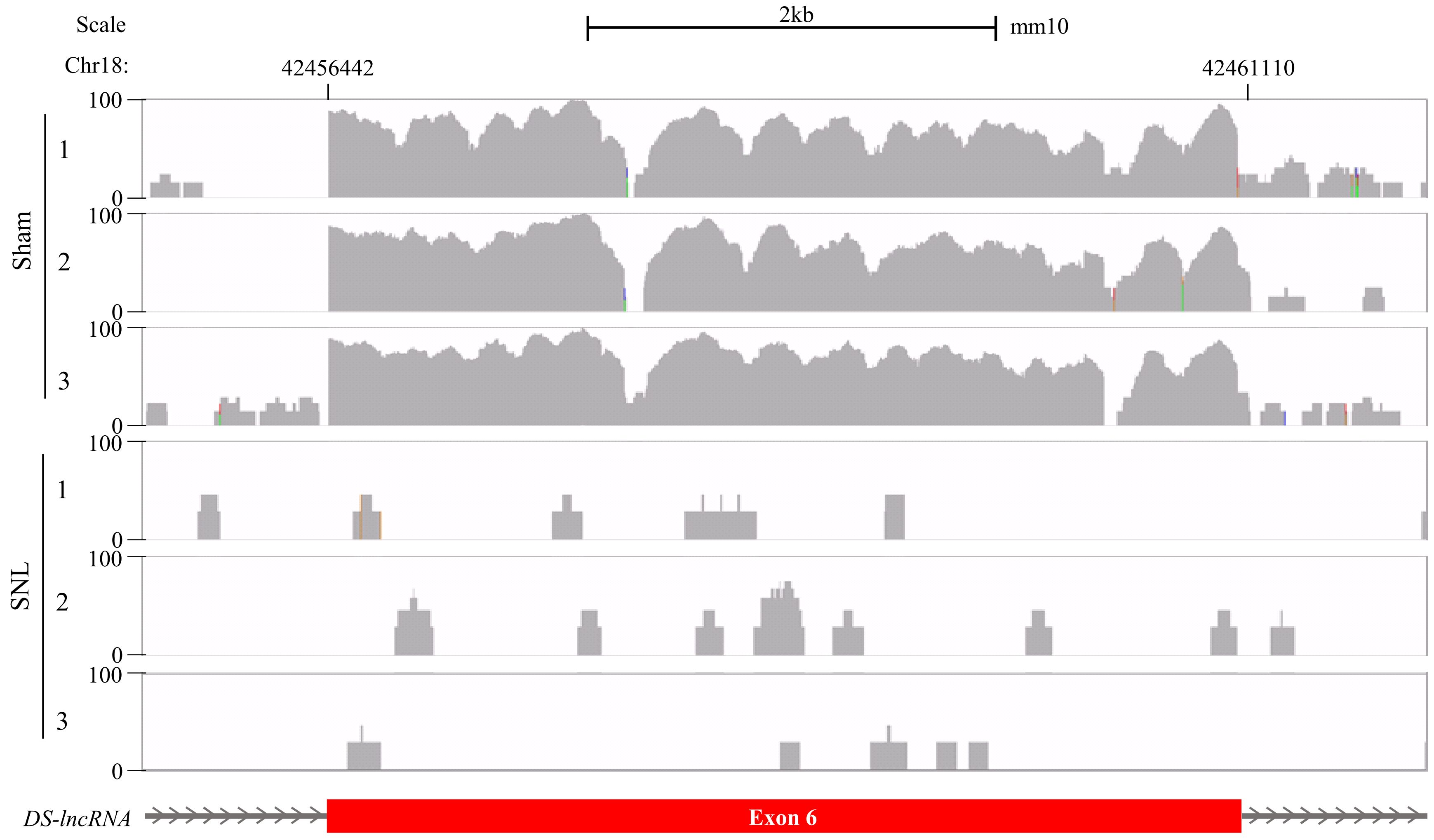

Supplementary Figure 1. Next-generation RNA-seq with a higher sequencing depth and without mRNA poly-A tail selection was carried out. The stacked reads decreased in the exon 6 region of *DS-lncRNA* gene in injured dorsal root ganglia from SNL mice compared to those from sham mice on day 7 post-surgery. n = 3 mice/group.

5'-GAAGCGAAGCGCTCCAGACGGGCGCGCCTTTGCCGGCTGCTGGGTTTCCTGCAGTGAGAACTTAAGTGTGGTGTAGTTTTGAAAGCAACATTTACCTCTAAGGATGCTGTGAGATCAATCAGACGTTCTCTTCTCATCTC
CTTGCTGCCTGCAGCTTGACAGCCTCTCTGTCCTCCTCTGCCTCTCCTTTTCCTCCCAACCCCATCCACTCCTCTTCCTTTTCTCTTCAGAGAAAACCAGCCCTGGAATATGACAGTGGTGACTTTTGAACAACATTGTGACTGT
CATCAGTCCTTTCTACCCAAGGCTTCTCAACCAATATTTTTAGAACAACTCGCAGCTATCCTGCATACATGTGGATATTTTTCTGGCTGTCTATTCTCTAAACAAATTGGCATCAATTCTGCTATTTACCTATATACTATATGAGGC
TAATTAATTAATTAATCTATAGATCAAACAAAATTTGGTTCTCAGTTTAAAATGGTTCGACTCTGGATTTTTCGACTTCATGATAGTGTGAAAAATGGCATTCATTCAGCAGATATCACCCTGGAGGGAGTTTCAATATTTCTCCAA
GCTAGTCATATTTGGTGTGATTCCCTCTTGTAACAGCAGTGACCTGGACATCCAGTCAGGCACAGGACCTGGGGGACAGCAGTTTCCTTTTATTTTGGAGGGAAACACGTTAAGTCATGGGAGATTAAAAAGGAGGTGTAGTAA
GAGGCTGTTCAAAGTGGAGGTGAAGCATTTTGTGTTCAGGGAAGCTTGTTAAACTCTGGGAGTAAACAACCCAAGGACTTCAAGAAATGGAGTGGAAGTTTCTGGTTGGGTCAAGTGATGCAGACTCAAATGGTGAGGCCAAC
TCTCCTGGTGTTTTCCTGAGGCCATGTGATGTTTGAAGAAACTATAAATAGGACTCAACCGGACAGTGACTGTGCGCTTGCACAGCTAGCCTTGCAAGGCTTCGTTGGTTTCCCATCTTCATCTTTGCCTTCGCT(2F)GATCTTC
ACTTCGTTGAGCGAGGCCCAGCAAGGAAGTTCCTGAAAAGAACTGGATTCACCAGGTCTCTCCCTCACTACACCTATGTAAGTGGTAATGATTGCTGAAGACACTCTCAGGTGTGCAGAATGTCCTAGAAGACCCTAGACCAG
AGGATCAGCGTGGAGGAGACAGAGACTGGCTCAACTGTATAGAAGAAACAAAGACCAGCCTAGGTACCTGGAAGAGGTTCGGATCCACTGAGCCACCTGCAAAGGTCCTGTTGAGTTGCCCACAGTTGTGCCGTGAGCTCCA
GGTCTCCAGGTCTTGCAAGCTGTCAACCATGATGGGGTAGCCTTTGGTCATACAGCTCTCTGGAAGAAGAAAATTGGCTTACATCCTGTCTGTGTGACAGACACAGTCAAAATCATCTATGATGCTCGTCTTCAAACCAAACAA
CAGGATGCCTCCCAATCCCATGTGAGCAGGAAAAATACAGAGAGTATGCAGACTTGTCAATTATTCCTTAGATTCCCATCTGTGACTTGATAAATATTTGATGCCGTGGCCAGTCAACATCCCTACATTAGCGAAACTTCTGCCC
AGGAGCAAAAACTGCACAGCTCTCAGCAATGAGACCCGACCTAAGTATTTCTGTCTATGAACGCCATTGTTGGGGGAAAATAACTGTGAAGATGGATGGAGGACGGCATAGCAACAGCAGCAGTAACAGCAACAGCAGCAGC
AGCAGCAGCAGCAACAGCAACAGTAACAACAGCAACAGCAACAGCAACAGCAGCAGCAGCAGCAACAGCAACAGTAACAACAGCAACAGCAACAGCAGCAGCAGCAGCAGCAGCAACAGCAACAGTAACAACAGCAACAGC
AACAACAGCAGCAACAGCAGGCTTTGTCTTTTCACTTCAGACTATAAGAAAGTTTCTACAATTTCTCCTTTTTAAAAAACTGTTTCACCATGCTACAAAATGCTAATTTTCTGCCATTTTTTACAATGAAAAAAATCAATAATCAATG
GTTTGAATTTAGCTTGCTGTCCTGGAGACACGCTCCTGAGTTCAGAAGGGTTGAGCCATGTGTTACCAGGCTCCTTCCGATGAGGATTTATAAAGATTGAGAGCTTTTTTCTTCCTTCCCAGATTCACCAGTTTCCCTTGTAATTT
ATCTTTGGGTAGCGAAGAGATTCCTTATAATTTGTCTTTGGGTAATAGATCTATCTCCGATCTGCTCCTTCTGAGTTGTGAATTAGATACAAAGAAAACATGCTTTTAAAAAAAAAACCCTTTTTTGGATCCAGAAGAAAAACATTC
ATTAGTAAAGAATATAAGGCGAGAGGGGATTTTCATCTGTCCTGTGGATTTGCTCTTATTTTAATTACTTTGCTCACTAAAAAGTTTATAAGACTTCCTTGTGACTGTTAGGTCGGCTAGGCTAGGCAGAAGGCTACTCTCTGCCA
TAGTTTACCCTGTACAAGTGTGAGGGGATACATTTCTGAACATACTCTGTTTCTTTTTCTGCCTTTATTTTGAGCTCCACTTTCGGGCTTTATTATTCTGCGTCCCCCTTCCCCTTCTCACTTTGTTTCCATTTTCAATGATACAAAG
GAATAAAAGAGGTGCTGGGGAGATAGCTAAGTGGTAACAGTGCTTTCCGTGAAAGCATGAGGACATGAGTTCAAATCCCAGCACATACATAAAAAGGCGGGTGTGACTGTGCATGTGACTATAACCCAGACCAGAGGGAGGA
AGAGACAGAGGAATCCTTGGGGCTTGCTGCCCAAGTTACAGACTAAGTGACAGACCTGTCTCAAGGGAAAGAGTGATAGAGCTGGGTACCCAGCATCCTCCTCTGGCTCCTGAGCATGCACACATGGTGTATGCAACCACAG
ACACACACATAGACACACACACAGACACACACACAGACACTCACATGCCCACATGCGCACACAATTAAAAGATAATACTCAAAGGGCTCCAAGCTGAGTATACCCTACCAAAAATGTATTTGAAGTAAATACTGAGATATCAGAA
AATTAAACATATTTTCATTAACTAGAACATCAAGGTTATTTTTCTTCTAAAACATCAAAATTCTCAATTAGTATATAATAGCAAAATACAAGTAGTAATGAATGAATCTTTACATAAATTAAAATTTTAAATAAGAGTAATTGTTTAGTT
CTAAATAAAATTTTAGTAAATATTTTTATAAATATAAATTATTTGCAATTTTTATGCTCAAAACACATCAGTTGCTAAGTAAAAAATATAAACCGATAATGTCATTGTGCTGGCTAGCTCTTTGTCAACTTGATACAAGCGAGGGTCAT
CTGGAAAGAGGGAGCCTCATTTGAGAAACTACCCACTTCTCATTTTTGTATTAGGTACTTTTTAATTGCTGTGCTAAGATTCTATGACCAAGGCCACTTAGAGAAGAAAGAGTTGATTGGGGGCTTGTGGTTTCAGAGGGTGAGT
CCATGACCACCATGGCAGAGAGTGTGGCAGCATGCAAGCAGGCATGGCACCAGAGAAGTAGCTGAGAACTTAATTCTTGAGACACGGCCATGAGGGAAAGAGAGAGAGAGAGACATACAAAGAGATACAGAGAGAATCACTA
ACTGGAAATGGTGTGGGTTTTTGAAATCTCAAAGCCCATGTCTACCAGTGACTTACTTACTTCCTCCAGGGAACCCTCCCAAACAGCTCTACCAATTTATTTATTTATTTATTTATTTATTTATTTTGGTTTTTCGAGACAGGTTTTC
TCTGTGTAGCCCTGGCTGTGCCGGAACTCACTTTGTAGACCAGGCTGGCCTCAAACTCAGAAATCCCGGAACTCACTTTGTAGACCAGGCTGGCCTCAAACTCAGAAATCCGCCTGCCTCTGCCTCCCGAGTGCTAGGATTAA
AGTTGTGCACCACCACGCCCAGAGAGTTCTACCGATTTTAGAACAACTATATGAGCCTATGGGGTCATTCTCATTCAAGTCACCACATGCCCCTACCAGACTGGCCAGCCAGTAGGCAAGTCTGTGGGACATTTTCTTGATTAA
TGATTTATATGGAAGAACCCAGCCCACTGTGGGCAGTGCTGCTCCTGGGCAGATGGTCCTGGGTGGTATAAGAAAGCAGGCTGAGCAAGACATAAAGAGCAAGGCAGTAAGCAAGTTTCCTCCATGTCCTCTGCTTCAGTTTC
TGCTTCCCGGTTCCTACGTTGGGTGAGTTCCTGCTCTGACTCCCTGGATGAACTATAAGTTGTAAGCTGAAATAAACTCTGTCTTCTTCAAGTTGCTCTGGTCAAAGTGTTTAATCCCCGTTATCGAAAGCAAGCTATGACAGCT
CTGCTCCATGCTTCCAGCAGGCTGTGTCTATACTGTCTCTAAAGACTAAGAGTAAATGTTTAATATTGCAAAGTGTTCTTTGGCCACCATGTGTAGCTGTTGCAGACACTGCACTCATTAGTCTGTGACTGCTTTTGGGTCCACA
AATTGGAACTGGAAGAAGCAAGAAAGTGGATGCTCATCACCCCTGGGAAACAATACTTTATTATGCAGGAATCTATAGCATTCGTGACATCTGGGAGGAAGGATTTGACAGGTTAATTAATTTGTCTTTTCTCCTGCCTAAATGC
TGCCACAGCTCAAATTCTCCTCCTTAGGATGGTCCTTCCGTGGCATTGGCAGCAACACAAGCCACAACCTTCATGACATTTGTAGAAAGTCACCTTCTGCTTCCAGGACAAAAGGCAAGAAAGAAAAAGGAAGAAGGCAAGTAC
ATTTGAAGTGGCCTGGATGGTACACACTGGGGGCCATGGCTGCATCATTCAGAGGGCACCCATGCTATTTAGTAAACTTGCCTTTCATTGTGAGTTTGTGATGCTGGACTCTAGGAAGAATGTTCAGGGGAGAGCCTCTTGTC
CTGTCTAAAGGCTGTCCCAAGCTCGATATAAATCCAGTACCACCAATAGGATAACCCCATGTGGAGAGTAGACAGGGACCCCACCCACAAGTACCCAAATCAATTATGATATCAACATTTTTGGCATTTGTGTTTCTATAAAAAT
GATACTAGCATGCACACATTATTTTATGTATTTATTATTATATTTTTTTCCACGACAAAGTTTCTCTGTAAATCCCTGGCTGTCCTAGAACTTGCTTTGTAGACCAGGCTGGCCTCAAACTCAGGGATCTACCTTCATCTGCCCAGC
TTTGTGTGTGTGTGTGTGTGTGTGTGTGTGTGTGTTTTAAATAGACAAGGAAGATTACTATATAAAAGCTTTGATGAGCTTCATTTCCACTTAAAACATTTCATAACACTATCCATATTGCTACTAAACATTTGTTAACTTTATCTTATTA
TATCAGTAAGATTTCAACCATTACTTCTTGATTGACTTTTTTCTTCAAGTTTATCATTATAAAAATAATATGACAAATATCCTTTGGAGATGACCTTATTTTCTGTGTTTAAGCTCATGGCTGCTTTTGGCCAATTTTAAATAATGCAT
GAATATTAGGACCTAAATATATATATTATATCTATATATAATATGAATGAATATATTTATGTATATGCTTATATACAGTACATGTGTGCATATATAATGTATGTATATACACATATACATATATACATATGTACACATATAAATATATATACA
ACATATGCATGTTGTAAAATTGCTTAGTTCTTACTCATAGTTTTCATCTTCTGAAGAATTTTATGAACAATTAAGTTATAGATGAAAACGTTACATTAGGTAATCTGTCTGATACCCAAAGCAGATGAATACTAACTCCCAAAGGAAA
AAAATGCTAGCTACCAAAATATTTATAAATTCAGATTGATAAGAAATGTTACATGGAAATACATATTTACATACATAAATATTTCAAGTATCTTATATATTGAGACAGATAACAATATCTTTATACTATGTATACATGCTTACATATATA
ATCCATATAGACATAAGTAAGATTGGAAACATATATGTGCTTTGGAAATTGAAGCCAATCCAGGATGAAGATTCAGGTTTGGCTGATCCCAGTGTCCCTAAACCATACATTCTATGTGCAATTAGGACTCAGTGAGGCCTCAAGG
GTCATTTGAATATAGGACCAAATCTGCAAATACATCAAAAACCAGGAGTCTCAGGGGTAGAGAGGAGTTAAAGAAAAAGGTAATGGGAGCTTTTGACATTTGTGTATTTCTTAACTTACTTAAATGCTAAAAAATAGATTTATGTA
ATATGTGTGAAATCTTAAAAATTAATAAAAATATTATTTTACAT-3'

Supplementary Figure 2. The full-length sequence of mouse *DS-lncRNA* splice isoform transcript I (*SIT1*) cDNA (6.293 kb). Six exons are presented in different colors. Translation analysis with DNAMAN software shows many stop codons (underlined TAG, TGA, TAA) distributed throughout the sequence. Genomic location: Chr18: 42398415 – 42461110.

5’-GAAGCGAAGCGCTCCAGACGGGCGCGCCTTTGCCGGCTGCTGGGTTTCCTGCAGTGAGAACTTAAGTGTGGTGTAGTTTTGAAAGCAACATTTACCTCTAAGGATGCTGTGAGATCAATCAGACGTTCTCTTCTCATCTC
CTTGCTGCCTGCAGCTTGACAGCCTCTCTGTCCTCCTCTGCCTCTCCTTTTCCTCCCAACCCCATCCACTCCTCTTCCTTTTCTCTTCAGAGAAAACCAGCCCTGGAATATGACAGTGGTGACTTTTGAACAACATTGTGACTGT
CATCAGTCCTTTCTACCCAAGGCTTCTCAACCAATATTTTTAGAACAACTCGCAGCTATCCTGCATACATGTGGATATTTTTCTGGCTGTCTATTCTCTAAACAAATTGGCATCAATTCTGCTATTTACCTATATACTATATGAGGC
TAATTAATTAATTAATCTATAGATCAAACAAAATTTGGTTCTCAGTTTAAAATGGTTCGACTCTGGATTTTTCGACTTCATGATAGTGTGAAAAATGGCATTCATTCAGCAGATATCACCCTGGAGGGAGTTTCAATATTTCTCCAA
GCTAGTCATATTTGGTGTGATTCCCTCTTGTAACAGCAGTGACCTGGACATCCAGTCAGGCACAGGACCTGGGGGACAGCAGTTTCCTTTTATTTTGGAGGGAAACACGTTAAGTCATGGGAGATTAAAAAGGAGGTGTAGTAA
GAGGCTGTTCAAAGTGGAGGTGAAGCATTTTGTGTTCAGGGAAGCTTGTTAAACTCTGGGAGTAAACAACCCAAGGACTTCAAGAAATGGAGTGGAAGTTTCTGGTTGGGTCAAGTGATGCAGACTCAAATGGTGAGGCCAAC
TCTCCTGGTGTTTTCCTGAGGCCATGTGATGTTTGAAGAAACTATAAATAGGACTCAACCGGACAGTGACTGTGCGCTTGCACAGCTAGCCTTGCAAGGCTTCGTTGGTTTCCCATCTTCATCTTTGCCTTCGCT(2F)GATCTTC
ACTTCGTTGAGCGAGGCCCAGCAAGGAAGTTCCTGAAAAGAACTGGATTCACCAGGTCTCTCCCTCACTACACCTATGTAAGTGGTAATGATTGCTGAAGACACTCTCAGGTGTGCAGAATGTCCTAGAAGACCCTAGACCAG
AGGATCAGCGTGGAGGAGACAGAGACTGGCTCAACTGTATAGAAGAAACAAAGACCAGCCTAGGTACCTGGAAGAGGTTCGGATCCACTGAGCCACCTGCAAAGGTCCTGTTGAGTTGCCCACAGTTGTGCCGTGAGCTCCA
GGTCTCCAGGTCTTGCAAGCTGTCAACCATGATGGGGTAGCCTTTGGTCATACAGCTCTCTGGAAGAAGAAAATTGGCTTACATCCTGTCTGTGTGACAGACACAGTCAAAATCATCTATGATGCTCGTCTTCAAACCAAACAA
CAGGATGCCTCCCAATCCCATGTGAGCAGGAAAAATACAGAGAGTATGCAGACTTGTCAATTATTCCTTAGCCTAAGTATTTCTGTCTATGAACGCCATTGTTGGGGGAAAATAACTGTGAAGATGGATGGAGGACGGCATAGC
AACAGCAGCAGTAACAGCAACAGCAGCAGCAGCAGCAGCAGCAACAGCAACAGTAACAACAGCAACAGCAACAGCAACAGCAGCAGCAGCAACAGCAACAGTAACAACAGCAACAGCAACAGCAGCAGCAGCAGCAGC
AGCAACAGCAACAGTAACAACAGCAACAGCAACAACAGCAGCAACAGCAGGCTTTGTCTTTTCACTTCAGACTATAAGAAAGTTTCTACAATTTCTCCTTTTTAAAAAACTGTTTCACCATGCTACAAAATGCTAATTTTCTGCCAT
TTTTTACAATGAAAAAAATCAATAATCAATGGTTTGAATTTAGCTTGCTGTCCTGGAGACACGCTCCTGAGTTCAGAAGGGTTGAGCCATGTGTTACCAGGCTCCTTCCGATGAGGATTTATAAAGATTGAGAGCTTTTTTCTTCC
TTCCCAGATTCACCAGTTTCCCTTGTAATTTATCTTTGGGTAGCGAAGAGATTCCTTATAATTTGTCTTTGGGTAATAGATCTATCTCCGATCTGCTCCTTCTGAGTTGTGAATTAGATACAAAGAAAACATGCTTTTAAAAAAAAA
ACCCTTTTTTGGATCCAGAAGAAAAACATTCATTAGTAAAGAATATAAGGCGAGAGGGGATTTTCATCTGTCCTGTGGATTTGCTCTTATTTTAATTACTTTGCTCACTAAAAAGTTTATAAGACTTCCTTGTGACTGTTAGGTCGG
CTAGGCTAGGCAGAAGGCTACTCTCTGCCATAGTTTACCCTGTACAAGTGTGAGGGGATACATTTCTGAACATACTCTGTTTCTTTTTCTGCCTTTATTTTGAGCTCCACTTTCGGGCTTTATTATTCTGCGTCCCCCTTCCCCTT
CTCACTTTGTTTCCATTTTCAATGATACAAAGGAATAAAAGAGGTGCTGGGGAGATAGCTAAGTGGTAACAGTGCTTTCCGTGAAAGCATGAGGACATGAGTTCAAATCCCAGCACATACATAAAAAGGCGGGTGTGACTGTGC
ATGTGACTATAACCCAGACCAGAGGGAGGAAGAGACAGAGGAATCCTTGGGGCTTGCTGCCCAAGTTACAGACTAAGTGACAGACCTGTCTCAAGGGAAAGAGTGATAGAGCTGGGTACCCAGCATCCTCCTCTGGCTCCTG
AGCATGCACACATGGTGTATGCAACCACAGACACACACATAGACACACACACAGACACACACACAGACACTCACATGCCCACATGCGCACACAATTAAAAGATAATACTCAAAGGGCTCCAAGCTGAGTATACCCTACCAAAAA
TGTATTTGAAGTAAATACTGAGATATCAGAAAATTAAACATATTTTCATTAACTAGAACATCAAGGTTATTTTTCTTCTAAAACATCAAAATTCTCAATTAGTATATAATAGCAAAATACAAGTAGTAATGAATGAATCTTTACATAAAT
TAAAATTTTAAATAAGAGTAATTGTTTAGTTCTAAATAAAATTTTAGTAAATATTTTTATAAATATAAATTATTTGCAATTTTTATGCTCAAAACACATCAGTTGCTAAGTAAAAAATATAAACCGATAATGTCATTGTGCTGGCTAGCT
CTTTGTCAACTTGATACAAGCGAGGGTCATCTGGAAAGAGGGAGCCTCATTTGAGAAACTACCCACTTCTCATTTTTGTATTAGGTACTTTTTAATTGCTGTGCTAAGATTCTATGACCAAGGCCACTTAGAGAAGAAAGAGTTG
ATTGGGGGCTTGTGGTTTCAGAGGGTGAGTCCATGACCACCATGGCAGAGAGTGTGGCAGCATGCAAGCAGGCATGGCACCAGAGAAGTAGCTGAGAACTTAATTCTTGAGACACGGCCATGAGGGAAAGAGAGAGAGAGA
GACATACAAAGAGATACAGAGAGAATCACTAACTGGAAATGGTGTGGGTTTTTGAAATCTCAAAGCCCATGTCTACCAGTGACTTACTTACTTCCTCCAGGGAACCCTCCCAAACAGCTCTACCAATTTATTTATTTATTTATTTA
TTTATTTATTTTGGTTTTTCGAGACAGGTTTTCTCTGTGTAGCCCTGGCTGTGCCGGAACTCACTTTGTAGACCAGGCTGGCCTCAAACTCAGAAATCCCGGAACTCACTTTGTAGACCAGGCTGGCCTCAAACTCAGAAATCC
GCCTGCCTCTGCCTCCCGAGTGCTAGGATTAAAGTTGTGCACCACCACGCCCAGAGAGTTCTACCGATTTTAGAACAACTATATGAGCCTATGGGGTCATTCTCATTCAAGTCACCACATGCCCCTACCAGACTGGCCAGCCA
GTAGGCAAGTCTGTGGGACATTTTCTTGATTAATGATTTATATGGAAGAACCCAGCCCACTGTGGGCAGTGCTGCTCCTGGGCAGATGGTCCTGGGTGGTATAAGAAAGCAGGCTGAGCAAGACATAAAGAGCAAGGCAGTAA
GCAAGTTTCCTCCATGTCCTCTGCTTCAGTTTCTGCTTCCCGGTTCCTACGTTGGGTGAGTTCCTGCTCTGACTCCCTGGATGAACTATAAGTTGTAAGCTGAAATAAACTCTGTCTTCTTCAAGTTGCTCTGGTCAAAGTGTTTTA
ATCCCCGTTATCGAAAGCAAGCTATGACAGCTCTGCTCCATGCTTCCAGCAGGCTGTGTCTATACTGTCTCTAAAGACTAAGAGTAAATGTTTAATATTGCAAAGTGTTCTTTGGCCACCATGTGTAGCTGTTGCAGACACTGCA
CTCATTAGTCTGTGACTGCTTTTGGGTCCACAAATTGGAACTGGAAGAAGCAAGAAAGTGGATGCTCATCACCCCTGGGAAACAATACTTTATTATGCAGGAATCTATAGCATTCGTGACATCTGGGAGGAAGGATTTGACAGG
TTAATTAATTTGTCTTTTCTCCTGCCTAAATGCTGCCACAGCTCAAATTCTCCTCCTTAGGATGGTCCTTCCGTGGCATTGGCAGCAACACAAGCCACAACCTTCATGACATTTGTAGAAAGTCACCTTCTGCTTCCAGGACAAAA
GGCAAGAAAGAAAAAGGAAGAAGGCAAGTACATTTGAAGTGGCCTGGATGGTACACACTGGGGGCCATGGCTGCATCATTCAGAGGGCACCCATGCTATTTAGTAAACTTGCCTTTCATTGTGAGTTTGTGATGCTGGACTCT
AGGAAGAATGTTCAGGGGAGAGCCTCTTGTCCTGTCTAAAGGCTGTCCCAAGCTCGATATAAATCCAGTACCACCAATAGGATAACCCCATGTGGAGAGTAGACAGGGACCCCACCCACAAGTACCCAAATCAATTATGATAT
CAACATTTTTGGCATTTGTGTTTCTATAAAAATGATACTAGCATGCACACATTATTTTATGTATTTATTATTATATTTTTTTCCACGACAAAGTTTCTCTGTAAATCCCTGGCTGTCCTAGAACTTGCTTTGTAGACCAGGCTGGCCT
CAAACTCAGGGATCTACCTTCATCTGCCCAGCTTTGTGTGTGTGTGTGTGTGTGTGTGTGTGTGTTTTAAATAGACAAGGAAGATTACTATATAAAAGCTTTGATGAGCTTCATTTCCACTTAAAACATTTCATAACACTATCCATA
TTGCTACTAAACATTTGTTAACTTTATCTTATTATATCAGTAAGATTTCAACCATTACTTCTTGATTGACTTTTTTCTTCAAGTTTATCATTATAAAAATAATATGACAAATATCCTTTGGAGATGACCTTATTTTCTGTGTTTAAGCTC
ATGGCTGCTTTTGGCCAATTTTAAATAATGCATGAATATTAGGACCTAAATATATATATATTATATCTATATATAATATATGAATGAATATATTTATGTATATGCTTATATACAGTACATGTGTGCATATATAATGTATGTATATACACATATA
CATATATACATATGTACACATATAAATATATATACAACATATGCATGTTGTAAAATTGCTTAGTTCTTACTCATAGTTTTCATCTTCTGAAGAATTTTATGAACAATTAAGTTATAGATGAAAACGTTACATTAGGTAATCTGTCTGATAC
CCAAAGCAGATGAATACTAACTCCCAAAGGAAAAAAATGCTAGCTACCAAAATATTTATAAATTCAGATTGATAAGAAATGTTACATGGAAATACATATTTACATACATAAATATTTCAAGTATCTTATATATTGAGACAGATAACAA
TATCTTTATACTATGTATACATGCTTACATATATAATCCATATAGACATAAGTAAGATTGGAAACATATATGTGCTTTGGAAATTGAAGCCAATCCAGGATGAAGATTCAGGTTTGGCTGATCCCAGTGTCCCTAAACCATACATTC
TATGTGCAATTAGGACTCAGTGAGGCCTCAAGGGTCATTTGAATATAGGACCAAATCTGCAAATACATCAAAAACCAGGAGTCTCAGGGGTAGAGAGGAGTTAAAGAAAAAGGTAATGGGAGCTTTTGACATTTGTGTATTTCTT
AACTTACTTAAATGCTAAAAAATAGATTTATGTAATATGTGTGAAATCTTAAAAATTAATAAAAATATTATTTTACAT-3’

Supplementary Figure 3. The full-length sequence of mouse *DS-lncRNA* splice isoform transcript II (*SIT2*) cDNA (6.18 kb). Five exons are presented in different colors. Translation analysis with DNAMAN software shows many stop codons (underlined TAG, TGA, TAA) distributed throughout the sequence. Genomic location: Chr18: 42398415 – 42461110.

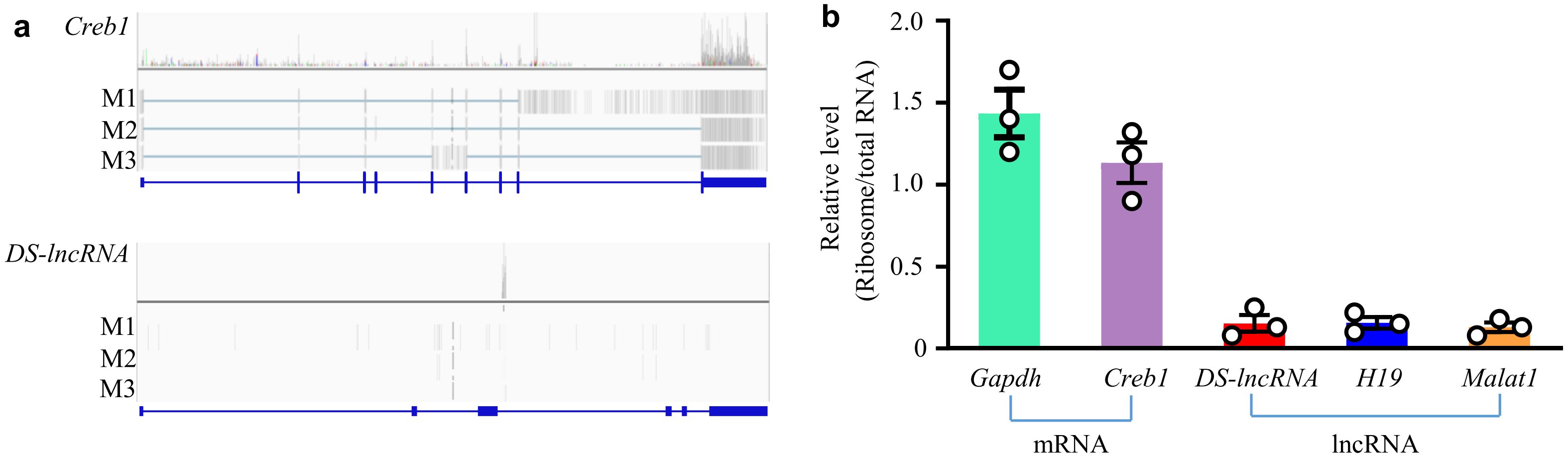

Supplementary Figure 4. Identification of *DS-lncRNA* as a non-coding RNA. (**a**) The ribosome profiling of *DS-lncRNA* and *Creb1*. The blue rectangles represent their corresponding exons. n = 3 mice (M). (**b**) Signal ratios of ribosome profiling to RNA sequencing for mRNAs (*Gapdh* and *Creb1*) and long noncoding RNAs (*DS-lncRNA, H19* and *Malat1*). n = 3 mice.

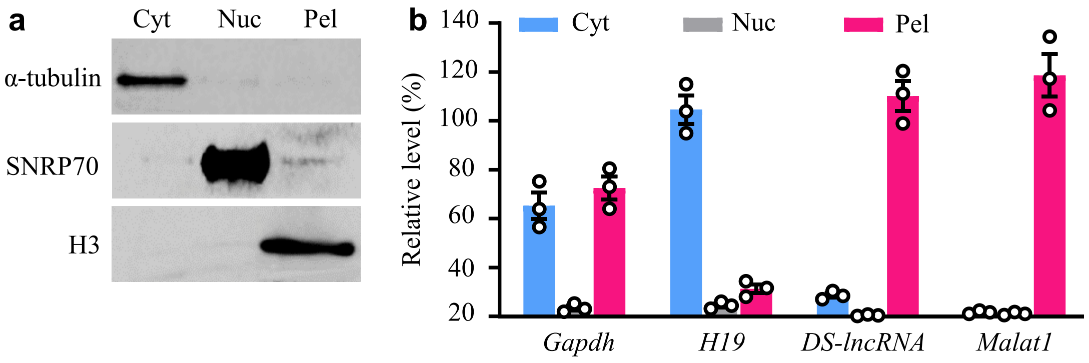

Supplementary Figure 5. Subcellular distribution of *DS-lncRNA* in the dorsal root ganglion (DRG). (**a**) Identification of cytoplasmic soluble (Cyt), nuclear soluble (Nuc) and insoluble pellet (Pel) fractions, which fractionated from the cultured DRG neurons of adult mice. α-tubulin: a marker of cytoplasmic soluble fraction. SNRP70: a marker of nuclear soluble fraction. H3: a marker of nuclear insoluble pellet fraction. (**b**) Levels of *Gapdh* mRNA*, H19, DS-lncRNA* and *Malat1* expression in cytoplasmic soluble (Cyt), nuclear soluble (Nuc) and insoluble pellet (Pel). n = 3 mice.

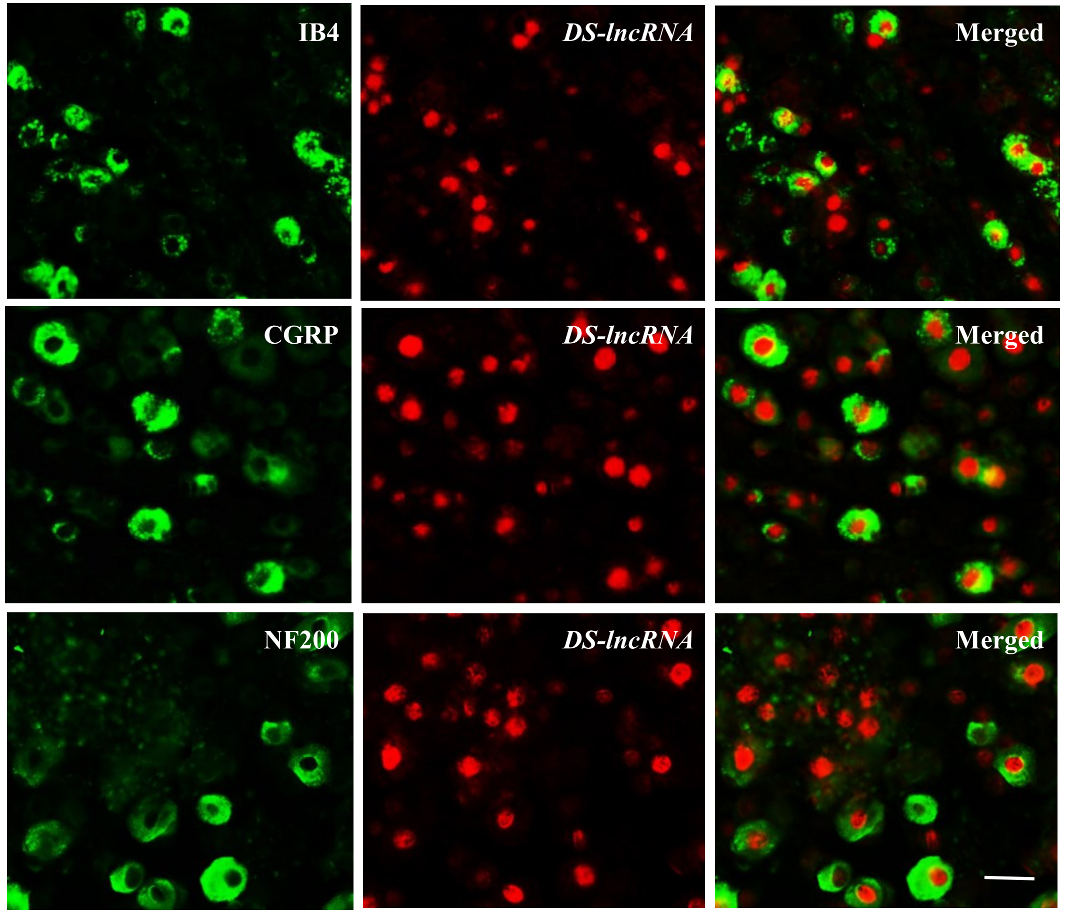

Supplementary Figure 6. Subpopulation of distribution of *DS-lncRNA*-containing neurons in DRG of naive mice. Neurons were double-labeled for *DS-lncRNA* (red) and for isolectin B4 (IB4, green), calcitonin gene-related peptide (CGRP, green), or neurofilament-200 (NF200, green). n = 5 mice. Scale bar: 50 µm.

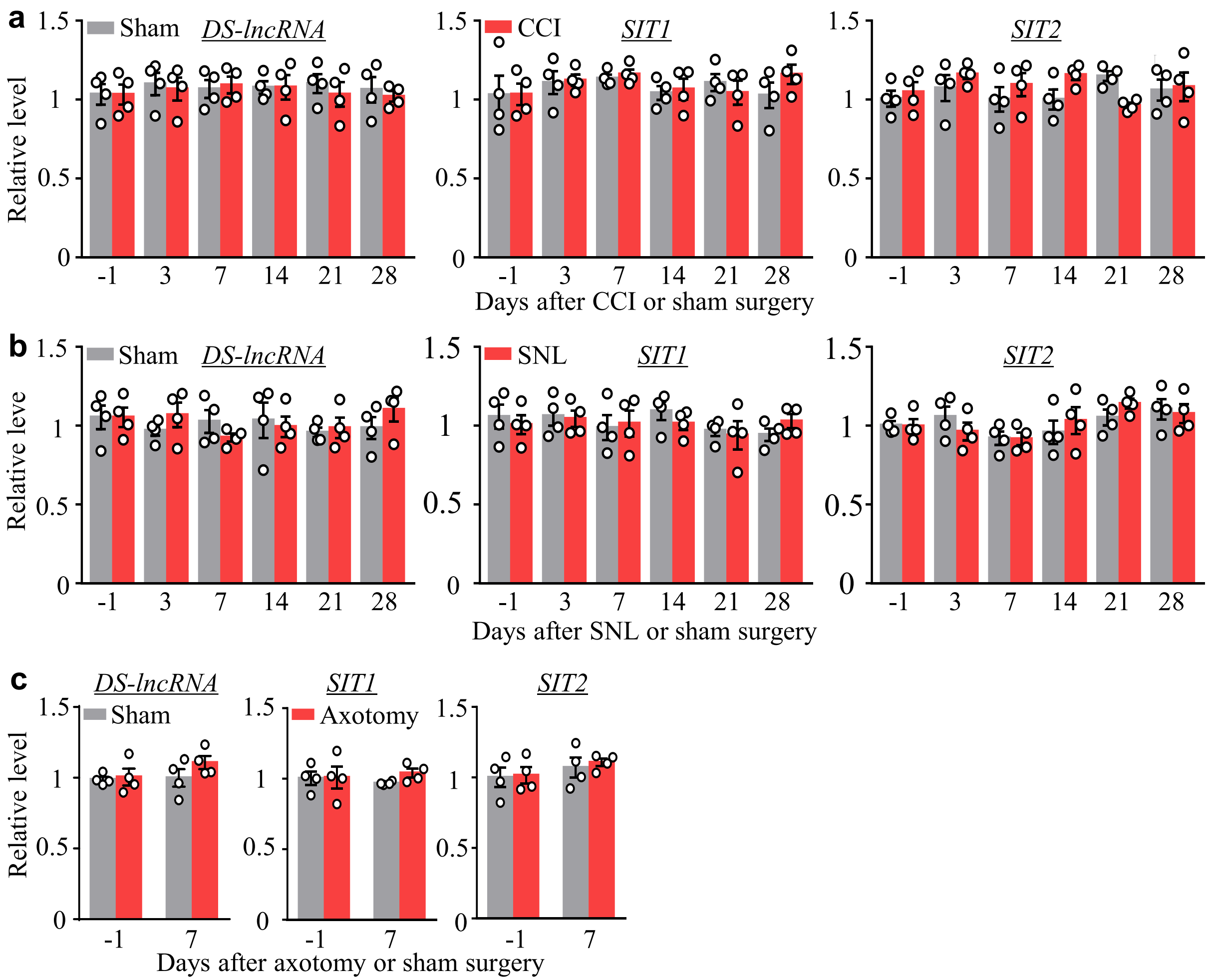

Supplementary Figure 7. The levels of *DS-lncRNA*, *SIT1* and *SIT2* expression in the contralateral dorsal root ganglion (DRG) after peripheral nerve injury. (**a**) Levels of *DS-lncRNA*, *SIT1* and *SIT2* in the contralateral L3/4 DRGs after chronic constriction injury (CCI) or sham surgery of unilateral sciatic nerve. n = 8 mice/time point/group. (**b**) Levels of *DS-lncRNA*, *SIT1* and *SIT2* in the contralateral L4 DRG after unilateral spinal nerve ligation (SNL) or sham surgery. n = 16 mice/time point/group. (**c**) Levels of *DS-lncRNA*, *SIT1* and *SIT2* in the contralateral L3/4 DRGs on day 7 after axotomy or sham surgery of unilateral sciatic nerve. n = 8 mice/time point/group. Two-way ANOVA with repeated measures followed by post hoc Tukey test.

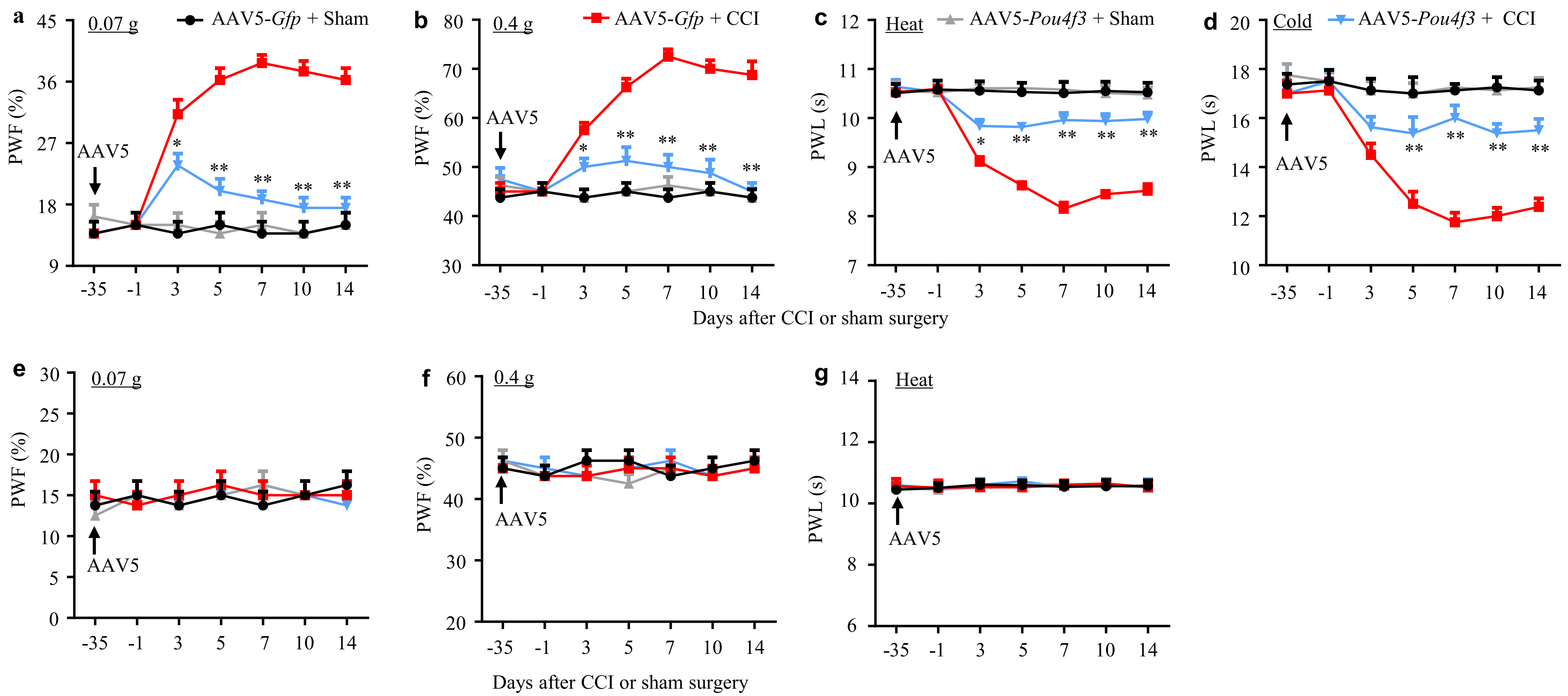

Supplementary Figure 8. Rescuing nerve injury-induced Pou4f3 downregulation in injured DRG attenuated the CCI-induced neuropathic pain induction. Effect of pre-microinjection of AAV5-*Pou4f3* or AAV5-*Gfp* into the ipsilateral L3/4 DRG of mice on paw withdrawal frequency (PWF) to 0.07 g (**a**, **e**) and 0.4 g (**b**. **f**) von Frey filaments and on paw withdrawal latencies (PWL) to heat (**c**, **g**) and cold (**d**) stimuli on the ipsilateral (a-d) and contralateral (e-g) sides at the different days after CCI or sham surgery. n = 8 mice/group. *$P < 0.05$, **$P < 0.01$ versus the AAV5-*Gfp* plus CCI group at the corresponding time points by two-way ANOVA with repeated measures followed by post hoc Tukey test.

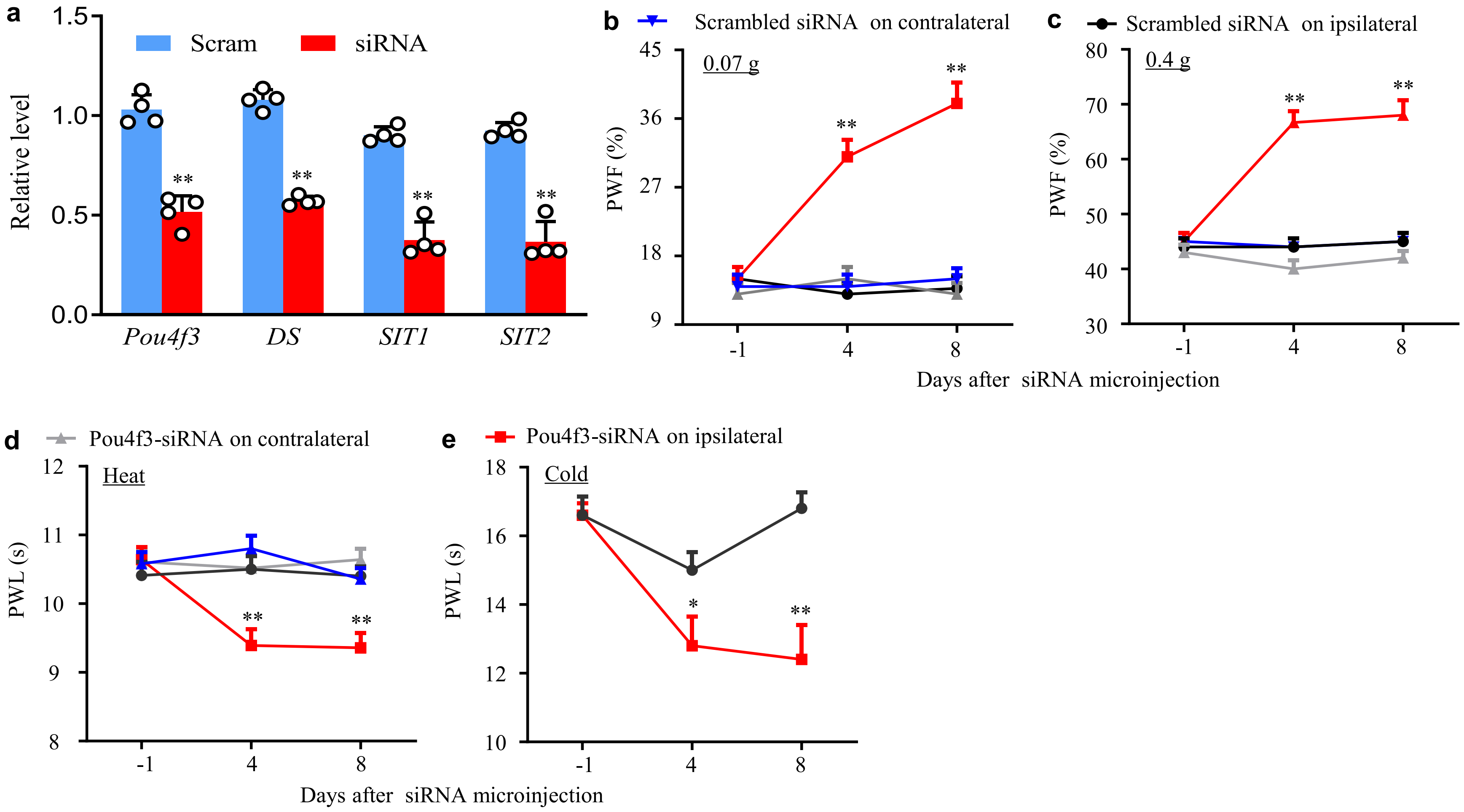

Supplementary Figure 9. DRG knockdown of Pou4f3 produced neuropathic pain-like symptoms. (**a**) Levels of *Pou4f3* mRNA (Pou4f3), *DS-lncRNA* (DS), *SIT1* and *SIT2* in the ipsilateral L3/4 DRGs on day 4 after DRG microinjection of *Pou4f3* siRNA (siRNA) or control scrambled siRNA (Scram). n = 8 mice/group. ***P* < 0.01 versus the corresponding control scrambled siRNA group by two-tailed unpaired Student's t test. (**b**-**e**) Effect of pre-microinjection of *Pou4f3* siRNA or control scrambled siRNA into unilateral L3/4 DRGs on the paw withdrawal frequency (PWF) to 0.07 g (**b**) and 0.4 g (**c**) von Frey filaments and on paw withdrawal latencies (PWL) to heat (**d**) and cold (**e**) stimuli on the ipsilateral and contralateral sides at the different days after siRNA microinjection. n = 8 mice/group. **P* < 0.05, ***P* < 0.01 versus the scrambled siRNA-treated mice on the ipsilateral side at the corresponding time points by two-way ANOVA with repeated measures followed by post hoc Tukey test.

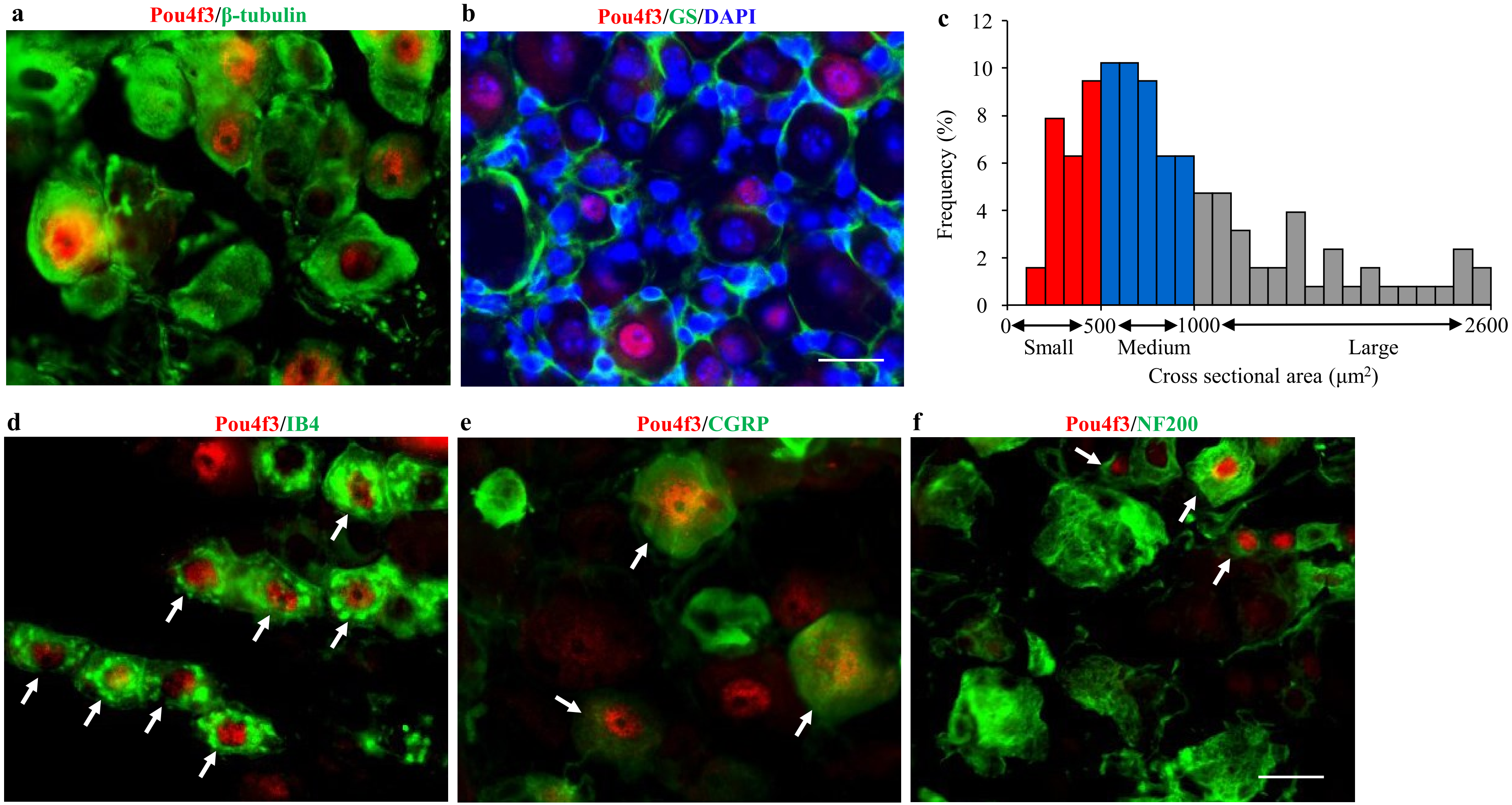

Supplementary Figure 10. Distribution of Pou4f3 in DRG. (**a-b**) Pou4f3 (red) is co-expressed with β-tubulin III (green, a) in individual cells and undetected in cellular nuclei (labeled by 4', 6-diamidino-2-phenylindole (DAPI), blue) of glutamine synthetase (GS, green, b)-labeled cells. Approximately 52 % of the β-tubulin III-labelled neurons were positive for Pou4f3. n = 3 mice. Scale bar: 25 μm. (**c**) Histogram shows the distribution of Pou4f3-positive somata in normal mouse L4 DRG: small, 25%; medium, 43%; large, 32%. (**d-f**) About 30% of the Pou4f3 (red)-positive neurons were labeled by isolectin B4 (IB4, green, d), 25% by calcitonin gene-related peptide (CGRP, green, e), and 48% by neurofilament-200 (NF200, green, f). Arrows: double-labeled neurons. n = 5 mice. Scale bar: 25 μm.

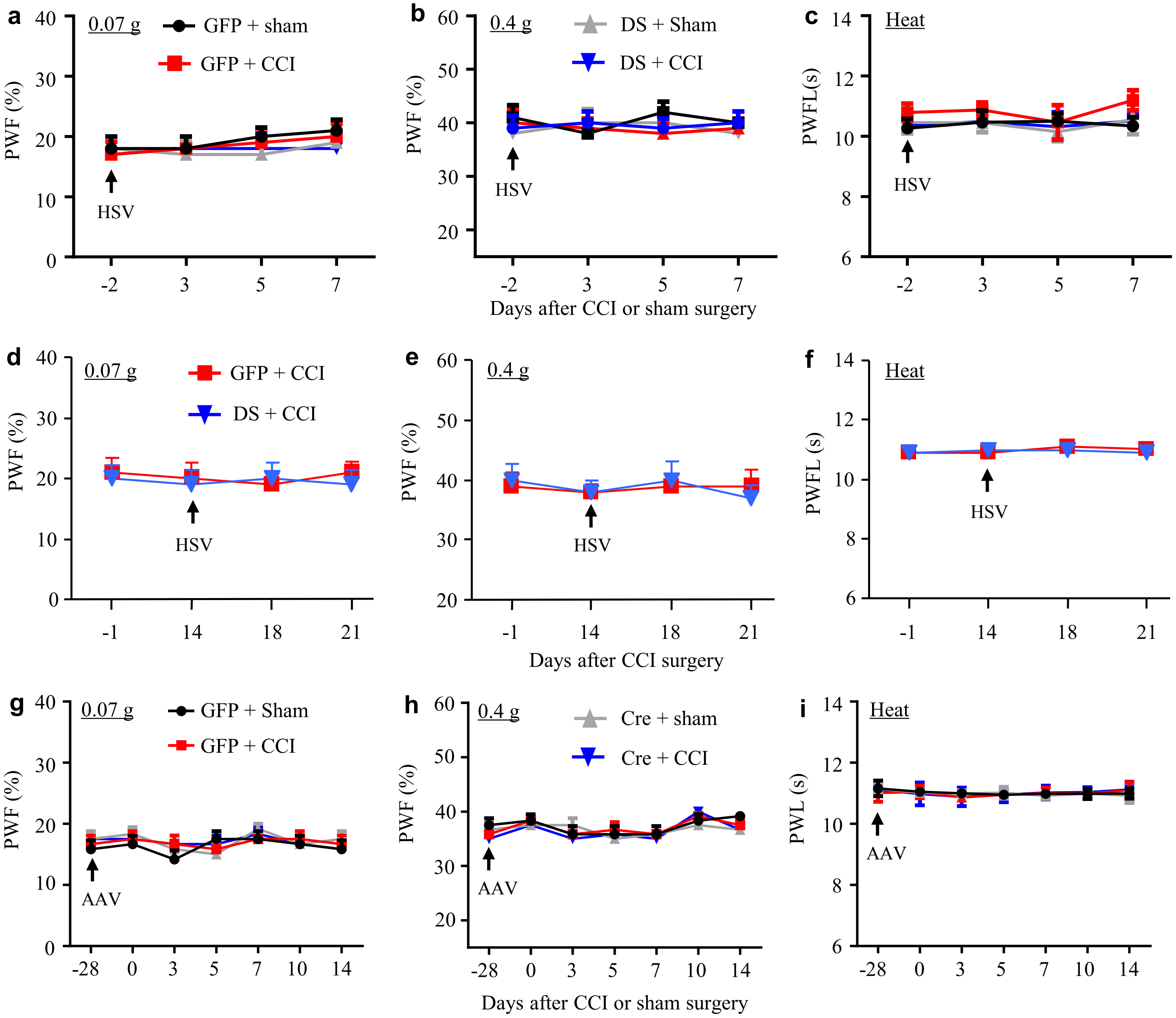

a
0.07 g
GFP + sham
GFP + CCI
PWF (%)
HSV
b
0.4 g
DS + Sham
DS + CCI
PWF (%)
HSV
c
Heat
PWFL(s)
HSV
Days after CCI or sham surgery
d
0.07 g
GFP + CCI
DS + CCI
PWF (%)
HSV
e
0.4 g
PWF (%)
HSV
f
Heat
PWFL (s)
HSV
Days after CCI surgery
g
0.07 g
GFP + Sham
GFP + CCI
PWF (%)
AAV
h
0.4 g
Cre + sham
Cre + CCI
PWF (%)
AAV
i
Heat
PWL (s)
AAV
Days after CCI or sham surgery

Supplementary Figure 11. Effect of rescuing nerve injury-induced *DS-lncRNA* downregulation in injured DRG on basal paw withdrawal responses on the contralateral side after CCI. (**a-c**) Basal paw withdrawal responses to mechanical (a, b) and heat (c) stimuli on the contralateral side during the development period at days indicated post-CCI or -sham surgery in mice pre-microinjected with HSV-*Gfp* (GFP) or HSV-*DS-lncRNA* (DS) into unilateral L3/4 DRGs for 2 days. n = 10 mice/group. Two-way ANOVA with repeated measures followed by *post hoc* Tukey test. (**d-f**) Basal paw withdrawal responses to mechanical (d, e) and heat (f) stimuli on the contralateral side during the maintenance period at days indicated post-CCI in mice post-microinjected with HSV-*Gfp* (GFP) or HSV-*DS-lncRNA* (DS) into unilateral L3/4 DRGs. n = 10 mice/group. Two-way ANOVA with repeated measures followed by *post hoc* Tukey test. (**g-i**) Basal paw withdrawal responses to mechanical (g, h) and heat (i) stimuli on the contralateral side during the development period at days indicated post-CCI or -sham surgery in conditional Rosa26$^{DS\text{-}lncRNA}$ knock-in mice (DS-KI mice) pre-microinjected with AAV5-*Cre* (Cre) or AAV5-*Gfp* (GFP) into the ipsilateral L3/4 DRGs for 28 days. n = 12 mice/group. Two-way ANOVA with repeated measures followed by post hoc Tukey test.

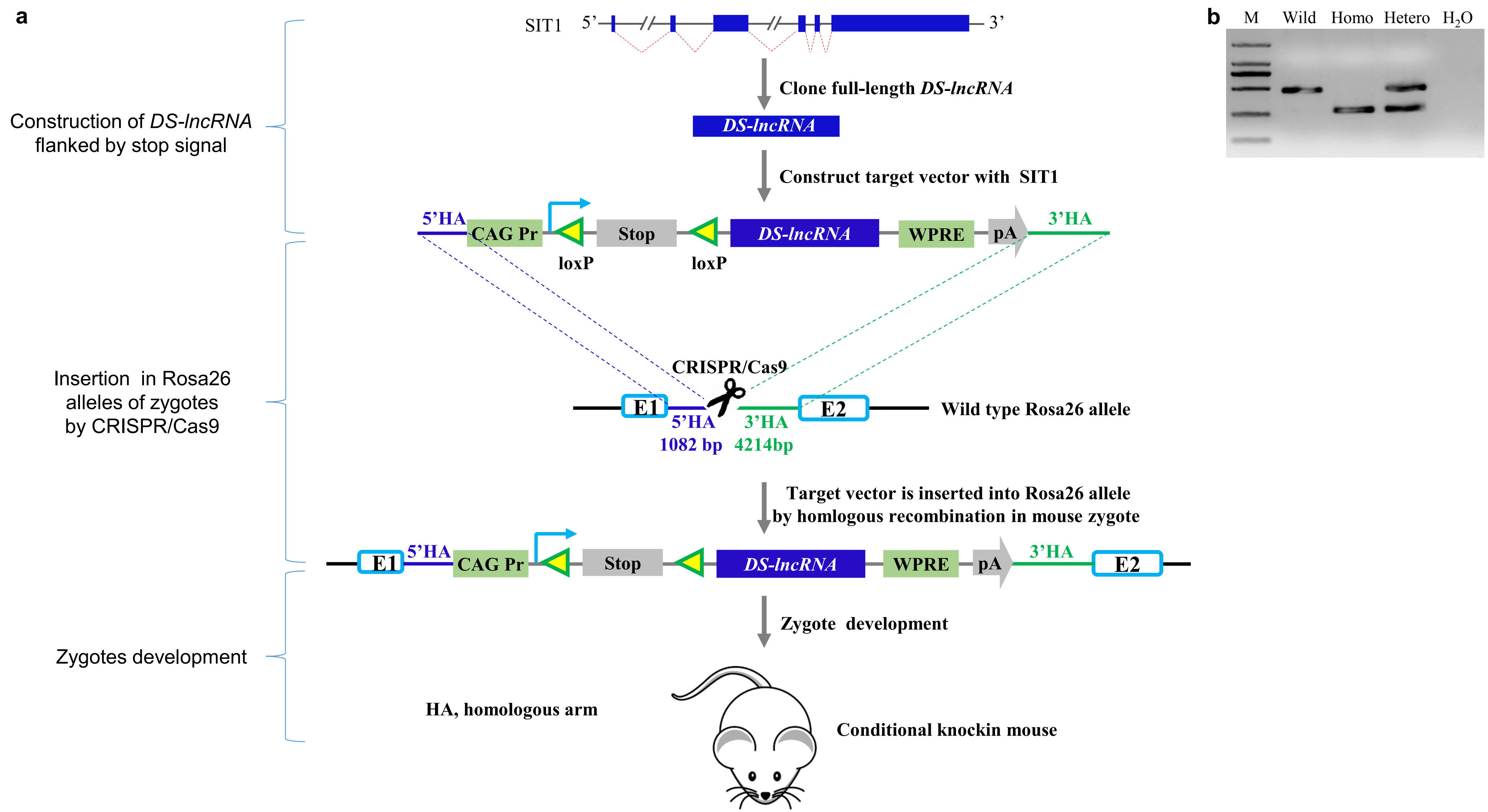

Supplementary Figure 12. Strategy for generation of conditional Rosa26$^{DS\text{-}lncRNA}$ knock-in mice (DS-KI mice). (**a**) Constructed vector containing *DS-lncRNA* flanked by stop signal was inserted into Rosa26 allele by homologous recombination in C57BL/6 mouse (Rosa26$^{\boldsymbol{DS\text{-}lncRNA}}$). HA, homologous arm. CAG Pro: CAG promoter in vector. pA: poly A. E1: *Rosa26* exon 1. E2: *Rosa26* exon 2. (**b**) Genotyping identification of conditional Rosa26$^{DS\text{-}lncRNA}$ mice by PCR. M: DNA ladder marker. Homo: Homozygous. Hetero: Heterozygous. Wild: wild type. H_2O: without DNA template.

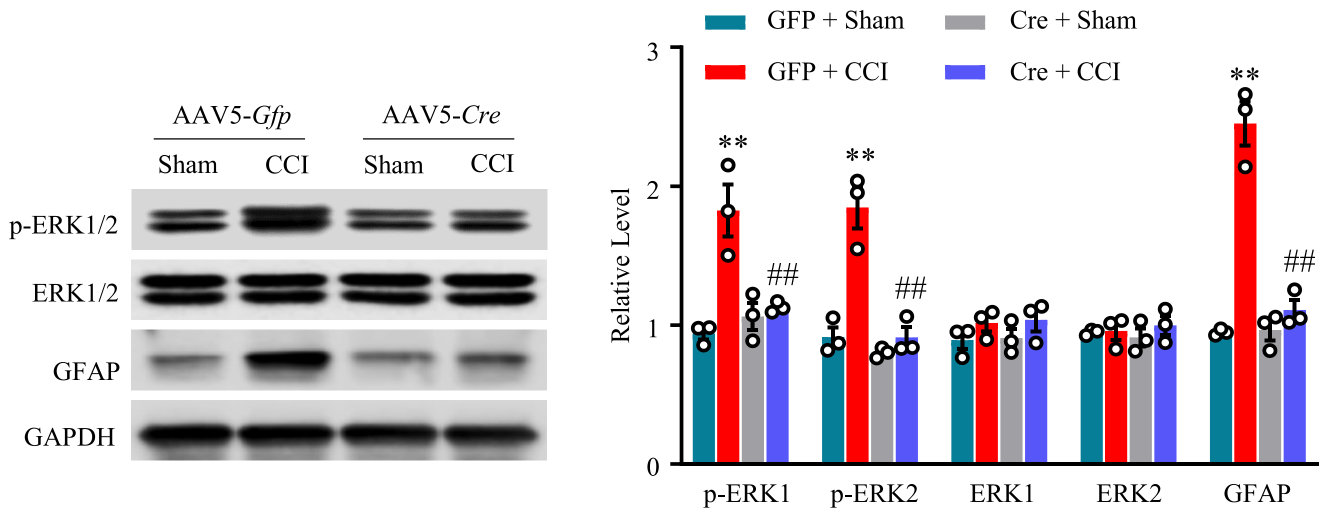

Supplementary Figure 13. Effect of rescuing nerve injury-induced *DS-lncRNA* downregulation in injured DRG on CCI-induced dorsal horn neuronal and astrocyte hyperactivities in mice. Levels of p-ERK1/2, ERK1/2 and GFAP in the ipsilateral L3/4 dorsal horn 14 days after CCI or sham surgery from conditional Rosa26$^{DS\text{-}lncRNA}$ knock-in mice (DS-KI mice) pre-microinjected with AAV5-*Cre* (Cre) or AAV5-*Gfp* (GFP) into the ipsilateral L3/4 DRGs for 28 days. n = 3 mice/group. $^{**}P < 0.01$ versus the corresponding AAV5-*Gfp*-treated sham group and $^{\#\#}P < 0.01$ versus the corresponding AAV5-*Gfp*-treated CCI group by one-way ANOVA followed by post hoc Tukey test.

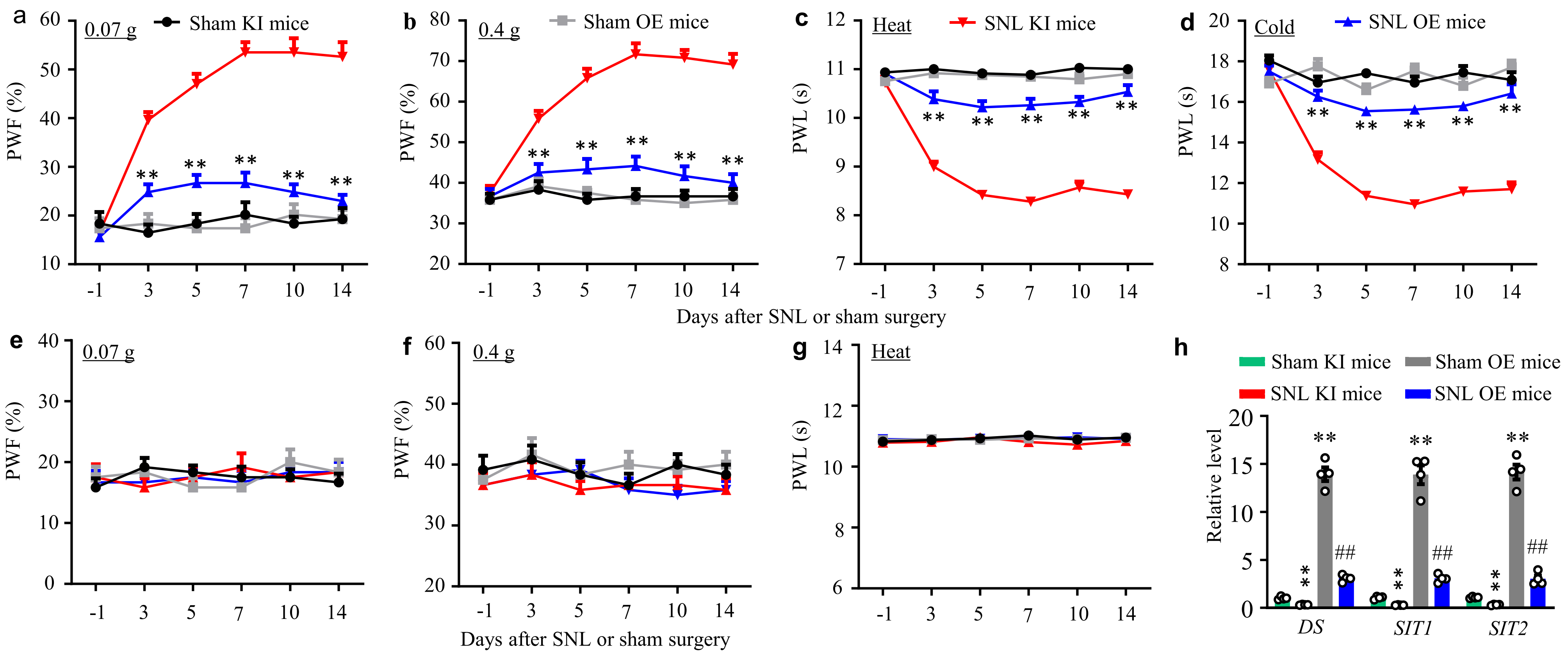

Supplementary Figure 14. Effect of DRG *DS-lncRNA* overexpression (OE) on the development of SNL-induced pain hypersensitivity. Conditional *DS*-lncRNA OE mice were generated by cross-breeding of the conditional Rosa26$^{DS\text{-}lncRNA}$ knock-in (KI) mice with sensory neuron-specific Advillin$^{Cre/+}$ mice. **(a-g)** Paw withdrawal frequency (PWF) to low (0.07 g; a and e) and medium (0.4 g; b and f) force von Frey filaments and paw withdrawal latencies (PWL) to heat (c and g) and cold (d) stimuli on the ipsilateral (a-d) and contralateral (e-g) sides from Rosa26$^{DS\text{-}lncRNA}$ KI mice or conditional *DS*-lncRNA OE mice at the different days as indicated after SNL or sham surgery. n = 12 mice/group. $^{**}P < 0.01$ versus the SNL DS-KI mice at the corresponding time points by two-way ANOVA with repeated measures followed by post hoc Tukey test. (**h**) Levels of *DS-lncRNA* (*DS*), *SIT1* and *SIT2* in the ipsilateral L4 DRG on day 14 after SNL or sham surgery in DS-KI (KI) mice or conditional *DS*-lncRNA OE mice. n = 12 mice/group. $^{*}P < 0.05$, $^{**}P < 0.01$ versus the corresponding sham KI mice and $^{\#\#}P < 0.01$ versus the corresponding SNL KI mice by one-way ANOVA followed by post hoc Tukey test.

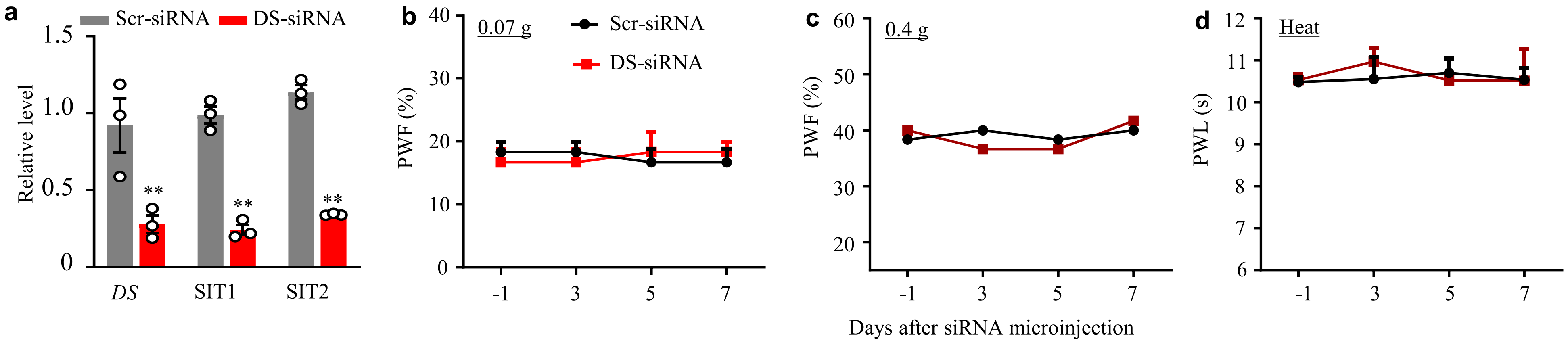

Supplementary Figure 15. Effect of DRG microinjection of *DS-lncRNA* siRNA on basal paw withdrawal responses to mechanical, heat and cold stimuli on the contralateral side in naive mice. (**a**) Levels of *DS-lncRNA* (*DS*), *SIT1* and *SIT2* in the cultured DRG neurons 2 days after transfection of *DS-lncRNA* (*DS*) siRNA (DS-siRNA) or control scrambled siRNA (Scr-siRNA). n = 3 biological repeats/group. $^{**}P < 0.01$ versus the corresponding control scrambled siRNA group by two-tailed unpaired Student's t test. (**b-d**) Paw withdrawal frequency (PWF) to low (0.07 g; b) and medium (0.4 g; c) force von Frey filaments and paw withdrawal latencies (PWL) to heat (d) stimulation on the contralateral side at days indicated after microinjection of *DS-lncRNA* siRNA or control scrambled siRNA into the unilateral L3/4 DRGs of naïve mice. n = 10 mice/group. Two-way ANOVA with repeated measures followed by post hoc Tukey test.

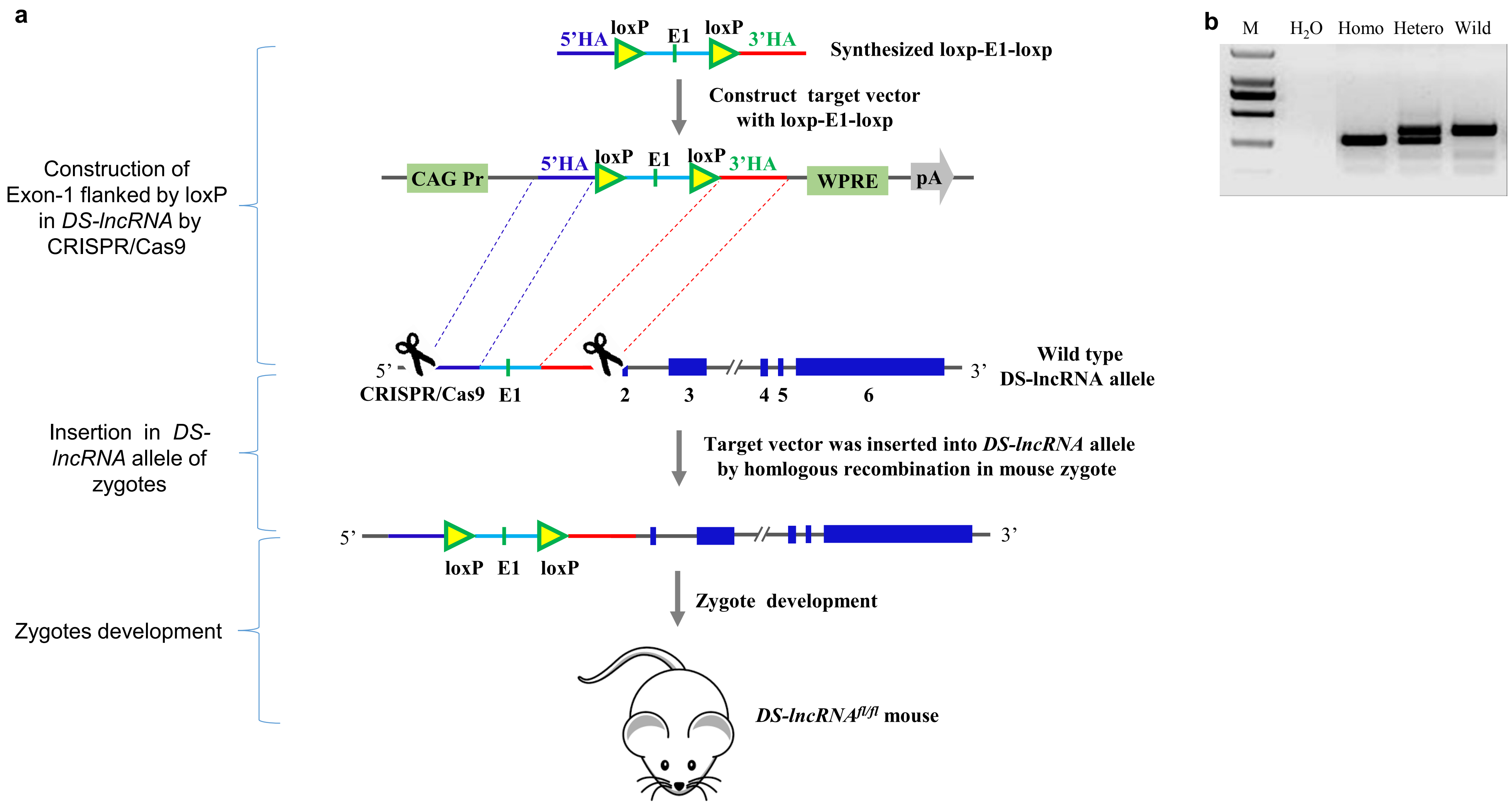

Supplementary Figure 16. Strategy for generation of *DS-lncRNA*$^{fl/fl}$ mice. (**a**) Constructed vector containing exon-1 flanked by loxP in *DS-lncRNA* gene was inserted into *DS-lncRNA* allele by homologous recombination in C57BL/6 mouse. HA, homologous arm. CAG Pro: CAG promoter in vector. pA: poly A. E1: *DS-lncRNA* exon 1. (**b**) Genotyping identification of *DS-lncRNA*$^{fl/fl}$ mice by PCR. M: DNA ladder marker. Homo: Homozygous. Hetero: Heterozygous. Wild: wild type. H_2O: without DNA template.

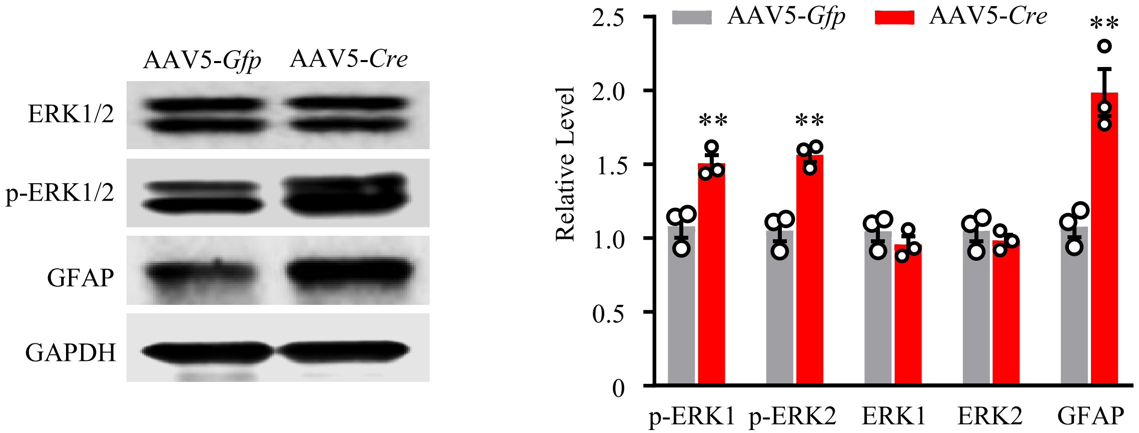

Supplementary Figure 17. Effect of DRG *DS-lncRNA* reduction through microinjection of AAV5-Cre into unilateral L3/4 DRGs of *DS-lncRNA*$^{fl/fl}$ mice on dorsal horn neuronal and astrocyte hyperactivities in naïve mice. Levels of p-ERK1/2, ERK1/2 and GFAP in the ipsilateral L3/4 dorsal horn 8 weeks after microinjection of AAV5-Cre or AAV5-*Gfp* into unilateral L3/4 DRGs of *DS-lncRNA*$^{fl/fl}$ mice. n = 6 mice/group. $^{**}P < 0.01$ versus the corresponding AAV5-*Gfp*-treated group by two-tailed unpaired Student's t test.

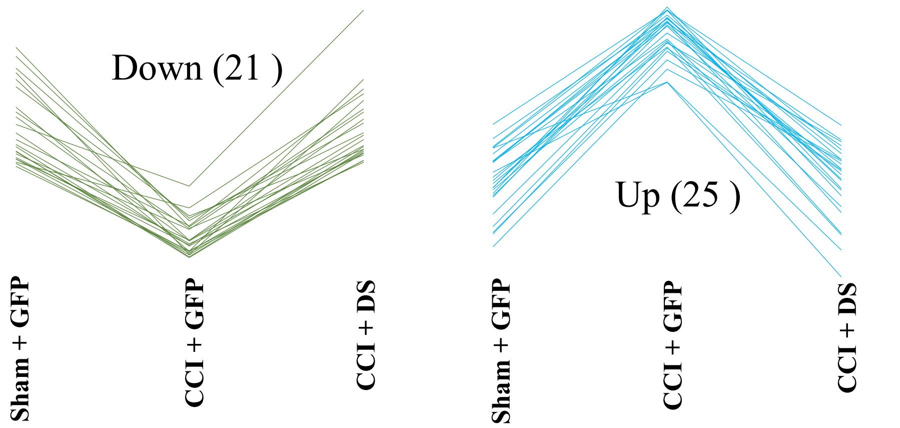

Supplementary Figure 18. Effect of rescuing nerve injury-induced *DS-lncRNA* downregulation on CCI-induced changes in gene expression in injured DRG. Up: upregulated genes. Down: down-regulated genes. The 46 differentially expressed genes reversed by *DS-lncRNA* overexpression in the ipsilateral L3/4 DRG on day 7 after CCI or sham surgery in the mice pre-microinjected with HSV-*DS-lncRNA* (DS) or HSV-*Gfp* (GFP) for 2 days.

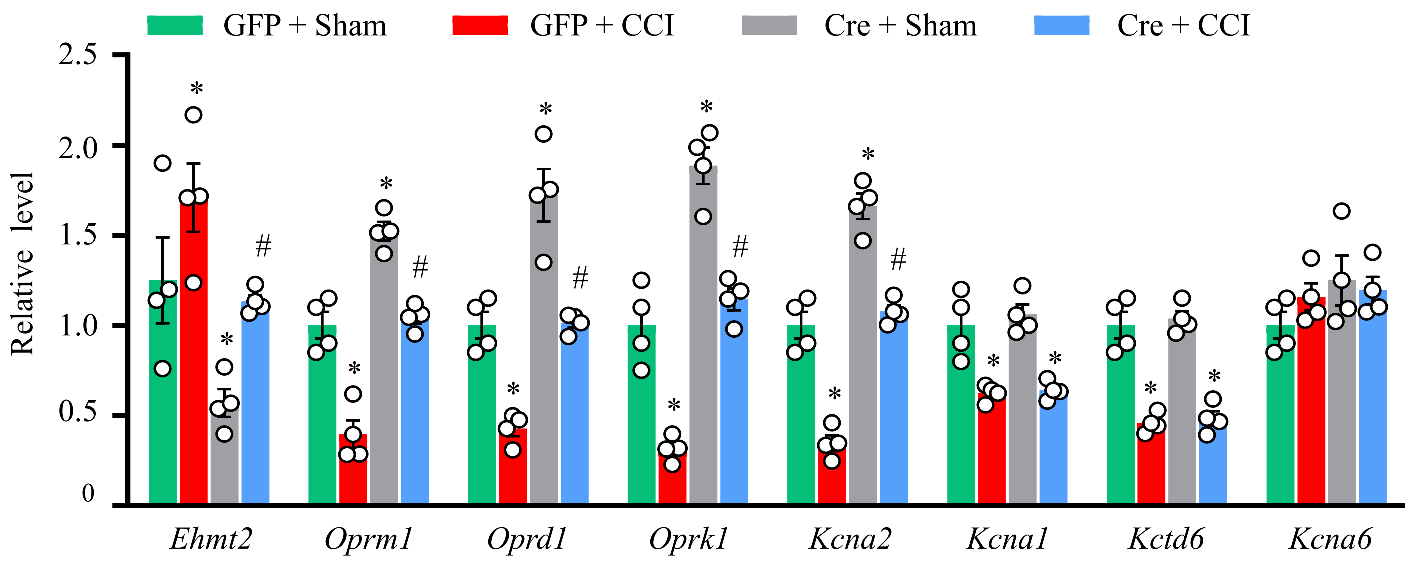

Supplementary Figure 19. Effect of DRG *DS-lncRNA* overexpression on the CCI-induced changes of pain-associated genes in the injured DRG. Levels of *Ehmt2*, *Oprm1*, *Oprmd1*, *Oprmk1*, *Kcna2*, *Kcan1*, *Kctd6* and *Kcna6* mRNAs in the ipsilateral L3/4 DRGs 14 days after CCI or sham surgery in conditional Rosa26$^{DS\text{-}lncRNA}$ knock-in (DS-KI) mice pre-microinjected with AAV5-*Cre* (Cre) or AAV5-*Gfp* (GFP) into the unilateral L3/4 DRGs. n = 8 mice/group. **P* < 0.05 versus the AAV5-*Gfp* plus sham group and #*P* < 0.05 versus the AAV5-*Gfp* plus CCI group by one-way ANOVA followed by post hoc Tukey test.

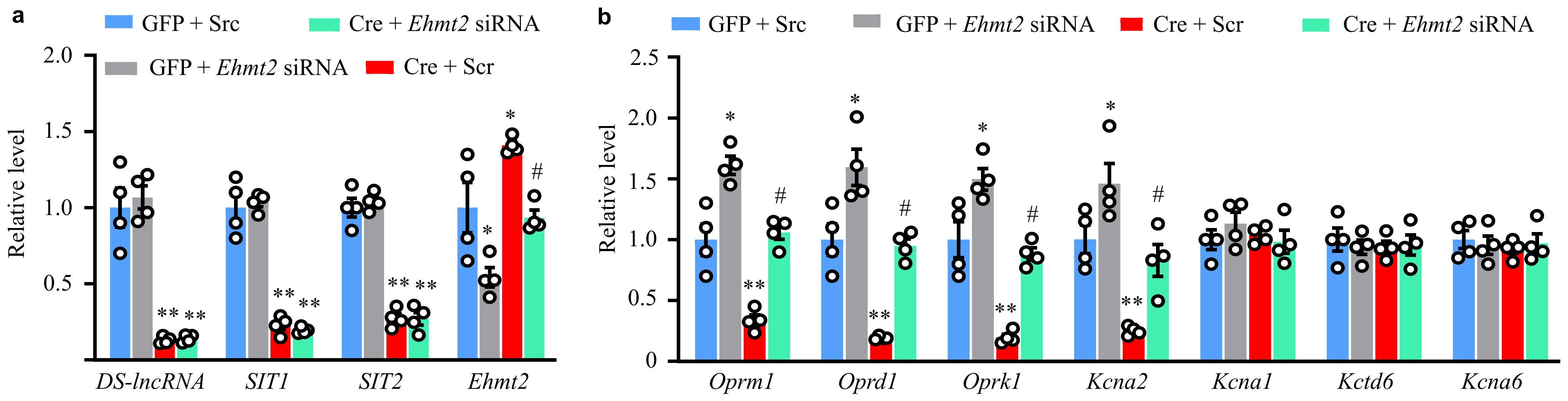

Supplementary Figure 20. Effect of DRG *Ehmt2* knockdown on the downregulation of pain-associated genes caused by *DS-lncRNA* reduction in the microinjected DRG of naive *DS-lncRNA*$^{fl/fl}$ mice. (**a**, **b**) Levels of *DS-lncRNA*, *SIT1*, *SIT2* and *Ehmt2* (a) as well as *Oprm1*, *Oprmd1*, *Oprmk1*, *Kcna2*, *Kcan1*, *Kctd6* and *Kcna6* mRNAs (b) in the ipsilateral L3/4 DRGs on day 6 after microinjection of *Ehmt2* siRNA or control scrambled siRNA (Src) into the ipsilateral L3/4 DRGs in *DS-lncRNA*$^{fl/fl}$ mice pre-microinjected with AAV5-*Cre* (Cre) or AAV5-*Gfp* (GFP) into the unilateral L3/4 DRGs for 8 weeks. n = 8 mice/group. *$P < 0.05$, **$P < 0.01$ versus the AAV5-*Gfp* plus control scrambled siRNA group and #$P < 0.05$ versus the AAV5-*Gfp* plus *Ehmt2* siRNA group by one-way ANOVA followed by post hoc Tukey test.

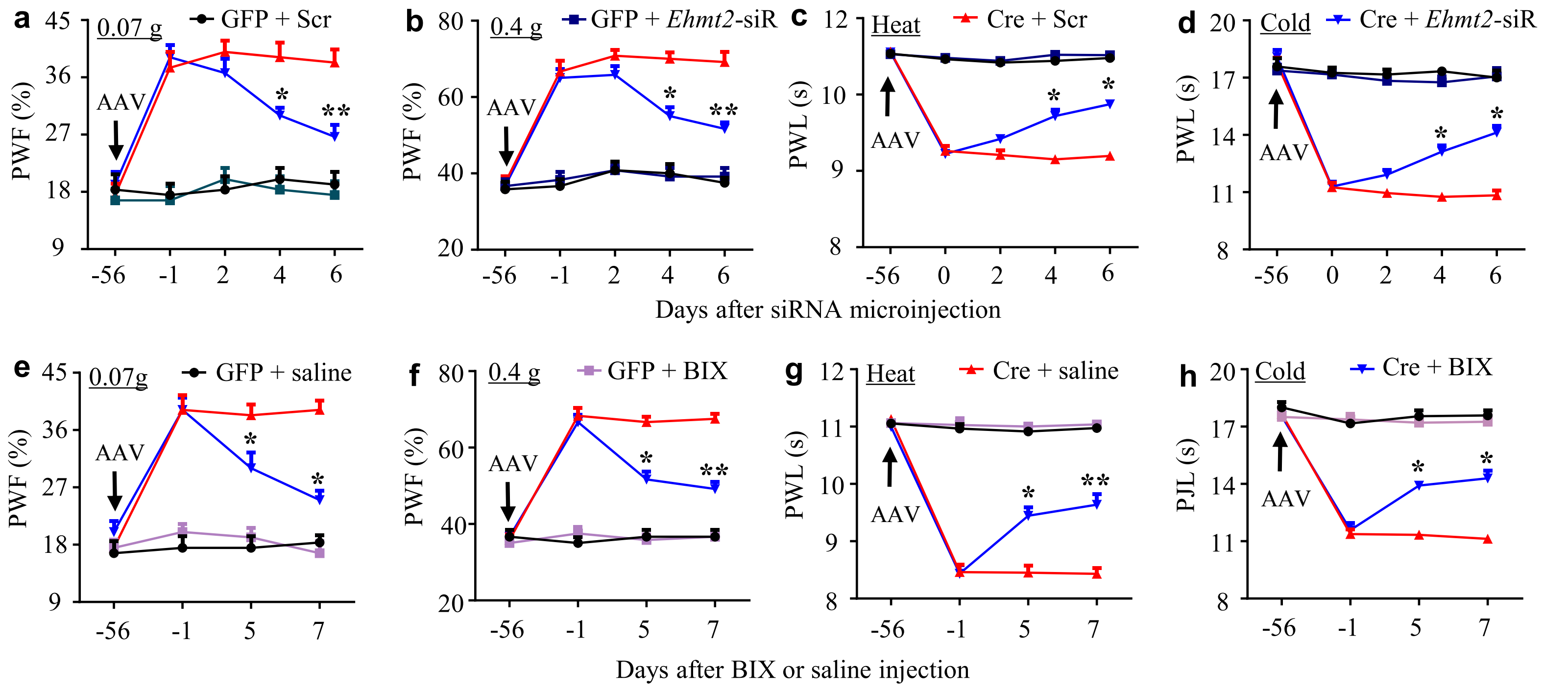

Supplementary Figure 21. Effect of DRG G9a knockdown or pharmacological inhibition on nociceptive hypersensitivities caused by DRG *DS-lncRNA* knockdown. (**a-d**) Paw withdrawal frequency (PWF) to low (0.07 g; a) and medium (0.4 g; b) force von Frey filaments and paw withdrawal latencies (PWL) to heat (c) and cold (d) stimuli on the ipsilateral side at the different days as indicated after microinjection of *Ehmt2* siRNA (*Ehmt2* siR) or control scrambled siRNA (Src) into the ipsilateral L3/4 DRGs from *DS-lncRNA*$^{fl/fl}$ mice pre-microinjected with AAV5-*Cre* (Cre) or AAV5-*Gfp* (GFP) into the unilateral L3/4 DRGs for 56 days. n = 12 mice/group. **P* < 0.05, ***P* < 0.01 versus the AAV5-*Cre* plus control scrambled siRNA group at the corresponding time points by two-way ANOVA with repeated measures followed by post hoc Tukey test. (**e-h**) Paw withdrawal frequency (PWF) to low (0.07 g; e) and medium (0.4 g; f) force von Frey filaments and paw withdrawal latencies (PWL) to heat (g) and cold (h) stimuli on the ipsilateral side at the different days as indicated after intraperitoneal injection of BIX01294 (BIX, a G9a inhibitor; 1 mg/kg) or control saline once daily for 5 days starting from 56 days after microinjection of AAV5-*Cre* (Cre) or AAV5-*Gfp* (Gfp) into the unilateral L3/4 DRGs of *DS-lncRNA*$^{fl/fl}$ mice. n = 12 mice/group. **P* < 0.05, ***P* < 0.01 versus the AAV5-*Cre* plus saline group at the corresponding time points by two-way ANOVA with repeated measures followed by post hoc Tukey test.

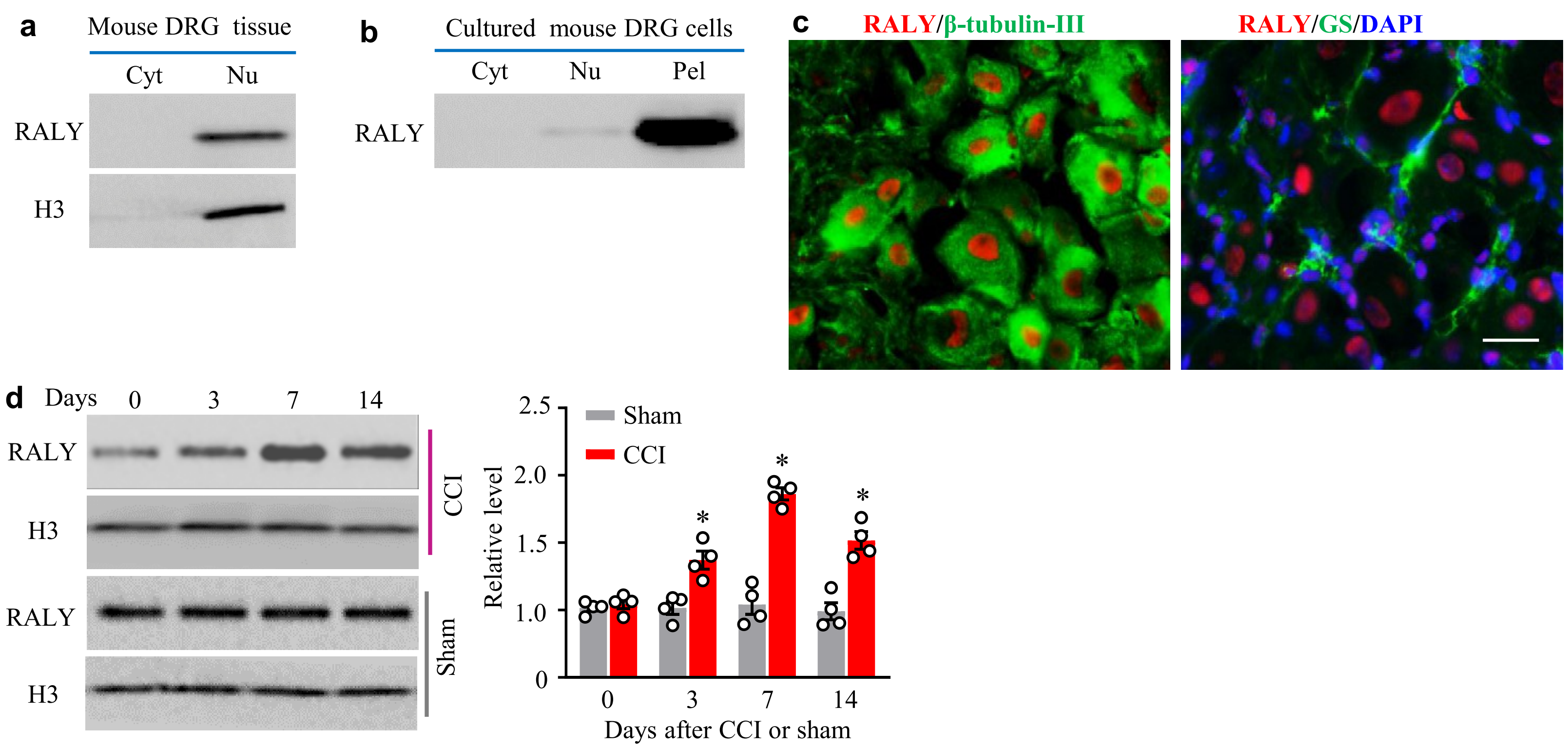

Supplementary Figure 22. Subcellular distribution of DRG RALY and an increase in its expression in injured DRG after CCI. (**a**) Expression of RALY in cytoplasmic (Cyt) and nuclear (Nu) fractions, respectively, from adult mouse DRG. n = 3 repeats. (**b**) Identification of RALY expression in cytoplasmic soluble (Cyt), nuclear soluble (Nuc) and insoluble pellet (Pel) fractions, respectively, from cultured DRG neurons. (**c**) RALY (red) is located predominantly in the nuclei of the β-tubulin III (green, left)-positive individual cells and weakly in cellular nuclei (labeled by 4', 6-diamidino-2-phenylindole (DAPI), blue) of glutamine synthetase (GS, green, right)-labeled cells in naive DRG. Approximate 45 % of the β-tubulin III-labelled neurons were positive for Pou4f3. n = 3 mice. Scale bar: 25 µm. (**d**) Time-course expression analysis of Pou4f3 protein in the ipsilateral L3/4 DRG after CCI or sham surgery. n = 8 mice/time point/group. *$P < 0.05$ versus the corresponding sham group (0 day) by two-way ANOVA with repeated measures followed by post hoc Tukey test.

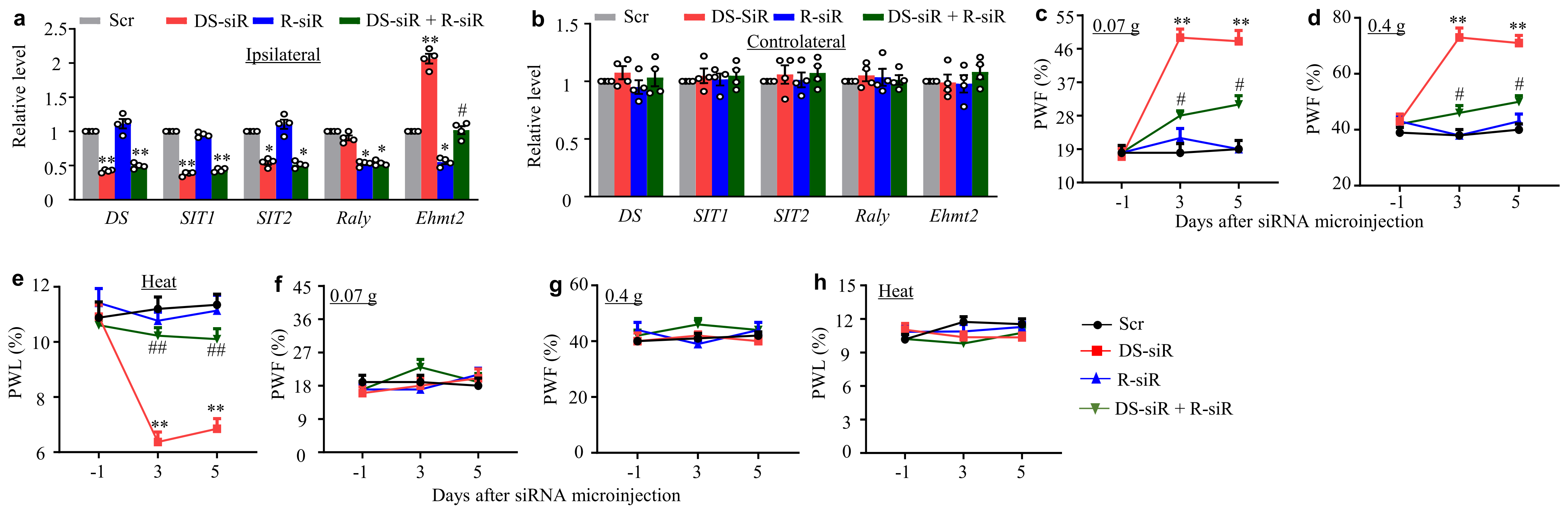

Supplementary Figure 23. DRG knockdown of Raly inhibited nociceptive hypersensitivities caused by the downregulation of DRG *DS-lncRNA*. (**a-b**) Levels of *DS-lncRNA* (*DS*), *SIT1, SIT2, Raly* mRNA and *Ethm2* mRNA in the ipsilateral (**a**) and contralateral (**b**) L3/4 DRGs on day 5 after microinjection with *DS-lncRNA* siRNA (DS-siR; 40 µM; 1 µl), Raly-siRNA (R-siR; 40 µM; 1 µl), *DS-lncRNA* siRNA plus Raly-siRNA, or control scrambled siRNA (Scr; 40 µM; 1 µl) into unilateral L3/4 DRGs. n = 8 mice/group. *$P < 0.05$, **$P < 0.01$ versus the corresponding scrambled siRNA-treated mice. $^{\#}P < 0.05$ versus the corresponding *DS-lncRNA* siRNA-treated mice. One-way ANOVA followed by post hoc Tukey test. (**c-h**) Effect of microinjection of *DS-lncRNA* siRNA (DS-siR), Raly-siRNA (R-siR), *DS-lncRNA* siRNA plus Raly-siRNA, or control scrambled siRNA (Scr) into unilateral L3/4 DRGs on the paw withdrawal frequencies (PWF) to 0.07 g (**c**, **f**) and 0.4 g (**d**, **g**) von Frey filaments and on paw withdrawal latencies (PWL) to heat (**e**, **h**) stimuli on the ipsilateral (**c-e**) and contralateral (**f-h**) sides at the different days after siRNA microinjection. n = 10 mice/group. **$P < 0.01$ versus the control scrambled siRNA-treated mice at the corresponding time point. $^{\#}P < 0.05$, $^{\#\#}P < 0.01$ versus the *DS-lncRNA* siRNA-treated mice at the corresponding time points. Two-way ANOVA with repeated measures followed by post hoc Tukey test.

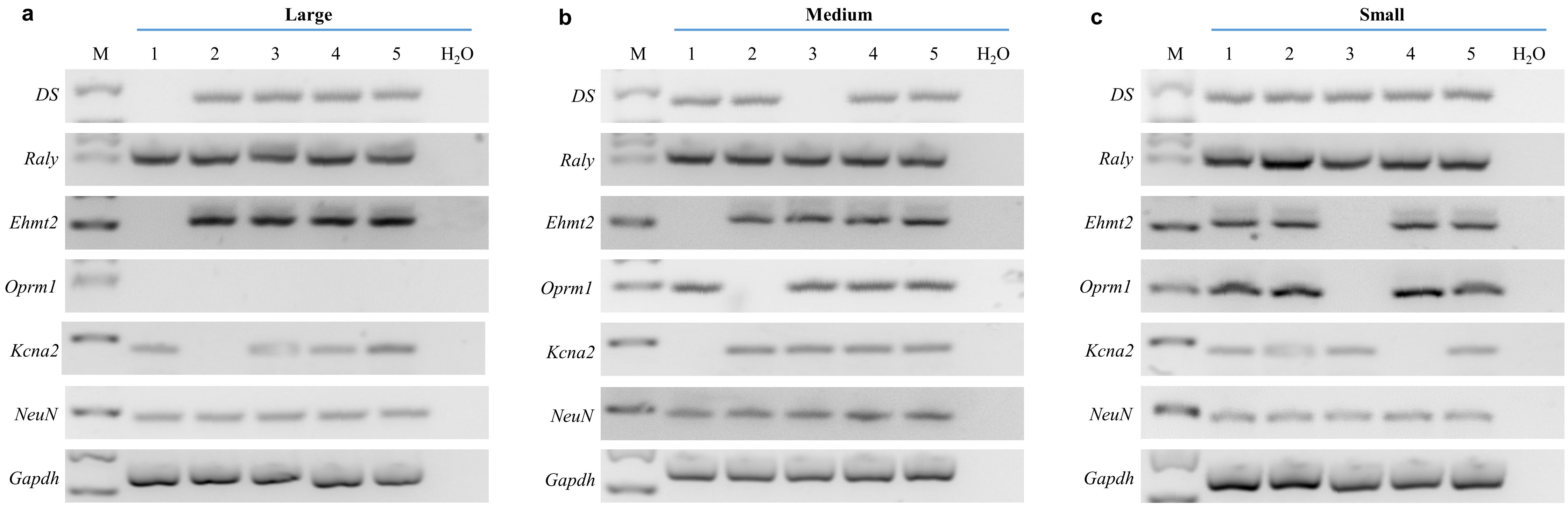

Supplementary Figure 24. Co-expression analysis of *DS-lncRNA* (DS), *Raly* mRNA, *Ehmt2* mRNA, *Oprm1* mRNA and *Kcna2* mRNA in individual dorsal root ganglion (DRG) neurons by single-cell RT-PCR assay. *NeuN* mRNA is used as a marker for DRG neurons. *Gapdh* mRNA was used a loading control. (**a**) Co-localization of *DS-lncRNA* with *Raly*, *Ehmt2* and *Kcna2* mRNAs in some individual large DRG neurons (> 35 µm in diameter). *Oprm1* mRNA was undetectable in large DRG neurons. (**b**) Co-localization of *DS-lncRNA* with *Raly, Ehmt2, Kcna2* and *Oprm1* mRNAs in some individual medium DRG neurons (25-35 µm in diameter). (**c**) Co-localization of *DS-lncRNA* with *Raly, Ehmt2, Kcna2* and *Oprm1* mRNAs in some individual small DRG neurons (< 25 µm in diameter). n = 5 neurons/size. Number 1–5 indicates five different neurons. M: DNA ladder marker. H_2O, no cDNA.

Supplementary Table 1. Mean changes in locomotor function.

Treatment groups	Placing	Grasping	Righting
HSV-Gfp + Sham	5(0)	5(0)	5(0)
HSV-Gfp + CCI	5(0)	5(0)	5(0)
HSV-DS-lncRNA + Sham	5(0)	5(0)	5(0)
HSV-DS-lncRNA + CCI	5(0)	5(0)	5(0)
CCI + HSV-Gfp	5(0)	5(0)	5(0)
CCI + HSV-DS-lncRNA	5(0)	5(0)	5(0)
AAV5-Gfp + Sham	5(0)	5(0)	5(0)
AAV5-Gfp + CCI	5(0)	5(0)	5(0)
AAV5-Cre + Sham	5(0)	5(0)	5(0)
AAV5-Cre + CCI	5(0)	5(0)	5(0)
Scramble siRNA	5(0)	5(0)	5(0)
DS-lncRNA siRNA	5(0)	5(0)	5(0)
AAV5-Gfp	5(0)	5(0)	5(0)
AAV5-Cre	5(0)	5(0)	5(0)
AAV5-Pou4f3 + Sham	5(0)	5(0)	5(0)
AAV5-Pou4f3 + CCI	5(0)	5(0)	5(0)
Pou4f3-siRNA	5(0)	5(0)	5(0)
AAV5-Gfp + SNL	5(0)	5(0)	5(0)
AAV5-Cre + SNL	5(0)	5(0)	5(0)
AAV5-Gfp + Scramble siRNA	5(0)	5(0)	5(0)
AAV5-Gfp + Ehmt2 siRNA	5(0)	5(0)	5(0)
AAV5-Cre + Scramble siRNA	5(0)	5(0)	5(0)
AAV5-Cre + Ehmt2 siRNA	5(0)	5(0)	5(0)
AAV5-Gfp + Saline	5(0)	5(0)	5(0)
AAV5-Gfp + BIX	5(0)	5(0)	5(0)
AAV5-Cre + Saline	5(0)	5(0)	5(0)
AAV5-Cre + BIX	5(0)	5(0)	5(0)

Supplementary Table 2. All primers and probes used

Names	Sequences
RT-PCR	
Mouse-DSF	5′- GGCTTACATCCTGTCTGTGTGA-3′
Mouse-DSR	5′-CCAACAATGGCGTTCATAGAC-3′
Human-DSF	5′-ATTCCCATCTGTGACTTGATAA-3′
Human-DSR	5′-AATACTTAGGTTGCTGAGAGC-3′
Human-GAPDHF	5′-AGGTCGGAGTCAACGGATTTG-3′
Human-GAPDHR	5′-GTGATGGCATGGACTGTGGT-3′
Vector construction	
HSV-DSF	5′-GCAGGCTCCGCGGCCGAAGCGAAGCGCTCCAGACG-3′
HSV-DSR	5′-AAGCTGGGTCGGCGCAAGATTTCACACATATTACATAAATC-3′
AAV- Pou4f3F	5′-ATATCCGGAGCCACCATGATGGCCATGAACGCC-3′
AAV-Pou4f3R	5′-ATAGCGGCCGCTCAGTGGACAGCAGAGTATT-3′
AAV-RalyF	5′-ATATCCGGAGCCACCATGATGGCCATGAACGCC-3′
AAV-RalyR	5′-ATAGCGGCCGCTCAGTGGACAGCAGAGTATT-3′
pGL3-DSF	5′-ATAGGTACCACTCAAGGCTATCCCTGGA-3′
pGL3-DSR	5′-ATAGCTAGCCCTCGTCTGCAGCCCGGCT-3′
Northern/ISH	
N-DS-SF	5′-TAATACGACTCACTATAGGGTAAGGCGAGAGGGGATTTTC-3′
N-DS-SR	5′-ATCTCCCCAGCACCTCTTTT-3′
N-DS-ASF	5′-TAAGGCGAGAGGGGATTTTC -3′
N-DS-ASR	5′-TAATACGACTCACTATAGGGATCTCCCCAGCACCTCTTTT-3′
In vitro protein translation	
DS-T7F	5′-TAATACGACTCACTATAGGGGAAGCGAAGCGCTCCAGAC-3′
DS-T7R	5′-CACTATGTAAAATAATATTTTTATTAA-3′
Luciferase-T7F	5′-TAATACGACTCACTATAGGGATG GAAGACGCCAAAAACATAA-3′
Luciferase-T7R	5′-TTACACGGCGATCTTTCCGCCCTT-3′
Creb1-T7F	5′-TAATACGACTCACTATAGGGATGCCAGCTCATGCAAC-3′
Creb1-T7R	5′-TTAATCTGATTTGTGGCAGTAAAG-3′
H19-T7F	5′-TAATACGACTCACTATAGGGACCGGGTGTGGGAGGGGGGT-3′
H19-T7R	5′-ATGACTGTAACTGTATTTATTGAT-3′
ChIP-PCR	
ChIP-Pou4f3F	5′-ACTTGCGCAACCATCCCAG-3′
ChIP-Pou4f3R	5′-CGTCTGGAGCGCTTCGCTTC-3′
ChIP-G9aF	5′-CAAAAGCACACTGACCCAGA-3′
ChIP-G9aR	5′- CACGTGTAGCCTGAGCCTTT -3′
ChIRP probes (O, Odd probe; E, Even probe)	
DS-O1	5′-bio-TATCCACATGTATGCAGGAT-3′
DS-E2	5′-bio-CTTAAGTTCTCACTGCAGGA -3′
DS-O3	5′-bio-CACTTGACCCAACCAGAAAC-3′
DS-E4	5′-bio-AACTCAACAGGACCTTTGCA-3′
DS-O5	5′-bio-ATCTTCACAGTTATTTTCCC-3′
DS-E6	5′-bio-TGGTGAATCTGGGAAGGAAG-3′
DS-O7	5′-bio-TCATGCTTTCACGGAAAGCA-3′
DS-E8	5′-bio-TTGGAGCCCTTTGAGTATTA-3′
DS-O9	5′-bio-GTTGACAAAGAGCTAGCCAG-3′
DS-E10	5′-bio-GTAGACATGGGCTTTGAGAT-3′
DS-O11	5′-bio-CCATAGGCTCATATAGTTGT-3′
DS-E12	5′-bio-TGCTTTCGATAACGGGGATT-3′
DS-O13	5′-bio-GGCCACTTCAAATGTACTTG-3′
DS-E14	5′-bio-AGCCAGGGATTTACAGAGAA-3′
DS-O15	5′-bio-CAAAAGCAGCCATGAGCTTA-3′
DS-E16	5′-bio-ATTCATCTGCTTTGGGTATC-3′
Negative	5′-bio-CCAGTGAATCCGTAATCATG-3′
RACE extended primers	
DS-SF1	5′-GAAGCGAAGCGCTCCAGACG-3′
DS-SR1	5′-TGCTGAGAGCTGTGCAGTTT-3′
DS-SF2	5′-GGCCAGTCAACATCCCTACA-3′
DS-SR2	5′-TGGACTCACCCTCTGAAACC-3′
DS-SF3	5′-GGTTTCAGAGGGTGAGTCCA-3′
DS-SR3	5′-TACCTTCATCTGCCCAGCTT-3′
DS-SF4	5′-TTAGGGACACTGGGATCAGC-3′
DS-SR4	5′-AAGCTGGGCAGATGAAGGTA-3′
RACE 5′-end primers	
SP1 for RT	5′-ACATGGCCTCAGGAAAACAC-3′
5′ Oligo Td-Anchor	Provided by 5′/3′ RACE kit (03353621001)
SP2 for the first nested PCR	5′-GCATCCTTAGAGGTAAATGTTGC-3′
5′ PCR anchor	Provided by 5′/3′ RACE kit (03353621001)
SP3 for the second nested PCR	5′-AAAGGCGCGCCCGTCTGGAGC-3′
RACE 3′-end primers	
3′ end RT Td-Anchor primer	Provided by 5′/3′ RACE kit (03353621001)
The first nested PCR 3SP1F	5′-GCTGATCCCAGTGTCCCTAA-3′
The second nested PCR 3SP2F	5′-TGTCTGATACCCAAAGCAGATG-3′
The first/second nested PCR anchor	Provided by 5′/3′ RACE kit (03353621001)
Real-time RT-PCR	
Total-mDSF	5′- TTCGTTGGTTTCCCATCTTC-3′
Total-mDSR	5′-CGCTGATCCTCTGGTCTAGG-3′
SIT1/SIT2 RT primer	5′-ATACCACCCAGGACCATCTG-3′
mSIT1F	5′-GTCAACCATGATGGGGTAGC-3′
mSIT1R	5′-TGCTGAGAGCTGTGCAGTTT-3′
mSIT2F	5′-GGCTTACATCCTGTCTGTGTGA-3′
mSIT2R	5′-AGAAATACTTAGGCTAAGGAATA-3′
mRalyF	5′-ATTCAGACCAGCAATGTAACCAA-3′
mRalyR	5′-CACAGAGCAACCAGCCACT-3′
mPou4f3F	5′-ATGCGCCGAGTTTGTCTCC-3′
mPou4f3R	5′-GGGCTTGAACGGATGGTTCT-3′
mG9aF	5′-AAATTGGGAACTTGGAAATGG-3′
mG9aR	5′-CACTACCCGTGAAGGAGGC-3′
mDNMT1F	5′-AGTCGGACAGTGACACCCTTT-3′
mDNMT1R	5′-TGTGTCTACAACTCTGCGTTTCT-3′
mDNMT3aF	5′-CGAATTGTGTCTTGGTGGATGAC-3′
mDNMT3aR	5′-GGTGGAATGCACTGCAGAAGGA-3′
mMBD1F	5′ -CTGCATCTGCGTCTTCACAT-3′
mMBD1R	5′-CACACCCCACAGTCCTCTTT-3′
mCEBPbF	5′ -CAAGCTGAGCGACGAGTACA-3′
mCEBPbR	5′-CAGCTGCTCCACCTTCTTCT-3′
mOprm1F	5′-TCTTCACCCTCTGCACCATG-3′
mOprm1R	5′-TCTATGGACCCCTGCCTGTA-3′
mOprd1F	5′-TTGGCATCGTCCGGTACAC-3′
mOprd1R	5′-AGAGCACAGCCTTGCACAGC-3′
mOprk1F	5′-AGTGTGGACCGCTACATTGCT-3′
mOprk1R	5′-CAATGACATCCACATCTTCCCTG-3′
mKv1.2F	5′-CTGCAAGGGCAACGTCACAC-3′
mKv1.2R	5′-GGGACAGTGAGATGCTTGGC-3′
mKcna1F	5′-GTGATGTCGGGGGAGAATGTT-3′
mKcna1R	5′-CCGGAGATGTTGATTACTACGC-3′
mKctd6F	5′-ATGGGGACTGGGGCTATATGA-3′
mKctd6R	5′-GAATCCGGGTAGCGTGTCAAC-3′
mKcna6F	5′-AGATCGGAGAAATCCCTGACG-3′
mKcna6R	5′-CCAACCTCTCACTACTACAGCA-3′
mTuba1aF	5′-GTGCATCTCCATCCATGTTG-3′
mTuba1aR	5′-GTGGGTTCCAGGTCTACGAA-3′
mGAPDHF	5′-AGGCCGGTGCTGAGTATGTC-3′
mGAPDHR	5′-TGCCTGCTTCACCACCTTCT-3′
Single cell RT-PCR	
DSoutF	5′-GTGTTTTCCTGAGGCCATGT-3′
DSoutR	5′-CGCTGATCCTCTGGTCTAGGT-3′
DSinF	5′-CAAGTGATGCAGACTCAAATG-3′
DSinR	5′-GTGAGGGAGAGACCTGGTGA-3′
Raly-outF	5′-GGCTTCCTCCAGACCTCG-3′
Raly-outR	5′-GCTCATTGGCATACTGGACA-3′
Raly-inF	5′-ATTCAGACCAGCAATGTAACCAA-3′
Raly-inR	5′-CACAGAGCAACCAGCCACT-3′
G9a-outF	5′-GTGGGTGAAGCCATCTAGAA-3′
G9a-outR	5′-CAGCCCAGGAGCTCTCCAT-3′
G9a-inF	5′-AGCCAAGAGGGGTCTCCAAT-3′
G9a-inR	5′-CTCGCTGATGCGGTCAATCT-3′
Oprm1-outF	5′-ACAGCCTACCGAGTCCGCA-3′
Oprm1-out R	5′-GAGGGCACAGGCTGTGGCT-3′
Oprm1-inF	5′-CCAGGGAACATCAGCGACTG-3′
Oprm1-inR	5′-GTTGCCATCAACGTGGGAC-3′
Po4f3-outF	5′-ACTGCAAGAACCCAAATTCTC-3′
Po4f3-outR	5′-GTTGGAACAGACGTGTGGAT-3′
Pou4f3-inF	5′- ATGCGCCGAGTTTGTCTCC-3′
Pou4f3-inR	5′-GGGCTTGAACGGATGGTTCT-3′
KV1.2-outF	5′-CTGCAAGGGCAACGTCACAC-3′
KV1.2-outR	5′-GGGACAGTGAGATGCTTGGC-3′
KV1.2-inF	5′-AGGACTCAGGCTTTTGCTGA-3′
KV1.2-inR	5′-GTGGAGCTTGGGTCTGAAG-3′
siRNA	
si-DS-S	5′-GCUAUUUACCUAUAUACUATT-3′
si-DS-AS	5′-UAGUAUAUAGGUAAAUAGCAG-3′
si-Raly-S	5'-UCUACAGGCUGUUUGAUUATT-3'
si-Raly-AS	5'-AAUAAUCAAACAGCCUGUAGATT-3'

RT: Reverse-transcription; F, Forward; R, Reverse.

Supplementary Table 3. DS-lncRNA binding proteins by the use of ChIRP-MS.

Proteins	Acc. No.	M. W.	Repeat-1 (Ratio)		Repeat-2 (Ratio)		Repeat-3 (Ratio)	
			Even/NC	Odd/NC	Even/NC	Odd/NC	Even/NC	Odd/NC
Ywhah	P68510	28 kDa	1.1	1.3	1.3	1.4	1.3	1.2
Ywhaq	P68254	28 kDa	1.3	1.6	0.58	0.6	1.3	1.3
Mpst	Q99J99	33 kDa	1.5	1.5	1.4	1.6	1.5	1.5
Arf5	P84084	21 kDa	1.3	2.0	0.7	0.9	1.3	1.1
Pfkp	Q9WUA3	85 kDa	2.0	1.3	1.8	1.7	1.8	1.7
Cops7a	Q9CZ04	30 kDa	1.5	1.5	1.3	1.5	2.3	2.0
Lap3	Q9CPY7	56 kDa	1.3	1.8	2	1.8	1.2	1.2
Ddost	O54734	49 kDa	1.2	1.2	1.25	1.5	1.2	1.3
Ehd4	Q9EQP2	61 kDa	1.7	1.7	1.3	1.1	1.3	1.1
Echs1	Q8BH95	31 kDa	1.3	2.3	1.2	0.6	1.4	1.1
Fdps	Q920E5	41 kDa	1.2	1.6	1.3	1.3	1.2	1.2
Ssb	P32067	48 kDa	1.3	1.3	1.2	1.4	1.3	1.4
Nudc	O35685	38 kDa	1.3	1.3	1.2	1	1.4	1.8
Nol3	Q9D1X0	25 kDa	1.3	1.7	1.7	1.3	1.3	1.2
Ppid	Q9CR16	41 kDa	1.7	1.7	1.3	1.3	2.7	1.3
Fkbp4	P30416	52 kDa	1.3	2.3	1.4	2	2.0	1.7
Fkbp8	O35465	44 kDa	1.5	1.5	1.5	2.3	1.3	2.0
Ppp1r12a	Q9DBR7	115 kDa	1.5	1.5	1.5	1.5	1.5	1.5
Cct5	P80316	60 kDa	1.3	1.3	0.8	0.9	1.5	1.4
Cct3	P80318	61 kDa	1.3	2.2	1.1	1.4	1.2	1.5
Hadhb	Q99JY0	51 kDa	1.1	1.3	1.3	1.2	1.2	1.4
Nsun2	Q1HFZ0	85 kDa	2.0	1.0	0.5	0.5	1.2	1.2
Usp5	P56399	96 kDa	1.4	1.3	1.2	1.3	1.3	1.4
Raly	Q64012	33 kDa	2.7	2.7	2.5	2.5	2.5	2.8
